# Bu-Gu-Sheng-Sui decoction promotes osteogenesis *via* activating the ERK/Smad signaling pathways

**DOI:** 10.3389/fphar.2022.976121

**Published:** 2022-08-25

**Authors:** Ning Liu, Baoyu Qi, Yili Zhang, Shengjie Fang, Chuanrui Sun, Qiuyue Li, Xu Wei

**Affiliations:** ^1^ Wangjing Hospital, China Academy of Chinese Medical Sciences, Beijing, China; ^2^ School of Traditional Chinese Medicine and School of Integrated Chinese and Western Medicine, Nanjing University of Chinese Medicine, Nanjing, China

**Keywords:** Bu-Gu-Sheng-Sui decoction, osteogenesis, mechanism, ERK/Smad, osteoporosis

## Abstract

Osteoporosis is a systemic metabolic skeletal disease, which becomes a common public health problem that seriously endangers people’s health. Bu-Gu-Sheng-Sui decoction (BGSSD) is a safe and effective Chinese medicine formulation for the treatment of osteoporosis. Numerous studies have indicated that it played a significant role in bone anabolism. However, the underlying mechanism remains unclear. Herein, we selected senescence-accelerated mice prone 6 (SAMP6) and MC3T3-E1 cells to study the effects of BGSSD on osteogenesis and then investigated the potential mechanism of BGSSD. Our research found that BGSSD protected the bone mass in SAMP6, increased the expression of osteogenic specific factor Runx2, and improved bone trabecular structure. *In vitro*, BGSSD accelerated the proliferation and differentiation of MC3T3-E1 cells, which was characterized by stimulating the activity of Alkaline phosphatase (ALP) and raising the expression of Runx2. Moreover, BGSSD could effectively boost the expression levels of ERK and Smad in SAMP6 and MC3T3-E1. Therefore, we speculate that BGSSD may promote bone formation through ERK/Smad pathways. Collectively, our results highlight the importance of BGSSD as a compound in promoting osteogenic differentiation and osteogenesis, demonstrating that BGSSD may become a latent drug to prevent and treat osteoporosis.

## Introduction

Osteoporosis (OP) is a systemic metabolic skeletal disease characterized by decreased bone mineral density (BMD), bone mass loss, and destructed microstructure of bones, easily leading to bone fragility and a high probability of fracture (Johnston and Dagar, 2020). OP is one of the age-related bone diseases in which bone loss progresses with advancing age. With the advent of an aging society, OP has become a common public health problem that seriously endangers people’s health in various countries (Noh et al., 2020). Especially fractures caused by OP seriously affect the patient’s quality of life and increase the mortality rate (Compston et al., 2019). The pathogenesis of OP is complicated, yet more bone resorption than formation is the primary pathological manifestation (Wong et al., 2019). The current clinical treatment of OP mainly focuses on inhibiting bone resorption, yet with many side effects. Although many researchers have demonstrated that the multilevel processes of bone formation are essential for maintaining bone mass, effective drugs are still lacking (Chang et al., 2019). Therefore, looking for potential drugs for the treatment of OP, which stimulate bone formation, is an urgent problem to be solved.

Traditional Chinese medicine (TCM) has unique advantages in treating OP due to its multi-component and multi-target characteristics, especially in promoting bone formation (Zhang et al., 2016). Bu-Gu-Sheng-Sui decoction (BGSSD) is composed of eight herbs (Drynaria, Fructus Psoraleae, Rhizoma cibotii, Wolfberry, Raw Oysters, Ginseng, Panax Notoginseng, and Fructus Amomi), which has exact efficacy in treating OP. A randomized controlled trial of 80 cases manifested that the significant and overall effective rates of BGSSD in treating OP were 46% and 82%, respectively. BGSSD raised BMD, ameliorated clinical symptoms, and had reliable safety in the treatment process (Xie et al., 1997a). On the premise of good clinical efficacy, many basic studies were conducted, and the results testified that BGSSD could increase the BMD and bone biomechanical properties of ovariectomized rats (Xie et al., 1997b), modify microcirculation disorders (Liu et al., 1997) and relieve inflammation and pain (Ji et al., 1997). These results attested that BGSSD could promote osteogenesis effectively. However, the detailed mechanism of BGSSD is still unclear.

The extracellular regulated protein kinases (ERK) pathway acted a prominent role in enhancing osteoblast differentiation and promoting bone formation (Catalano et al., 2017; Mizumachi et al., 2017; Kim et al., 2018). It was the most critical event in bone development and bone mass maintenance (Bhaskar et al., 2014; Park et al., 2019). The Smad protein family also played an integral part in regulating bone metabolism (Kopf et al., 2014; Cheng et al., 2019; Feng et al., 2020). The down-regulation of Smad mRNA expression and protein level may be one of the momentous mechanisms of OP (Donoso et al., 2015; Durbano et al., 2020). Inhibition and defect of Smad signal transduction may down-regulate the expression of Runx2 and Osterix, affecting the activity of osteoblasts, leading to reduced osteogenesis and bone mass, and ultimately to the occurrence of OP (Dineen and Gaudet, 2014; Li et al., 2014; Liu et al., 2017). Studies showed that the main extracts of herbs in BGSSD affected the differentiation of osteoblasts by modifying the ERK or Smad pathway (Zhu et al., 2012; Zhang et al., 2019; Sun et al., 2021b; Huang et al., 2021). Moreover, many kinds of research have shown a mutual crosstalk relationship between ERK and Smad (Zhou et al., 2007; Park et al., 2011; Lee et al., 2018; Tashima et al., 2020). Therefore, we hypothesize that BGSSD may enhance osteoblast differentiation through ERK/Smad pathway to exert an anti-osteoporosis effect.

Based on the above, the osteogenic functions of BGSSD will be verified in SAMP6 and MC3T3-E1 cells. Furthermore, potential mechanisms of BGSSD in boosting osteogenesis through ERK/Smad will be investigated in the rat model and osteoblastic cells. The above research results may provide new pharmacological evidence for BGSSD in clinically treating or preventing osteoporosis.

## Materials and methods

### Experimental animal

The animals were purchased from the Laboratory Animal Science Department of Peking University Health Science Center, with the license number SCXK (Jing) 2020–0002. SAMP6 were randomly divided into the model group (Model, *n* = 6), high-dose BGSSD-treated group (H-BGSSD, *n* = 6), middle-dose BGSSD-treated group (M-BGSSD, *n* = 6), low-dose BGSSD-treated group (L-BGSSD, *n* = 6), and alendronate (Shi Yao, Hebei, China)-treated group (Alendronate, *n* = 6). Six senescence-accelerated mice resistant 1 (SAMR1) were used as the control group (Control, *n* = 6), alendronate was given at 1.517 mg/kg, and the low-, medium-, and high-dose of BGSSD groups were respectively given the crude drug at 10.16 g/kg, 20.32 g/kg, and 40.64 g/kg intragastrically. All animals were housed at 1 per cage in the Animal Experiment Center of the Acupuncture Institute, China Academy of Chinese Medical Sciences, with humidity of 48% and temperature of 25°C, and freed access to food and water. Two weeks of adaptive feeding were adjusted before the gavage.

### Drug preparation

Chinese herbal medicine was purchased from Wangjing Hospital of China Academy of Chinese Medical Sciences, and administered dosages were converted according to the clinically effective dose. We referenced the procedure of drug preparation by Jiang et al. (2021). All medicinal plants of BGSSD were decocted 40 min twice with boiling purified water (1:10 and then 1: 5, w/v). After filtering, the solution was concentrated using a vacuum evaporator (70°C). The solution was stored at 4°C and diluted with ultrapure water to the desired concentrations before use.

### Tissue collection

After 12 weeks, all animals were anesthetized by intraperitoneal injection of 0.3% sodium pentobarbital (Haling, Shanghai, China). Obtained the left femurs and immediately placed them in PBS (Solarbio, Beijing, China) for BMD detection. The right femurs were stored in paraformaldehyde solution (Solarbio, Beijing, China) for fixation, and the distal femoral metaphysis was selected for Hematoxylin-eosin (HE) staining. The right tibias were determined by immunohistochemistry (IHC) Staining, and the left tibias were quick-frozen with liquid nitrogen and stored at −80°C, which were used for Western Blotting (WB) and Real-time PCR (RT-PCR) detection.

### Dual-energy X-ray absorptiometry test

The skeletal imaging was performed via a dual-energy X-ray scanner (Osteocore, Medilink, France). It can automatically aim at the induction area and control quality and periosteum calibration. Cross-sectional images of the samples were collected, and the range of the femur was framed. Bone mineral mass and bone area were analyzed and calculated by Osteocore3.7.0.0.5 software. Finally, the BMD was calculated.

### Hematoxylin-eosin staining assay

Right femurs were fixed with 4% paraformaldehyde (Solarbio, Beijing, China) for 7 days, and decalcified, dehydrated, embedded in paraffin, cut into 5-μm sections, and then deparaffinized by xylene (Solarbio, Beijing, China) together with ethanol (in the following order: 100, 95, 90, 80, 70%). The sections were immersed in distilled water, and hematoxylin-eosin staining (Baso, Zhuhai, China) was performed according to the protocol. Bone trabeculae were observed under a microscope (Carl Zeiss, Germany), and bone cavity volume ratio was measured by Image-Proplus5 (IPP).

### Immunohistochemical staining assay

Tibias were fixed in 4% paraformaldehyde solution at 4°C for 24 h, and then they were placed in 10% ethylene diamine tetraacetic acid solution (Solarbio, Beijing, China) and decalcified for 21 days. Next, the bones were embedded in paraffin (Guoyao, Shanghai, China). Paraffin wax blocks were sliced into longitudinally oriented bone sections with a thickness of 4 µm. After retrieval of antigens, quenching of endogenous peroxidase, and blocking of nonspecific binding, the sections were incubated with the corresponding primary antibodies at 4°C. After washing, the tissue sections were incubated with a secondary antibody linked to horseradish peroxidase (Zymed, SanFrancisco, United States). After rinsing in PBS, the sections were stained with DAB (DAKO, Glostrup, Denmark) and counterstained with hematoxylin, and then images were captured by a fluorescence microscope.

### MC3T3-E1 cell culture and treatment

MC3T3-E1 cells were purchased from the Institute of Basic Medical Sciences, Chinese Academy of Medical Sciences, and cultured in alpha-modified minimum essential medium eagle (α-MEM, Shanghai BasalMedia Technologies Co., LTD., China) containing 10% fetal bovine serum (FBS, Gibco, United States) and 1% penicillin-streptomycin (Gibco, United States), incubated at 37°C and 5% CO_2_. The medium was changed every 2 days. MC3T3-E1 was modeled by the method of serum starvation as a control group for subsequent experiments. Firstly different concentrations of BGSSD medicated serum were used to intervene MC3T3-E1 to find the optimal medicated serum concentration. Then the further experiment was performed. The Control, Epidermal growth factor (EGF), and BGSSD groups were treated for 48 h, 72 h, or 7 days with blank serum, EGF (10 ng/ml, Proteintech, United States), and optimal concentration of BGSSD containing serum, respectively.

### Preparation of drug-containing serum

When the BGSSD-Containing Serum was prepared, the intragastric dose of rats was the equivalent dose (5.87 g/kg). After the last intragastric administration, the blood of rats was collected and placed at 4°C for 1 h, then centrifuged for 10 min (4°C, 3000 rpm). The supernatant was collected, and anhydrous ethanol of 4 times the volume of serum was added to precipitate proteins. The suspension was centrifuged for 10 min (4°C, 5000 rpm), and the supernatant was dried in a vacuum centrifuge concentrator (45°C, 1,600 rpm, 300 min). The powder was stored separately at −80°C. To eliminate individual differences, the drug-containing serum of each group was premixed and concentrated. The quality control (QC) of BGSSD medicated serum has been previously examined by high-performance liquid chromatography (HPLC), which ensured the experimental stability and controllability (Supplementary Figure S1).

### Cell counting kit 8 assay

The cell viability was tested via Cell counting kit 8 assay (CCK-8; Bioson, Beijing, China). MC3T3-E1 cells were inoculated into 96-well plates at 5 × 10^3^ per well and incubated overnight to adhere. After 8 h, the cells were treated with different treatments. CCK-8 reagent (10%, v/v) was added after 48 h, 72 h, or 7 days of administration, and the optical density (OD) was detected at 450 nm to analyze the cells’ proliferation.

### Alkaline phosphatase activity assay

MC3T3-E1 cells were seeded into 6-well plates at 5 × 10^4^ cells per well and incubated with a 3 ml medium to adherence. Serum-free medium containing corresponding drugs was added for different groups, 5% CO_2_, 37°C incubating for 72 h. In the end, cells were washed once with PBS, and 100 μL 0.1% Triton X-100 (v/v, PBS) was added, transferring them to 1.5 ml Eppendorf tubes, and the cells were sufficiently lysed by 40% intensity sonication (ice bath operation), centrifuged at 3000 rpm, 4°C for 15 min, collecting the supernatant. BCA protein assay kit (MA, United States) was used to determine the concentration at 3 mg/ml (w/v, PBS). Samples were tested for ALP contents following the enzyme-linked immunosorbent assay (ELISA) kit operating instructions.

### Western blotting

The proteins of animal bone tissue and osteoblasts were quantified using a BCA protein assay kit and separated by electrophoresis in 10% sodium dodecyl sulphate-polyacrylamide gel before being transferred to PVDF membranes (Millipore, United States). PVDF membrane was combined with antibodies of β-actin, ERK1/2 (Proteintech, United States), Smad4 (Proteintech, United States), Runx2 (Bioss, Beijing, China), incubating overnight at 4°C, then shaken with secondary antibody at room temperature for 1 h. Exposure was taken in a TANON gel imager.

### Real-time PCR

Trizol reagent (Solarbio, Beijing, China) was used to extract total RNA from MC3T3-E1 cells and bone tissue of SAMP6. Then TransScript II All-in-One First-Strand cDNA Synthesis SuperMix Kit (Crenscene, China) was used to synthesize the first-strand cDNA templates. According to the manufacturer’s protocol, RT-PCR was carried out by RT-PCR testing equipment (Roche LightCycler^®^ 480II, Switzerland), and the results were analyzed by Applied Biosystems 7500 Fast System SDS software. Using the expression levels of β-actin and calculated by the 2^−ΔΔCt^ method. The primer gene sequences used are listed in Table 1.

**TABLE 1:** Sequences of primers used for RT–PCR.

Gene	Primer sequences (5′→3′)	
ERK1	F:TGTTCCCAAATGCTGACT	R:GGGTCGTAATACTGCTCC
Runx2	F:TCCCTCCATCCTCCCTTATTT	R:CCTCATTCCCTAACCTGAAACC
Smad4	F:ACCTTTACACTCCAACTGC	R:AACTTCCCCAACATTCCT
β-actin	F:ACTGCCGCATCCTCTTCCTC	R:ACTCCTGCTTGCTGATCCACAT

F, forward; R, reverse.

### Statistical analysis

SPSS 24.0 statistical software was used to check the data normality and homogeneity of variance of experimental data. Statistical analysis was performed by one-way ANOVA. All data were presented as mean ± SD. *p* < 0.05 is considered to be statistically significant.

## Results

### Effects of Bu-Gu-Sheng-Sui decoction on femoral bone mass in SAMP6

**FIGURE 1:**
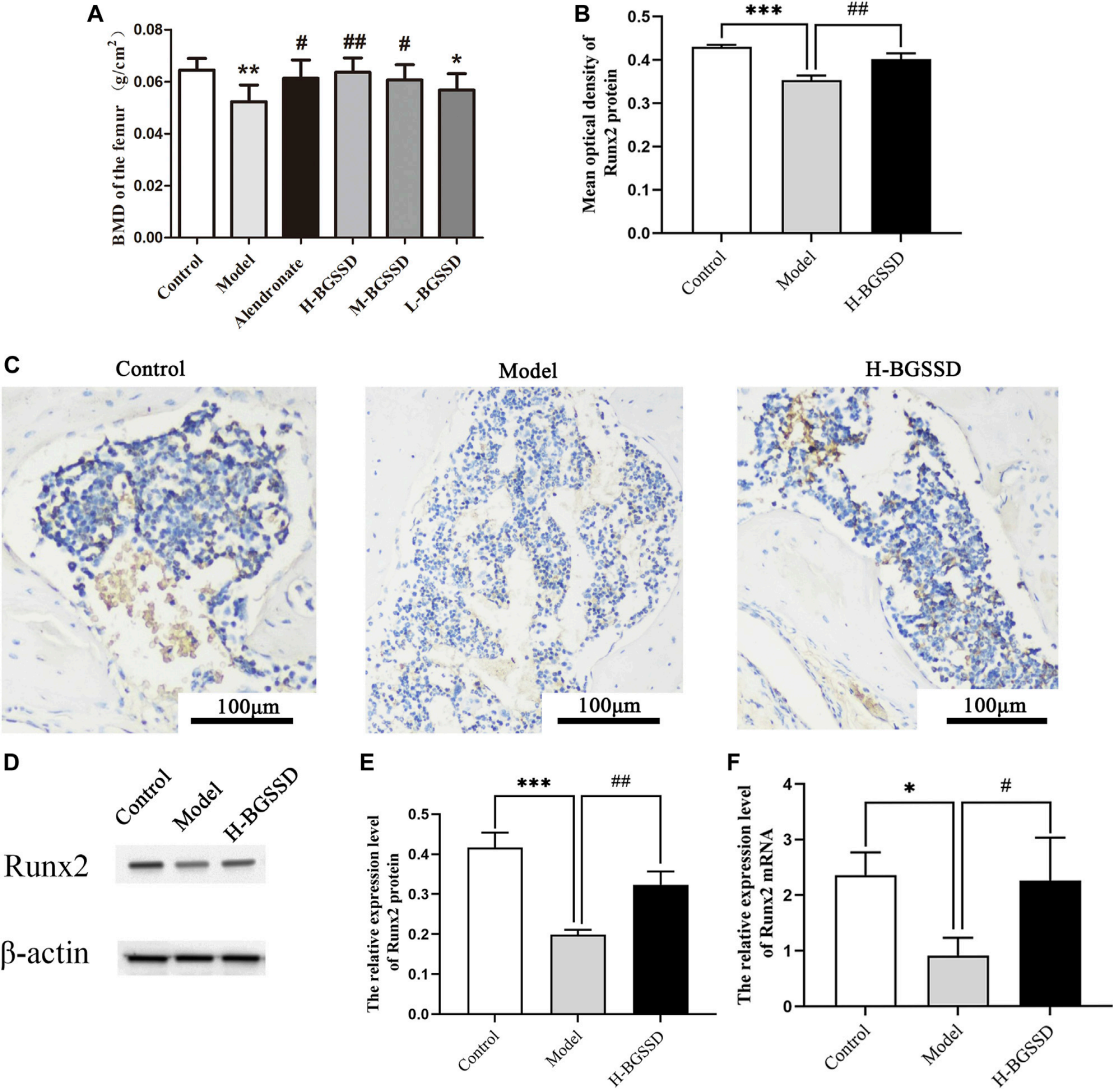
BGSSD promoted bone formation of SAMP6. Control: control group (SAMR1), Model: model group (SAMP6), H-BGSSD: High-dose BGSSD-treated group, M-BGSSD: middle-dose BGSSD-treated group, L-BGSSD: low-dose BGSSD-treated group, Alendronate: positive control, alendronate-treated group. First, BGSSD raised the BMD of the femoral in SAMP6. **(A)** X-ray imaging was performed on the femurs collected from the above groups. BMD of each group was measured by dual-energy X-ray absorptiometry. Data are expressed as mean ± SD, ^*^ *p* < 0.05, ^**^ *p* < 0.01 compared with the control group; ^#^ *p* < 0.05, ^##^ *p* < 0.01 versus the model group. Furthermore, BGSSD promoted overexpression of osteogenic specific factor Runx2 in SAMP6 . **(C)** Immunohistochemical staining was carried out to evaluate the expression of Runx2 protein in bone tissue. Scale bar = 100 µm. **(B)** The mean optical density of Runx2 protein was quantified by ImageJ. Values were expressed as mean ± SD, ^***^ *p* < 0.001 the model group versus the control group; ^##^ *p* < 0.01, the group of H-BGSSD versus the model group. WB was carried out to assess the expression of Runx2 protein in bone tissue. **(D)** Representative images of WB. **(E)** The quantification of the protein expression level of Runx2, samples were collected from the bone tissue. Values were expressed as mean ± SD, ^***^ *p* < 0.001, versus the control group; ^##^ *p* < 0.01, versus the model group. **(F)** The mRNA expression of Runx2 in bone tissue was assessed by RT-PCR. Values were expressed as mean ± SD, ^*^ *p* < 0.05, versus the control group; ^#^ *p* < 0.05, versus the model group. All the assays were repeated more than three times.

**FIGURE 2:**
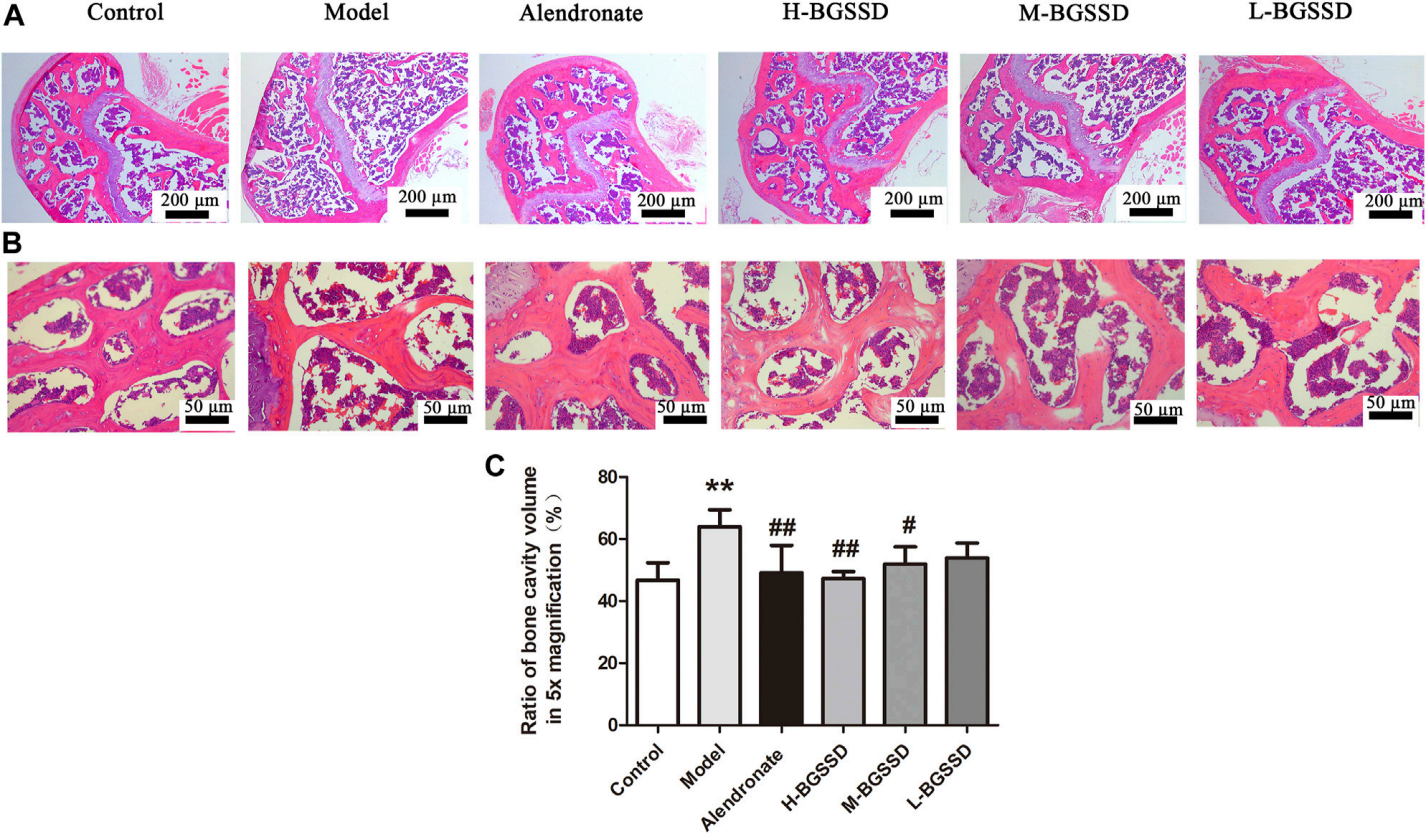
BGSSD ameliorated the bone tissue morphology of SAMP6. The femurs gathered from the above groups were performed by HE staining, the images of trabecular bone structure were selected from blow the growth plates, magnification is ×5 **(A)** and ×20 **(B). (C)** Ratio of bone cavity volume regarding representative HE staining of femoral sections from different groups in ×5 magnification, the site of choice was selected from blow the growth plates. Data are expressed as mean ± SD, ^**^ *p* < 0.01, compared with the control group; ^#^ *p* < 0.05, ^##^ *p* < 0.01, versus the model group. All assays were repeated more than three times.

To identify the pharmacodynamic effects of BGSSD in age-related osteoporosis, we adopted SAMP6, a model of senile osteoporosis, as the subjects, and manifested that the BMD of SAMP6 was significantly lower than SAMR1. It was altered by using BGSSD. The BGSSD-treated groups had higher BMD than the model group, and the H-BGSSD-treated group was the best versus the other two administration groups (Figure 1A; *p* < 0.05). To better compare and analyze the changes in femoral bone mass, we performed HE staining to investigate whether BGSSD affected skeletal histomorphometry in SAMP6. The results demonstrated that the bone trabeculae in the control group were numerous, closely arranged, relatively evenly distributed, and interconnected into a dense network structure. However, the bone trabeculae of SAMP6 were extremely damaged, with few in number, sparse arrangement and interrupted connectivity, and non-reticular structure. Compared with the model group, the trabecular bone structure improved after the intervention of BGSSD. The most apparent performance was the H-BGSSD group (Figures 2A,B); meanwhile, the ratio of bone cavity volume regarding representative HE staining of femoral sections showed that the model group was the largest, improved after BGSSD treatment, and the H-BGSSD group had the best effect (Figure 2C; *p* < 0.05), which were consistent with BMD results. These results indicated that the impact of BGSSD on preventing bone mass loss presented as a dose-dependent trend. H-BGSSD group showed the best therapeutic effect, which was the optimal dosing group for this experiment, so it was selected for IHC, WB, and RT-PCR detection.

Furthermore, Runx2 is a marker for initiating osteoblast differentiation and is the earliest and most specific marker gene during bone formation (Komori, 2020). Meanwhile, Runx2 is the central control gene of the osteoblast phenotype (Franceschi et al., 2009). Therefore, we examined Runx2 expression in bone tissue. Our IHC, WB, and RT-PCR indicated that H-BGSSD increased the expression of osteogenic specific factor Runx2 (Figures 1B–F; *p* < 0.05). Overall, our findings demonstrate that BGSSD treatment could actively protect the bone structure and prevent bone loss.

### Effects of Bu-Gu-Sheng-Sui decoction on the proliferation of osteoblasts

**FIGURE 3:**
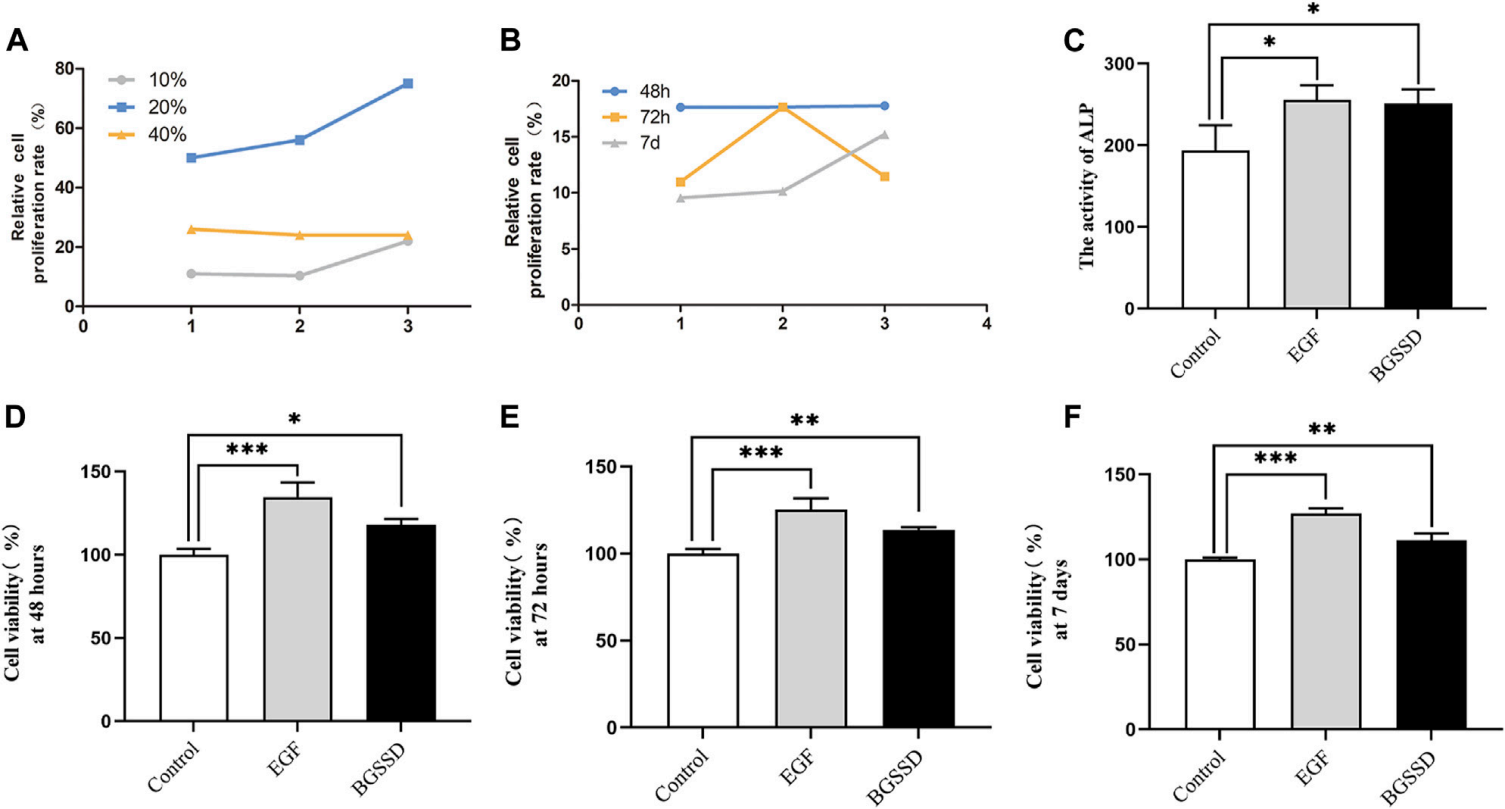
BGSSD increased osteoblast proliferation. Control: MC3T3-E1 + 20% concentration of blank serum; EGF: MC3T3-E1 + EGF; BGSSD: MC3T3-E1 + 20% drug-containing serum of BGSSD. **(A)** The effects of different concentrations of drug-containing serum of BGSSD on MC3T3-E1 proliferation. **(B)** Effect of 20% drug-containing serum of BGSSD on MC3T3-E1 cells proliferation at 48, 72 h and 7 days. **(C)** The activity of ALP was determined by ELISA. **(D–F)** The effect of EGF or 20% drug-containing serum of BGSSD on cell viability at 48, 72 h, and 7 days. The data in the A and B were expressed by a line chart; other data were expressed as mean ± SD. **(C–F)**, ^*^ *p* < 0.05, ^**^ *p* < 0.01, ^***^ *p* < 0.001, versus the control group; all assays were repeated more than three times.

To confirm whether BGSSD affects the proliferation of MC3T3-E1 cells, we used different concentrations of drug-containing serum of BGSSD to treat MC3T3-E1 cells. The CCK-8 assay was applied for determination. The results showed that different concentrations of drug-containing serum of BGSSD promoted osteoblasts proliferation, and the 20% drug-containing serum of the BGSSD group performed the best effect (Figure 3A). Then, we used EGF or 20% drug-containing serum of BGSSD to intervene in MC3T3-E1 cells. The consequences revealed that EGF and 20% drug-containing serum of BGSSD elevated the viability of MC3T3-E1 cells after 48 h, 72 h, and 7 days, compared with untreated cells (Figures 3D–F; *p* < 0.05). Furthermore, after being treated with 20% drug-containing serum of BGSSD, the differentiation of MC3T3-E1 cells had the most significant effect at the 48-h time point (Figure 3B). Therefore, the cells treated for 48 h were selected as the samples for further detection. Altogether, our data suggest that BGSSD could promote the proliferation of osteoblasts.

### Bu-Gu-Sheng-Sui decoction upregulated osteoblast alkaline phosphatase activity

To determine whether BGSSD increases bone mass by affecting the process of osteoblastogenesis, we applied 20% drug-containing serum of BGSSD and EGF to intervene MC3T3-E1 *in vitro* and measured the activity of ALP. The results demonstrated that the levels of ALP enhanced after intervention in both the EGF group and the BGSSD group versus the control group (Figure 3C; *p* < 0.05). Since ALP starts to be secreted during the phase of bone matrix synthesis, which can stimulate processes such as inorganic phosphorus release and matrix mineralization (Sunden et al., 2017), the elevation of ALP is currently the most widely recognized marker of osteoblast differentiation (Lee et al., 2011). All these demonstrated that the activity of ALP affects the differentiation of osteoblasts. Thus, our findings prove that BGSSD may stimulate osteoblast differentiation by upregulating ALP activity in MC3T3-E1 cells.

### Bu-Gu-Sheng-Sui decoction promoted the expression of osteogenic specific factor Runx2

Runx2 is essential for regulating the expression of genes responsible for osteogenic specific matrix proteins (including ALP, Col I, BSP, OCN, and OPN), and it has a significant effect on the development, proliferation, differentiation, and mineralization of osteoblasts (Yang et al., 2011). To observe the effect of BGSSD-containing serum on osteoblast, we examined Runx2 expression in osteoblasts. The vitro experiments results showed that drug-containing serum of BGSSD and EGF increased the protein and mRNA levels of Runx2 compared with the control (Figures 5D,G, 6E; *p* < 0.05). These results suggested that BGSSD may promote osteoblast proliferation, differentiation, and mineralization.

### Bu-Gu-Sheng-Sui decoction regulated osteoblast differentiation by activating ERK/Smad

**FIGURE 4:**
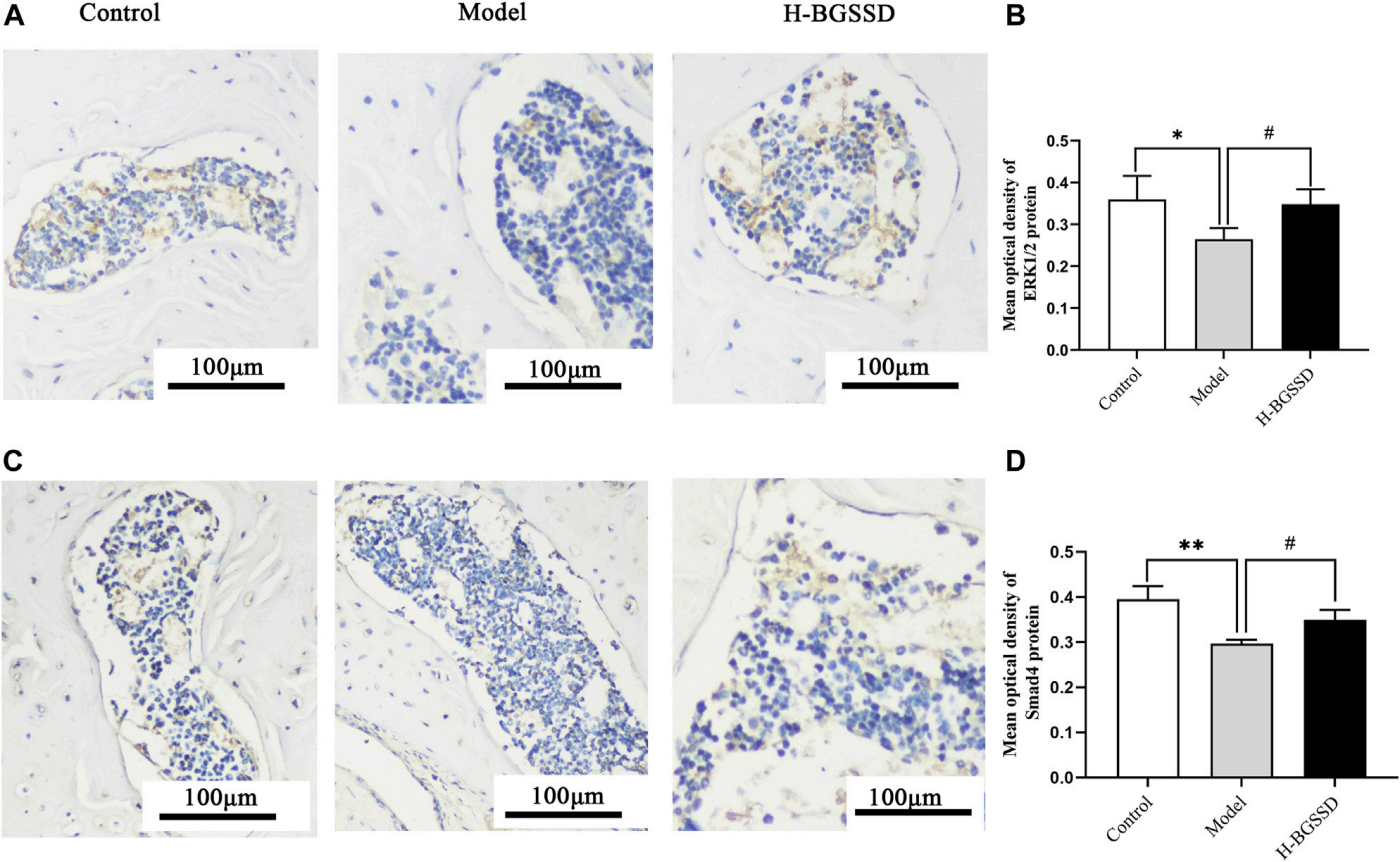
BGSSD enhanced the expression of ERK1/2 and Smad4 proteins of SAMP6. **(A,C)** Immunohistochemical staining was carried out to evaluate the expression of ERK1/2 and Smad4 protein. Scale bar = 100 µm. **(B,D)** The mean optical density of ERK1/2, Smad4 protein was quantified by ImageJ. Values were expressed as mean ± SD, ^*^ *p* < 0.05, ^**^ *p* < 0.01, the model group versus the control group; ^#^ *p* < 0.05, the group of high-dose BGSSD versus the model group. All the assays were repeated more than three times.

**FIGURE 5:**
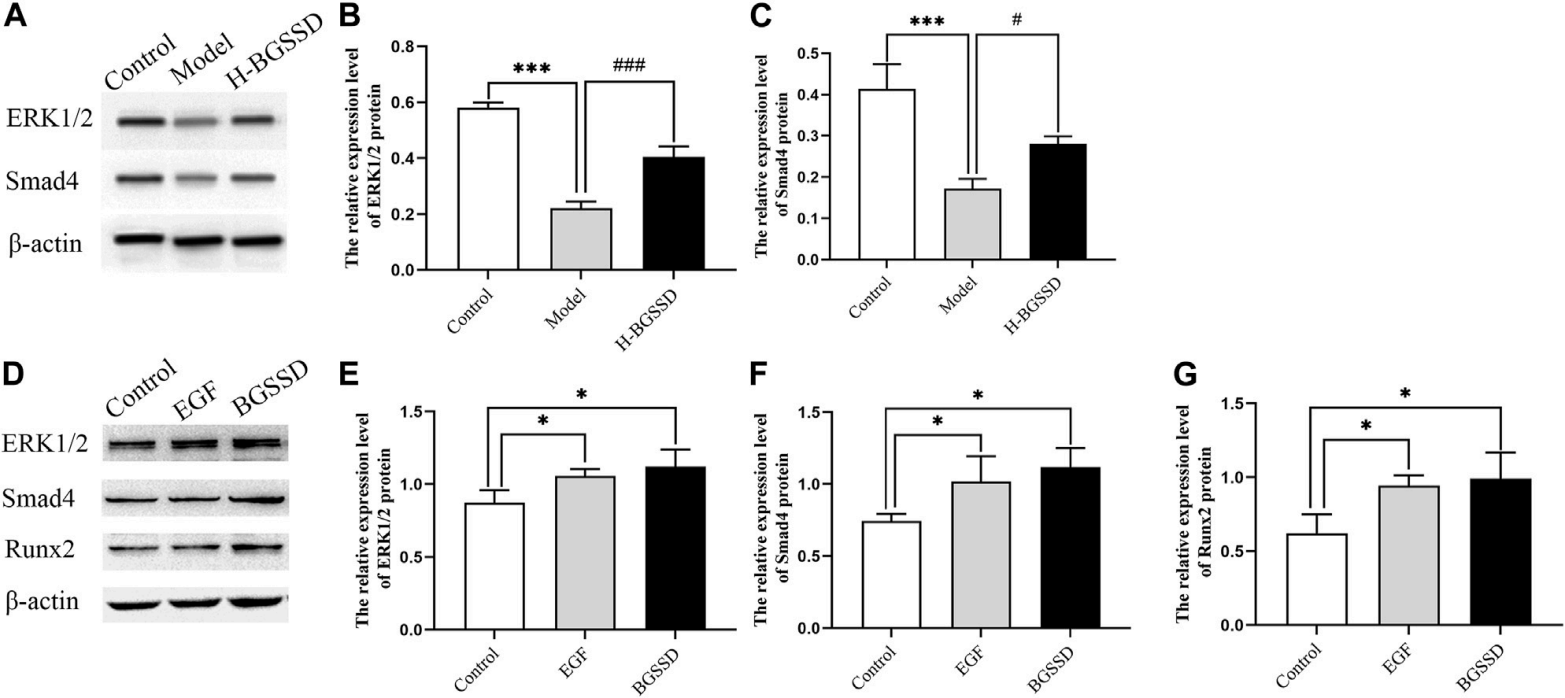
*In vivo*, BGSSD induced overexpression of ERK1/2, Smad4 protein of SAMP6. **(A)** Representative images of WB. **(B,C)** The quantification of the protein expression level of ERK1/2, Smad4, samples were collected from the bone tissue. Values were expressed as mean ± SD, ^***^ *p* < 0.001, versus the control group; ^#^ *p* < 0.05, ^###^ *p* < 0.001, versus the model group. *In vitro*, BGSSD medicated serum enhanced ERK1/2, Smad4, and Runx2 protein expression in MC3T3-E1 cells. **(D)** The WB bands of the cell experiment. **(E–G)** the quantification of the protein expression level of ERK1/2, Smad4, and Runx2 in MC3T3-E1 cells before and after 20% BGSSD medicated serum treatment. Values were expressed as mean ± SD, ^*^ *p* < 0.05, compared with the control group. Furthermore, the agonist of ERK, EGF, activated the ERK1/2 protein, meanwhile, stimulated the expression of Smad4 and Runx2 protein in MC3T3-E1 cells. **(D)** Representative images of WB. **(E–G)** The quantification of the protein expression level of ERK1/2, Smad4, Runx2 after EGF invention. Values were expressed as mean ± SD, ^*^ *p* < 0.05, versus the control group. All the assays were repeated more than three times.

**FIGURE 6:**
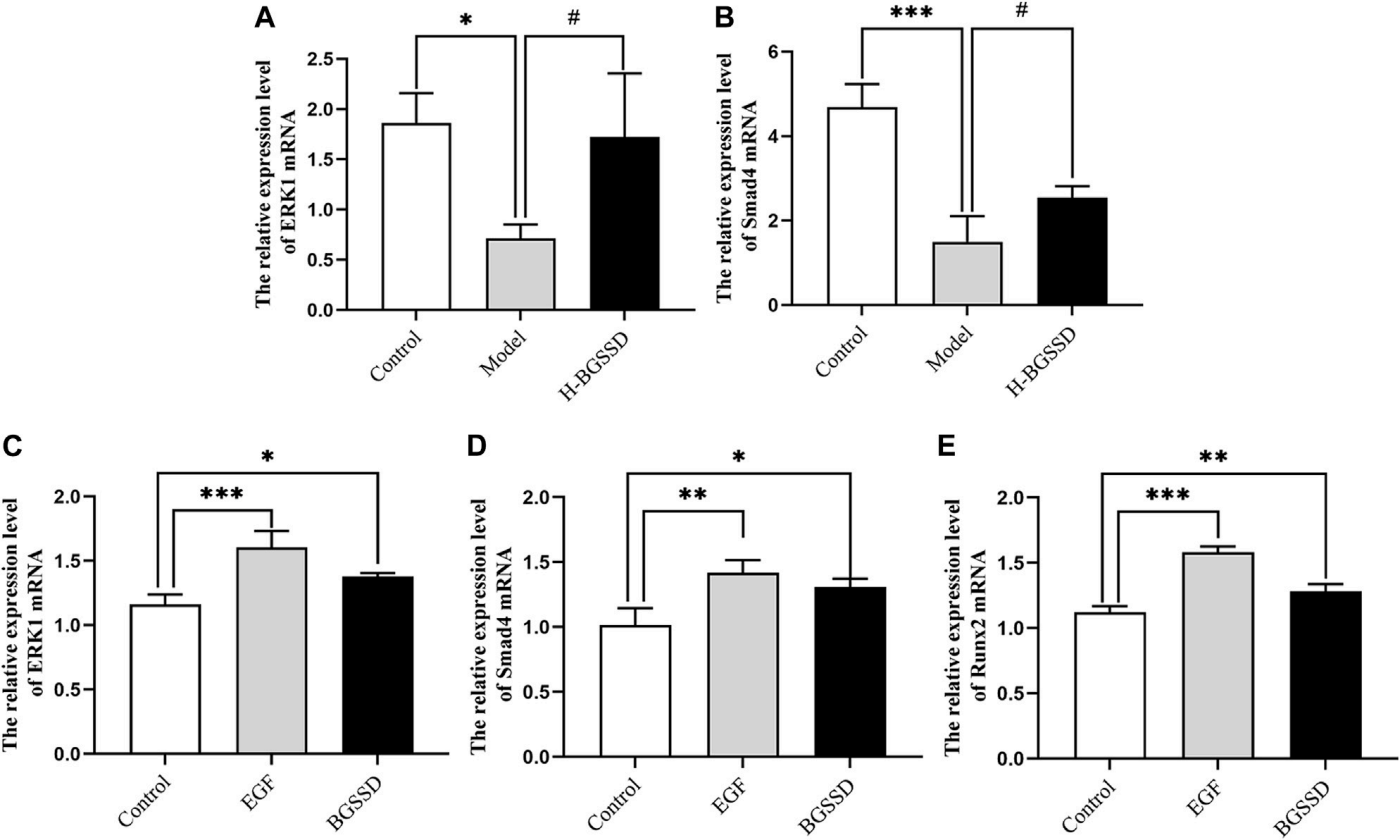
BGSSD stimulated the expression of ERK1 and Smad4 mRNA *in vivo*. **(A,B)** The mRNA expression of ERK1 and Smad4 in SAMP6 were assessed by RT-PCR. Values were expressed as mean ± SD, ^*^ *p* < 0.05, ^***^ *p* < 0.001, versus the control group; ^#^ *p* < 0.05, versus the model group. And BGSSD medicated serum enhanced expression of ERK1, Smad4, and Runx2 mRNA *in vitro*. **(C–E)** The quantification of ERK1, Smad4, and Runx2 mRNA in MC3T3-E1 cells before and after 20% BGSSD medicated serum treatment. Values were expressed as mean ± SD, ^*^ *p* < 0.05, ^**^ *p* < 0.01, compared with control group. **(C–E)** The expression of ERK1, Smad4, and Runx2 mRNA after EGF intervention. Values were expressed as mean ± SD, ^**^ *p* < 0.01, ^***^ *p* < 0.001, compared with the control group. All the assays were repeated more than three times.

To investigate the mechanisms involved in regulating BGSSD-induced osteoblast proliferation, we assayed the expressions of ERK and Smad *in vivo* and *in vitro*. *In vivo*, IHC and WB were performed to assess the protein levels of ERK1/2 and Smad4. IHC staining demonstrated that the levels of ERK1/2 and Smad4 were lower in the model group than those in the control group; after H-BGSSD treatment, the expression of these proteins significantly increased compared with the model group (Figures 4A,C). The mean optical density of ERK1/2 and Smad4 also illustrated this point (Figures 4B,D; *p* < 0.05). This observation was subsequently confirmed in the results of WB bands (Figure 5A) and the relative expression levels of proteins (Figures 5B,C; *p* < 0.05). *In vitro*, 20% drug-containing serum of BGSSD promoted the expression of ERK1/2 and Smad4 protein compared with the control (Figures 5D–F; *p* < 0.05). Moreover, RT-PCR was also performed. *In vivo*, RT-PCR manifested that the mRNA expression levels of ERK1 and Smad4 in the model group were lower versus the control group, which were reversed by H-BGSSD, and the expression of ERK1 and Smad4 mRNA significantly increased (Figures 6A,B; *p* < 0.05). *In vitro*, 20% drug-containing serum of BGSSD promoted the expression of ERK1 and Smad4 mRNA versus the control group (Figures 6C,D; *p* < 0.05). Thus, our results indicated that BGSSD could boost the expression levels of ERK and Smad.

To further verify the existence of crosstalk between the ERK and Smad pathways, we overexpressed ERK by using EGF, an agonist of ERK. WB and RT-PCR were performed to detect the expression of ERK, Smad-related proteins, and mRNA. EGF overexpressed the expression of ERK1/2 protein (Figures 5D,E; *p* < 0.05) and, meanwhile, increased the protein level of Smad4 (Figures 5D,F; *p* < 0.05) compared with the control group. Moreover, the result of RT-PCR indicated that EGF stimulated the overexpression of ERK1 (Figure 6C; *p* < 0.05), at the same time, raised the level of Smad4 mRNA (Figure 6D; *p* < 0.05), versus the control group. Thus, we demonstrated that there was crosstalk between ERK and Smad signaling pathways. From all these results, we conclude that BGSSD may activate the ERK/Smad signal transduction pathway to promote bone formation.

## Discussion

We have previously confirmed that the BGSSD is influential in the treatment of OP clinically, and it enhances the BMD of the lumbar spine and femur in ovariectomized rats and improves bone biomechanical indexes. However, its molecular mechanism for osteogenesis is still unclear. In the present study, we discovered that BGSSD could accelerate bone formation in SAMP6 and positively affect MC3T3-E1. At the same time, we investigated the underlying mechanism of BGSSD, and our study demonstrated that BGSSD could promote osteoblast proliferation and osteogenesis by regulating the ERK/Smad pathway.

In the animal experiments, we chose SAMP6 as the model. SAMP6 was developed by Japanese scholars from AKR/J strains is a valuable mouse model of senile osteoporosis. They are characterized by peak bone mass at 4 months, but their peak bone mass is lower than SAMR1 (Chawalitpong et al., 2018). The osteoblastic hypoplasia of SAMP6 leads to degradation in the number of bone trabeculae, thinning of cortical bone, and decrease in bone formation, and eventually decline in bone density, bone calcium, and bone phosphorus (Oda et al., 2018). These features are similar to the changes in the bones of the elderly (Kasai et al., 2004) and show the characteristics of low turnover rate osteoporosis, so it is widely used in animal models of senile osteoporosis (Antika et al., 2017; Kim et al., 2019). To verify the pharmacodynamic effect of BGSSD on SAMP6, we evaluated the bone density and bone tissue morphology of SAMP6. Our results showed that BGSSD ameliorated the morphology and microstructure of trabecular bone in SAMP6, raised bone mass, and elevated bone density. Thus, we confirm that BGSSD can stimulate bone formation.

In cellular models, we selected MC3T3-E1. MC3T3-E1 cells are osteoblastic precursor cells that can be further differentiated into mature osteoblasts under the induction and stimulation of osteogenic signals (Long, 2011). It has osteoblast characteristics, strong proliferation ability, and stable cell biology. So, it is a good osteoblast differentiation research model (Chaves Neto et al., 2011). Currently, the most widely recognized biochemical marker of osteoblast differentiation is increasable in ALP activity, and the level of ALP activity reflects the degree of osteogenic differentiation (Lee et al., 2011). Studies showed that kidney-tonifying herbs could upregulate ALP activity and osteogenic differentiation of MC3T3-E1 cells (Zhang et al., 2016). Ge et al. (2018) demonstrated, that is, opsoralen promoted the ossification of MC3T3-E1 cells and the activity of ALP. In Li et al. (2016) study, Bu Shen Huo Xue Gu Chi decoction promoted the development of MC3T3-E1 cells to osteoblast-like phenotype, increased the ALP activity of MC3T3-E1 cells, accelerated the differentiation and maturation of MC3T3-E1 cells, and stimulated the formation of bone tissue. We checked the ALP activity in MC3T3-E1 cells and tested the effect of BGSSD-containing serum on the differentiation of MC3T3-E1 cells. We discovered that BGSSD increased ALP’s activity and promoted osteoblasts’ proliferation. Therefore, our findings prove that BGSSD can promote osteoblast differentiation.

Runx2 is the earliest and most specific marker gene in bone formation (Komori, 2020). It is essential for regulating the expression of genes responsible for osteogenic specific matrix proteins, and it significantly affects the development, proliferation, differentiation, and mineralization of osteoblasts (Yang et al., 2011; Komori, 2019). Deleting runt in osteoblasts results in no bone phenotype, lower transcription activation capacity, and bone loss (Adhami et al., 2014). Thus, Runx2 plays a crucial part in osteoblast differentiation and osteogenesis (Li et al., 2017; Kim et al., 2020). Studies confirmed that the recipe of Osteoking stimulated the proliferation and differentiation of MC3T3-E1 by regulating the expression of bone-specific factor Runx2 (Qin et al., 2019). Sun et al. (2021a) proved that the total flavonoids of Rhizoma Drynariae upregulated the expression of Runx2 and accelerated bone formation in rats. We examined Runx2 protein and mRNA expression in SAMP6 and MC3T3-E1 cells. The results showed that the expression of Runx2 was enhanced. These data reveal that BGSSD may induce bone formation by promoting osteoblast differentiation and mineralization.

Based on these, we explored the potential molecular mechanism of BGSSD in promoting osteogenesis. As we all know, there are many mechanisms for the regulation of bone remodeling. ERK and Smad played momentous roles in the modulation of osteoblasts and could participate in the proliferation and differentiation of osteoblasts (Lee et al., 2018). According to reports, the activation of the ERK signaling pathway was crucial to the proliferation and differentiation of osteoblasts, and it can upregulate the expression of bone formation-related genes, promote the differentiation and mineralization of osteoblasts, and play an essential role in bone formation and bone homeostasis (Miao et al., 2018; Sun et al., 2018; Zhang et al., 2019). Liu et al. (2018) reported that lactoferrin modulated the ERK signaling pathway to stimulate osteogenesis *in vivo*; Wang et al. (2019) revealed that Melatonin can alleviate bone loss in retinoic acid-induced OP model mice, repair trabecular bone microstructure, and promote bone formation by regulating the ERK/Smad pathway. Vitro experiments revealed that the proliferation of osteoblasts and primary osteoblasts was enhanced under the action of ERK protein (Peng et al., 2018; Assefa et al., 2022). Studies found that Longan fruit extract upregulated ALP activity of MC3T3-E1 cells, induced mineralization, and promoted Runx2 expression. In addition, it activated the ERK1/2 pathway (Park et al., 2016); Wang et al. (2017) indicated that Naringin could significantly promote the proliferation and osteogenic differentiation of bone marrow mesenchymal stem cells and increase the protein and mRNA expression levels in a dose-dependent manner, such as Runx-2, OCN. They revealed that Naringin enhanced osteogenic differentiation was related to the activation of ERK. Moreover, SPRY as a receptor tyrosine kinase (RTK)-associated signaling protein reduced osteogenic differentiation and bone formation when the ERK signaling pathway was restrained (Park et al., 2019). As well the Smad pathway also plays a significant role in bone formation. The expression of Smad is closely related to the number of osteoblasts, bone formation, and BMD (Jin et al., 2020). It was found that *Prunella* vulgaris could relieve glucocorticoid-induced osteogenesis inhibition by activating the Smad pathway and prevented the deterioration of OP (Xi et al., 2020), which was also supported by Cui et al. (2019) result. Our study verified that BGSSD upregulated the protein and mRNA levels of ERK and Smad in SAMP6 and MC3T3-E1 cells. We conclude that the molecular mechanism of BGSSD seems to depend partly on ERK and Smad signaling pathways.

The crosstalk between ERK and Smad signaling was described more than a decade ago (Zhou et al., 2007; Park et al., 2011), and recent studies showed (Park et al., 2016; Liu and Yang, 2020) that the ERK activity increased Smad-mediated transcription. Others indicated that ERK inhibitors could down-regulate the expression of Smad-related proteins and affected bone formation (Mei et al., 2013). In our study, we used the EGF to promote the expression of ERK and found that Smad’s expression also increased, agreeing with the results in BGSSD. Therefore, we deduce that the BGSSD may activate ERK and Smad to upregulate osteogenesis, and the two signal pathways communicate through signal crosstalk.

However, some limitations of this study should be noted. Firstly, we detected the trabecular bone morphology and structure by HE. Although it indicated that BGSSD could improve the bone trabecular structure and promote bone formation, it is obviously not as intuitive and accurate as Micro-CT for observing bone microstructure. Secondly, we have not verified the exact mechanism of BGSSD through inhibitor or RNAi, which we will further investigate.

## Conclusion

Taken together, BGSSD has a positive effect on osteoblast differentiation and bone formation. Such responsiveness may predominantly be associated with ERK/Smad signaling pathways. Therefore, this study provides experimental evidence of BGSSD as a treatment for osteoporosis, and it suggests that BGSSD is an effective drug for the prevention and treatment of osteoporosis.

## Data Availability

The original contributions presented in the study are included in the article/Supplementary Materials, further inquiries can be directed to the corresponding author.
