# Supplementary figures and images for: Bu-Gu-Sheng-Sui decoction promotes osteogenesis via activating the ERK/Smad signaling pathways
Source: Front Pharmacol. 2022 Aug 25;13:976121. doi: 10.3389/fphar.2022.976121 (PMC9453880; doi:10.3389/fphar.2022.976121)

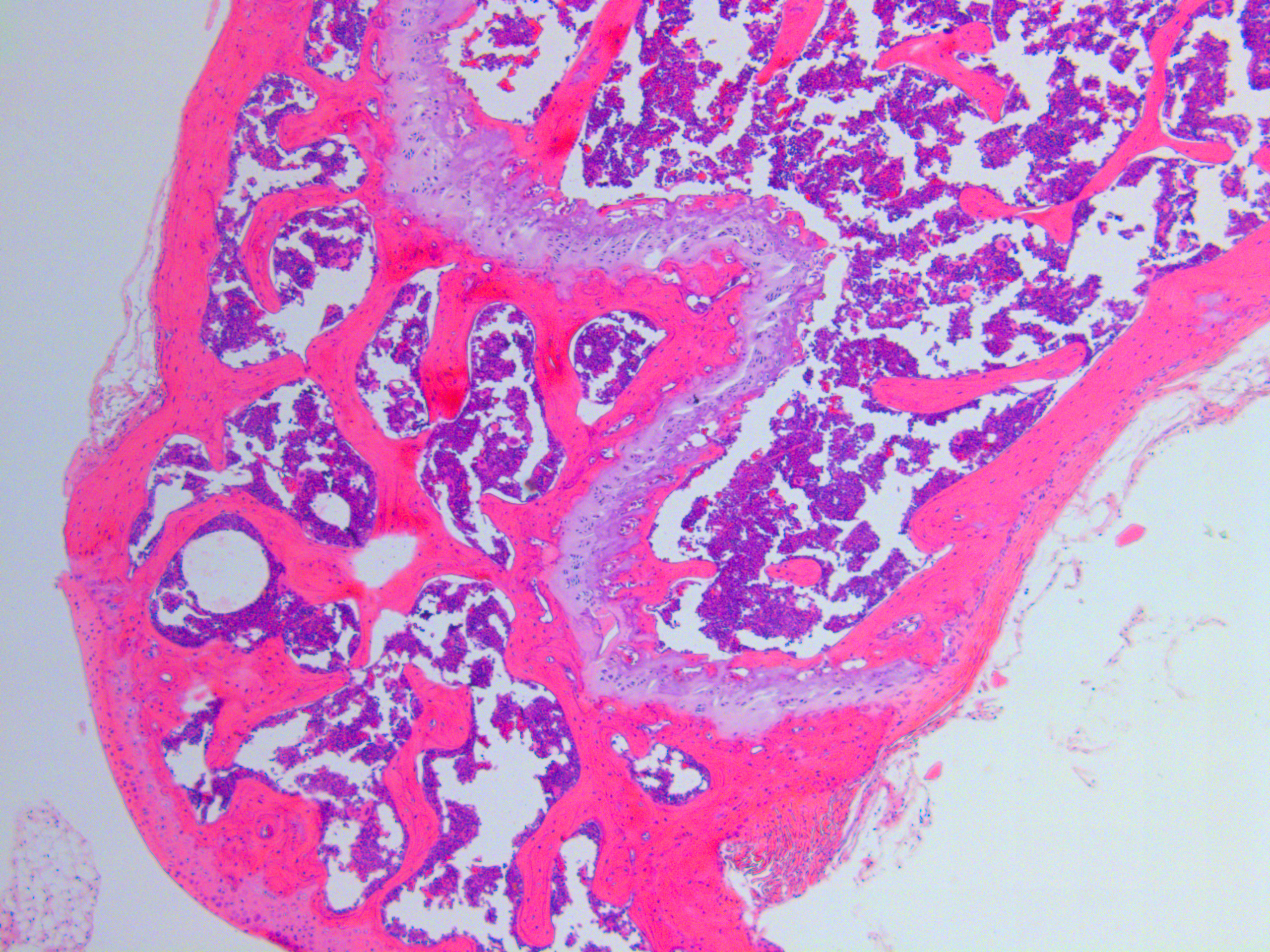

Supplement: Supplementary file 1 [file DataSheet3.zip › Figure2A-HE×5(The first sample from each group) - 副本/H-BGSSD.tif]

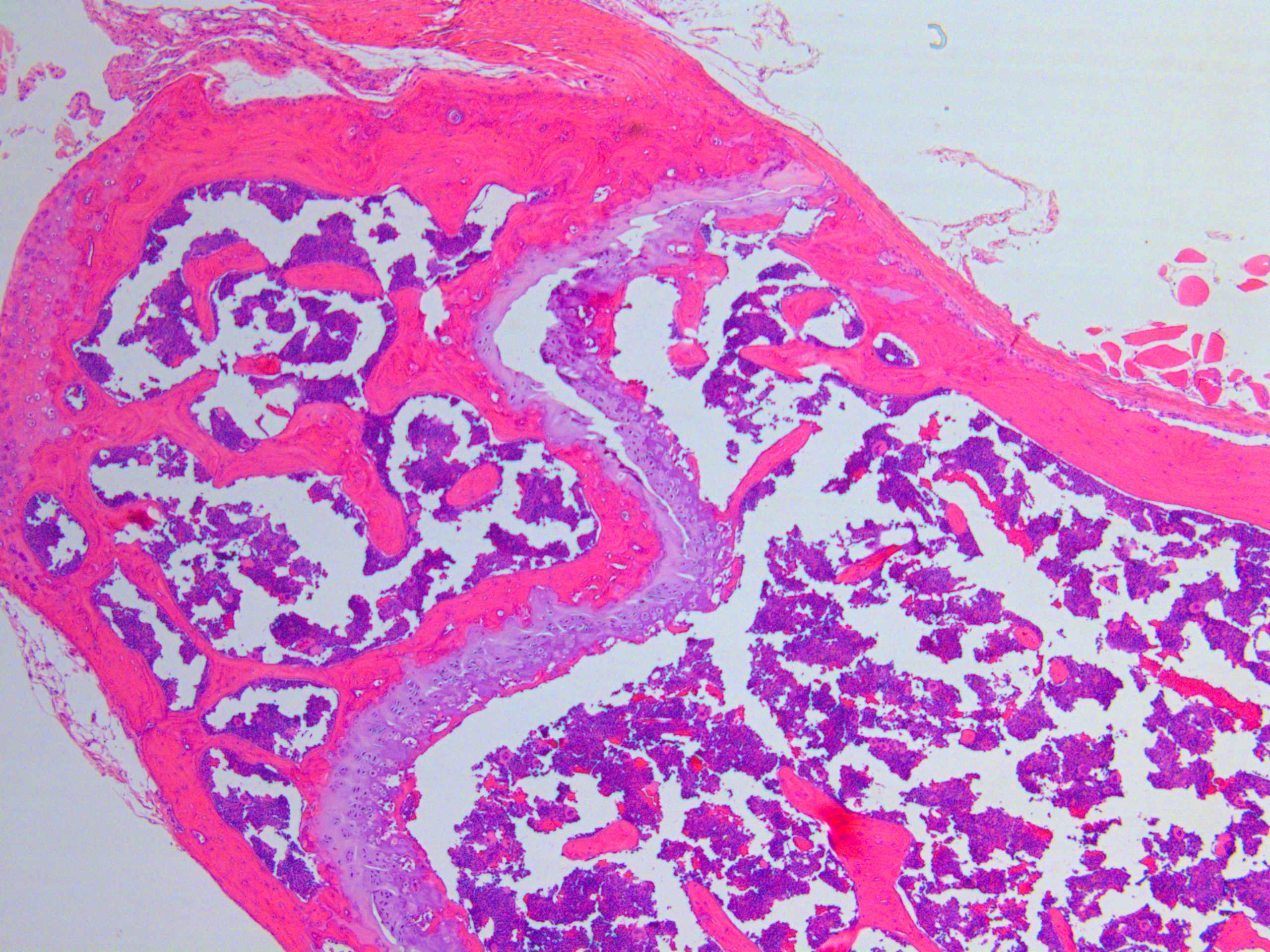

Supplement: Supplementary file 1 [file DataSheet3.zip › Figure2A-HE×5(The first sample from each group) - 副本/L-BGSSD.tif]

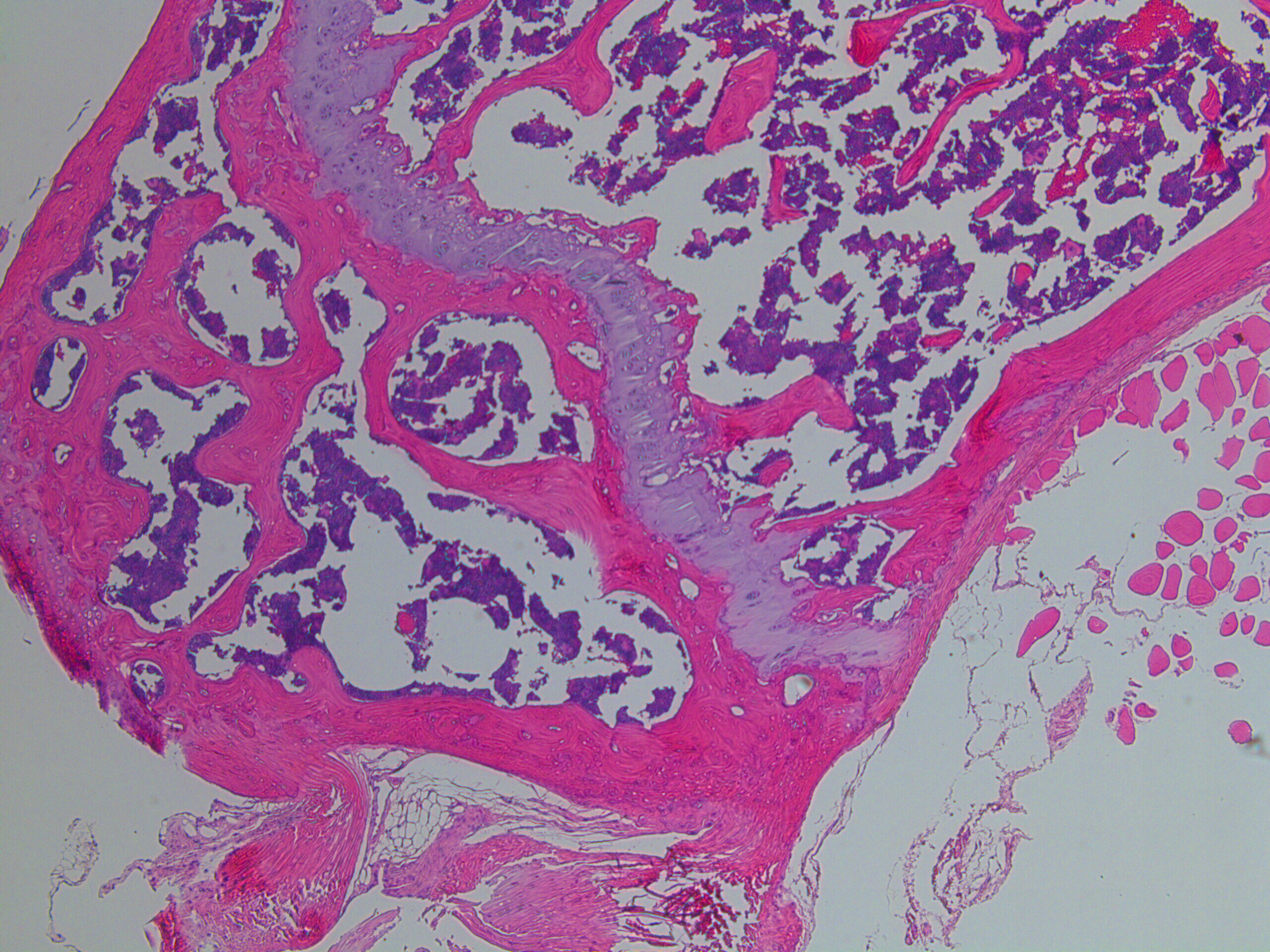

Supplement: Supplementary file 1 [file DataSheet3.zip › Figure2A-HE×5(The first sample from each group) - 副本/M-BGSSD.jpg]

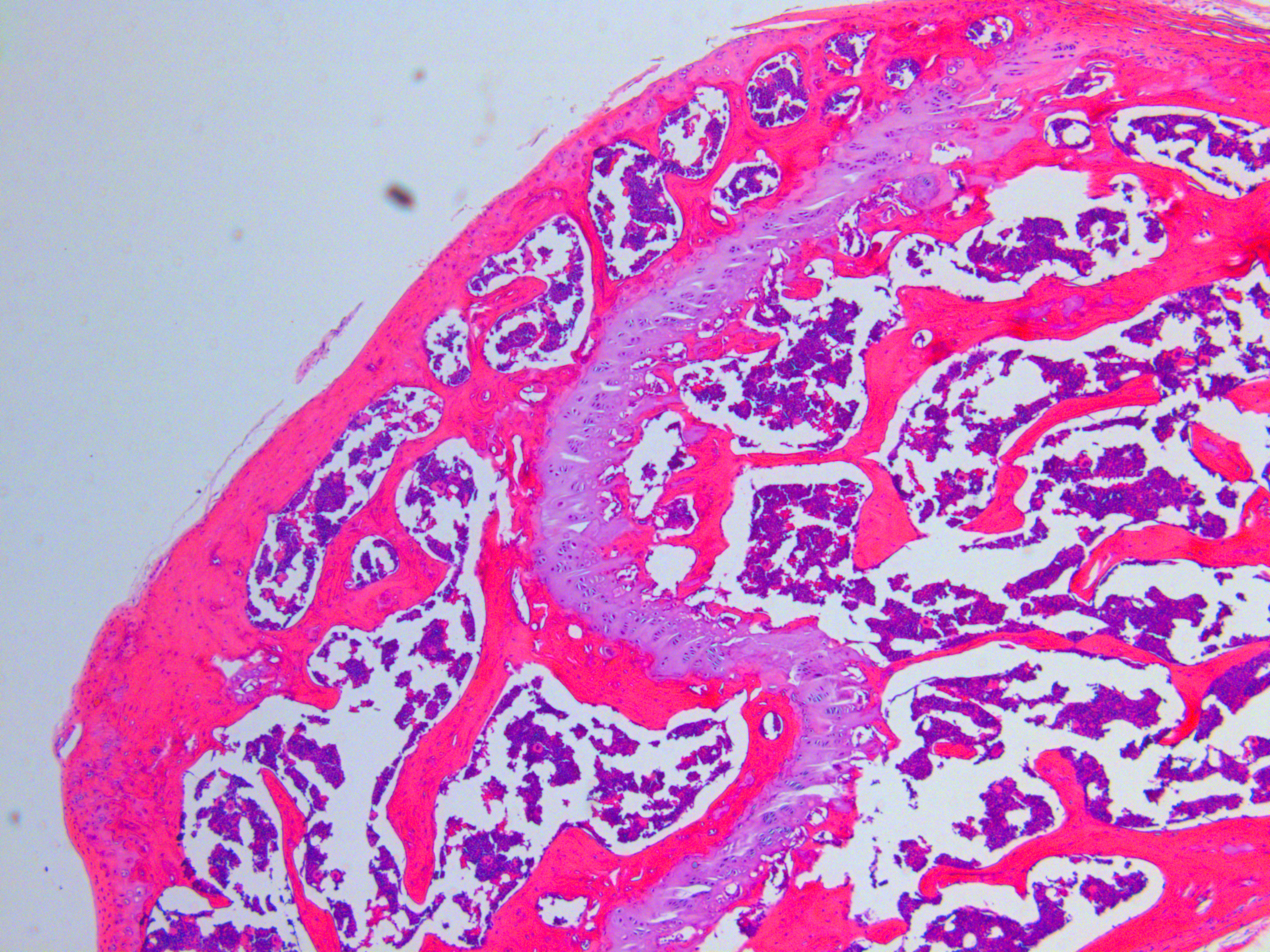

Supplement: Supplementary file 3 [file DataSheet11.zip › HE×5(The third sample from each group) - 副本/Alendronate.tif]

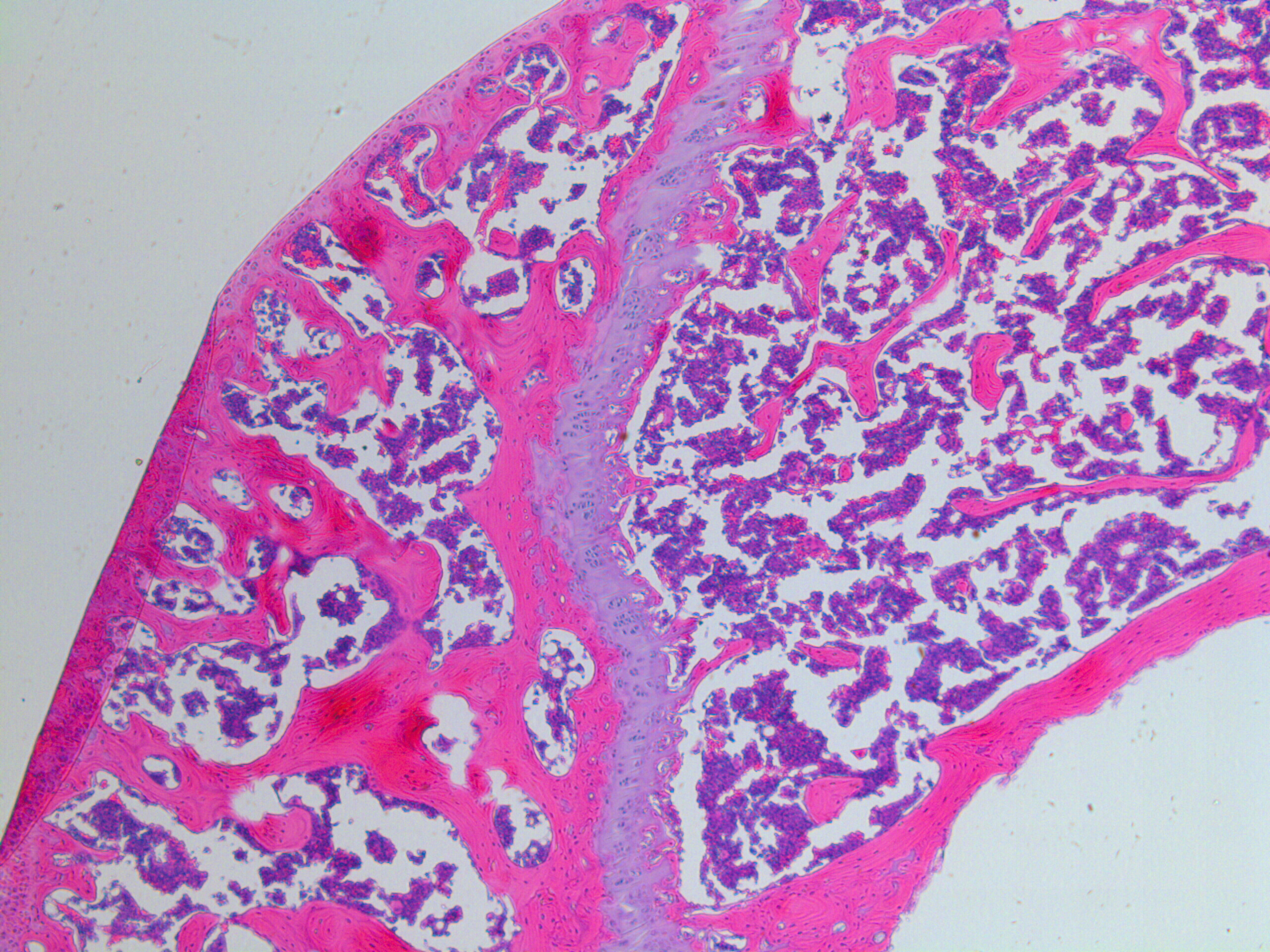

Supplement: Supplementary file 3 [file DataSheet11.zip › HE×5(The third sample from each group) - 副本/Control.jpg]

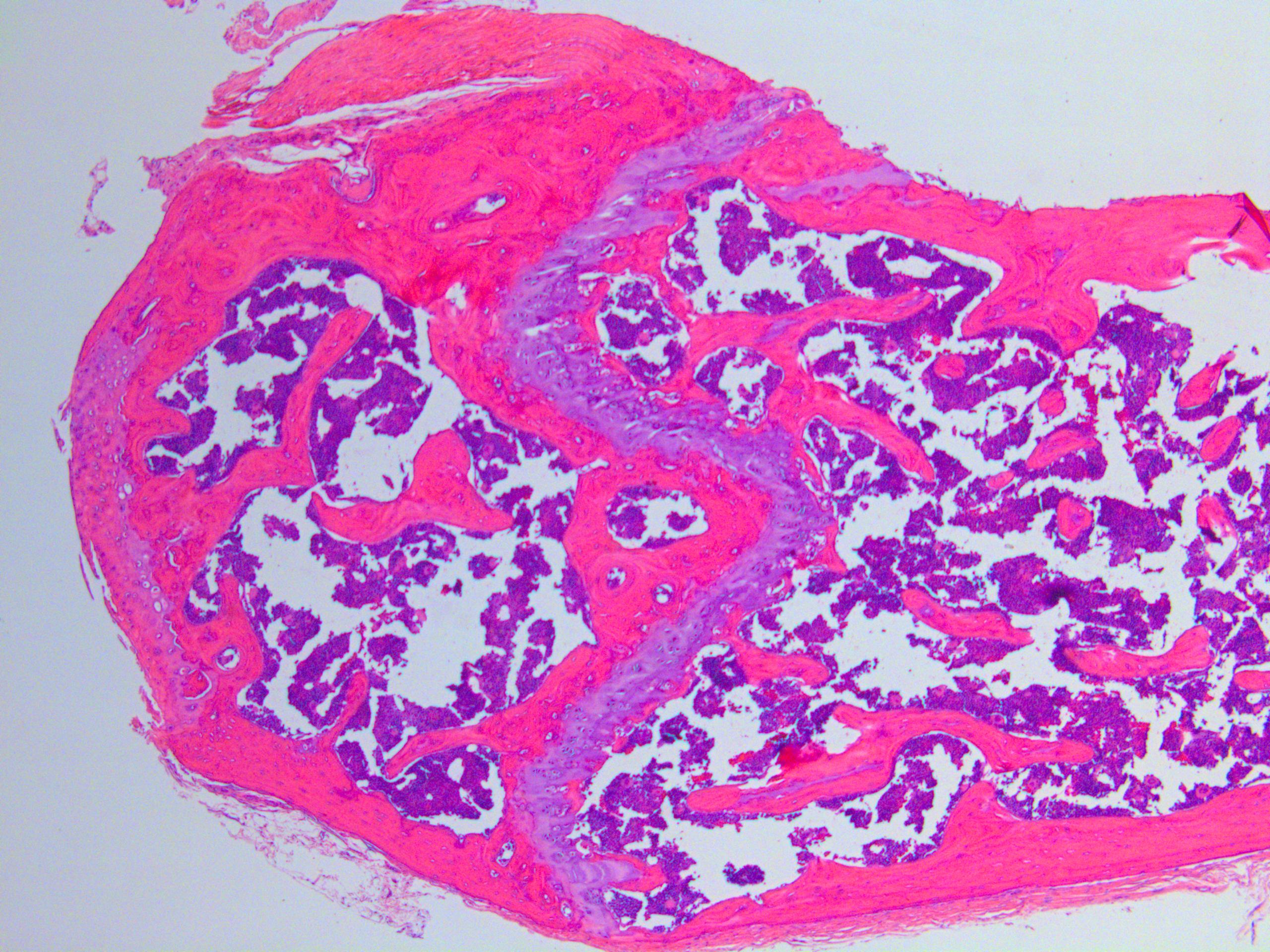

Supplement: Supplementary file 3 [file DataSheet11.zip › HE×5(The third sample from each group) - 副本/Model.tif]

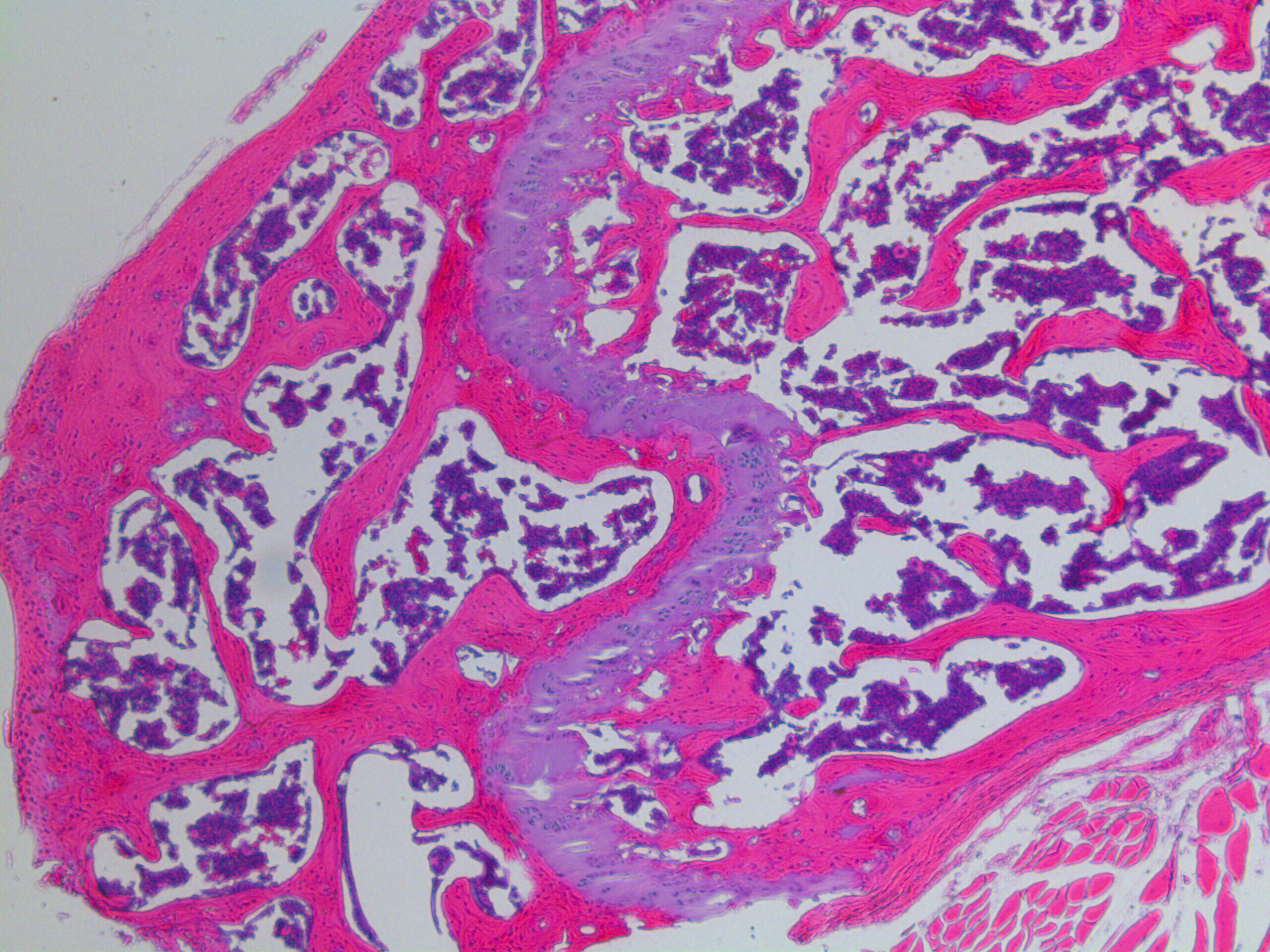

Supplement: Supplementary file 4 [file DataSheet8.zip › HE×5(The second sample from each group)/Alendronate.jpg]

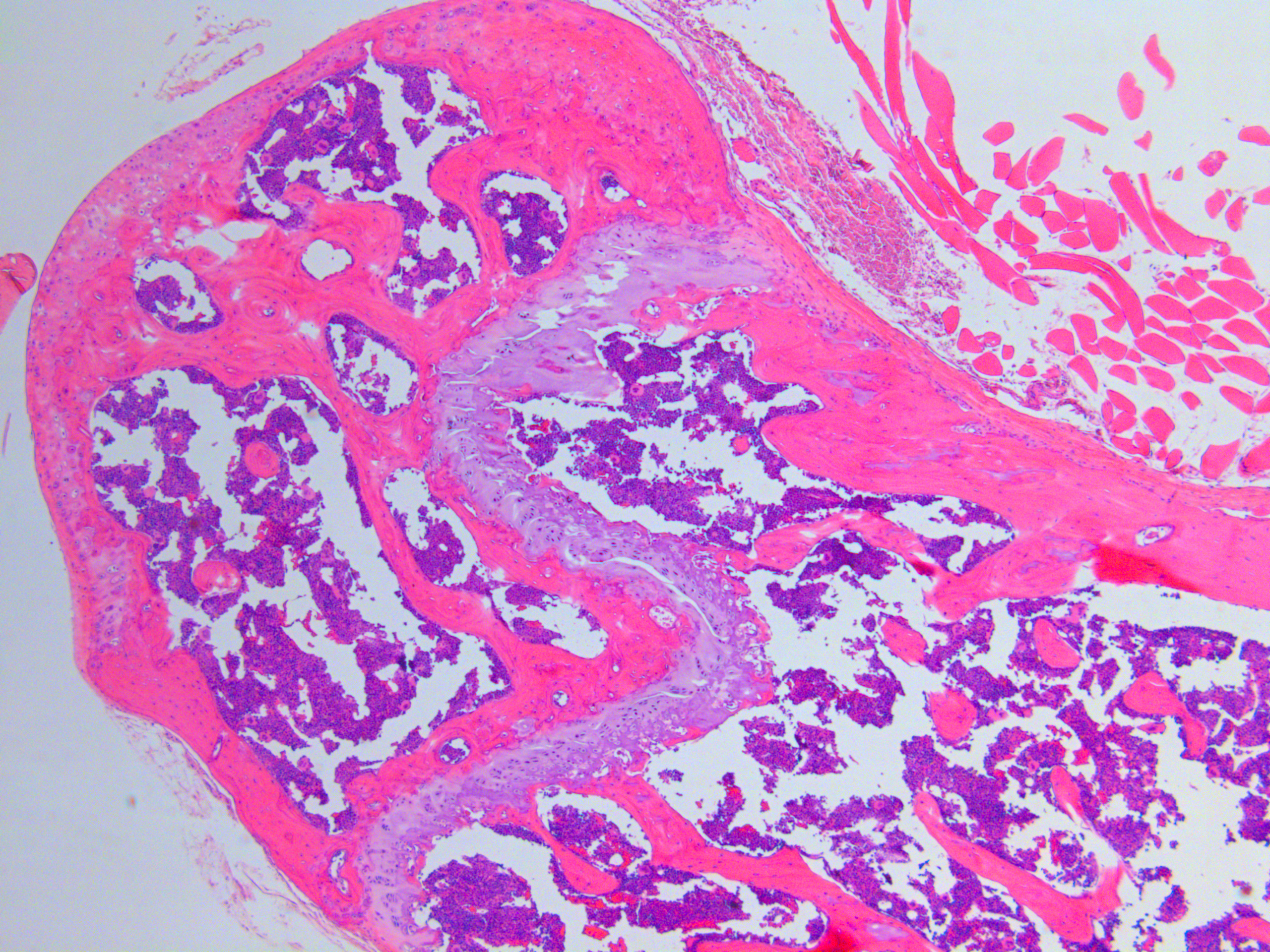

Supplement: Supplementary file 4 [file DataSheet8.zip › HE×5(The second sample from each group)/L-BGSSD.tif]

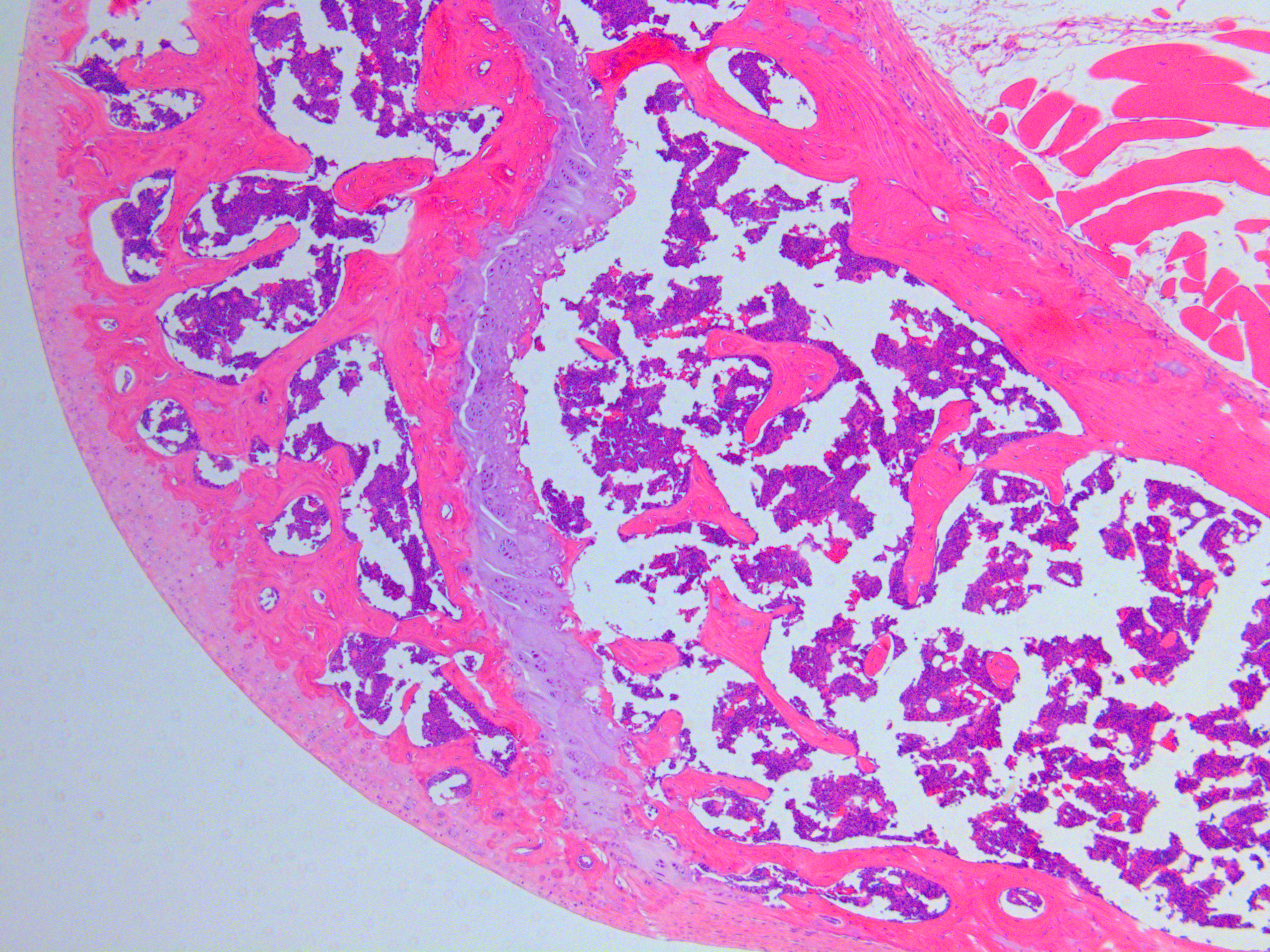

Supplement: Supplementary file 4 [file DataSheet8.zip › HE×5(The second sample from each group)/M-BGSSD.tif]

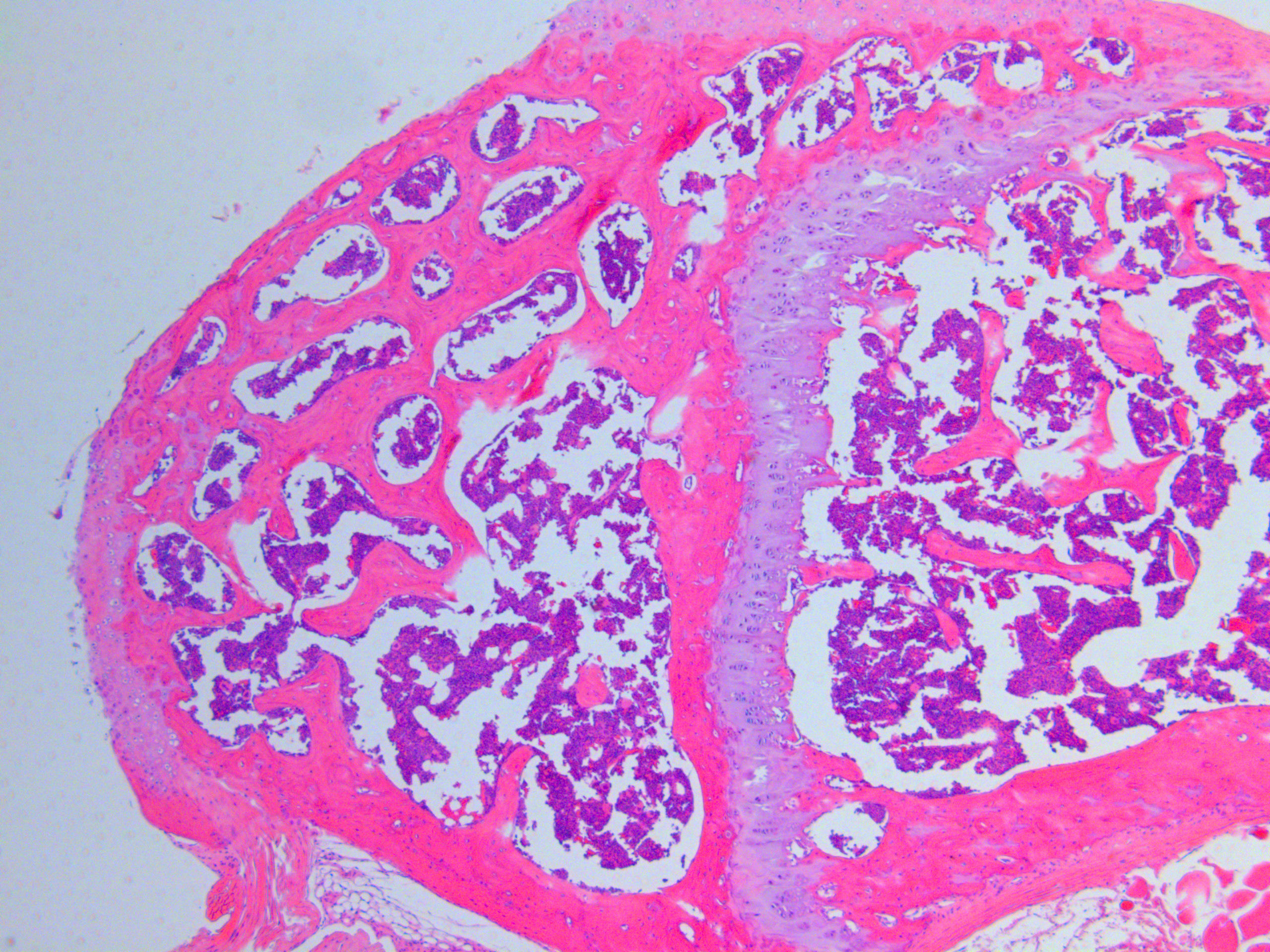

Supplement: Supplementary file 5 [file DataSheet9.zip › HE×5(The second sample from each group) - 副本/Control.tif]

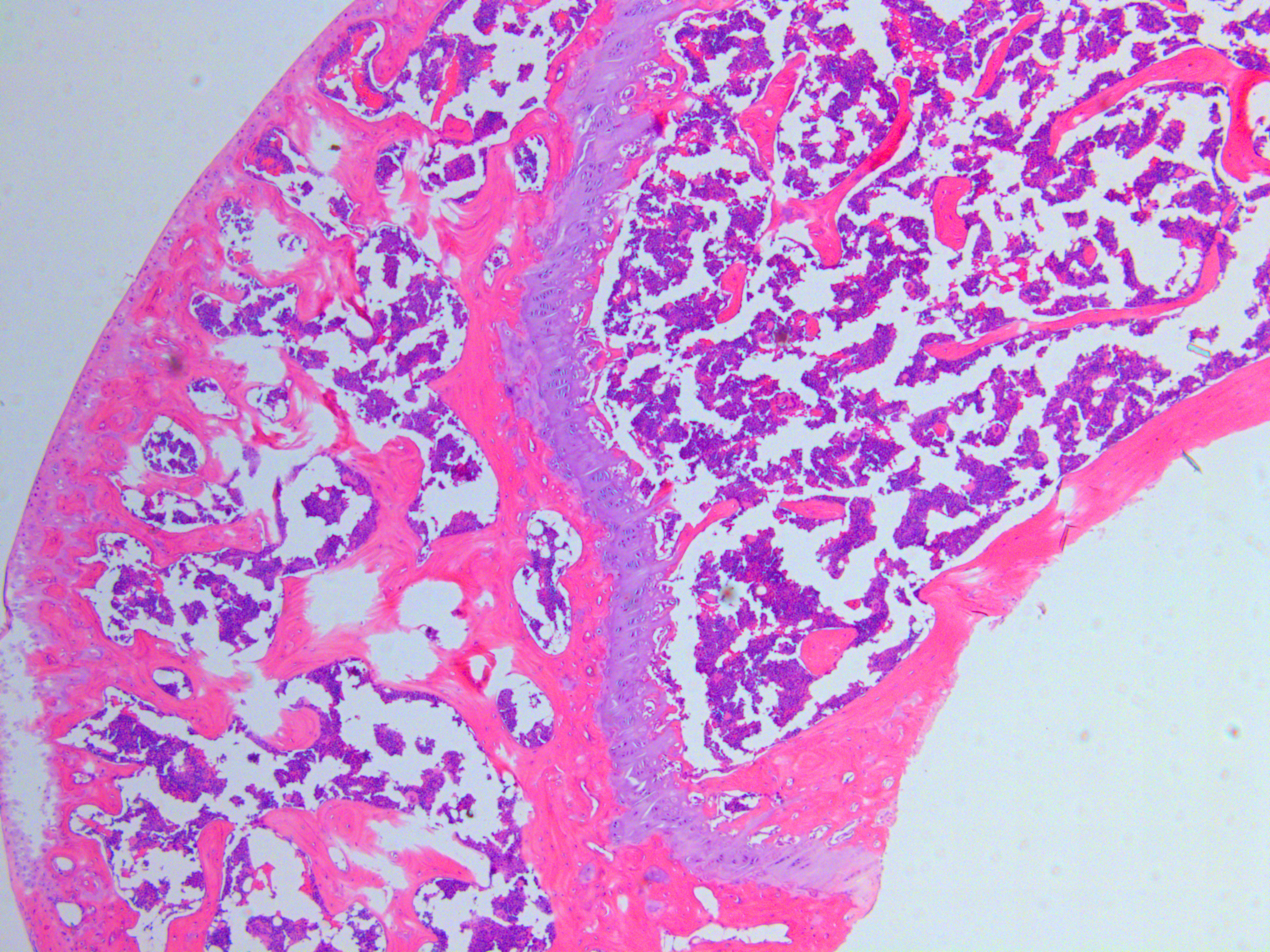

Supplement: Supplementary file 5 [file DataSheet9.zip › HE×5(The second sample from each group) - 副本/H-BGSSD.tif]

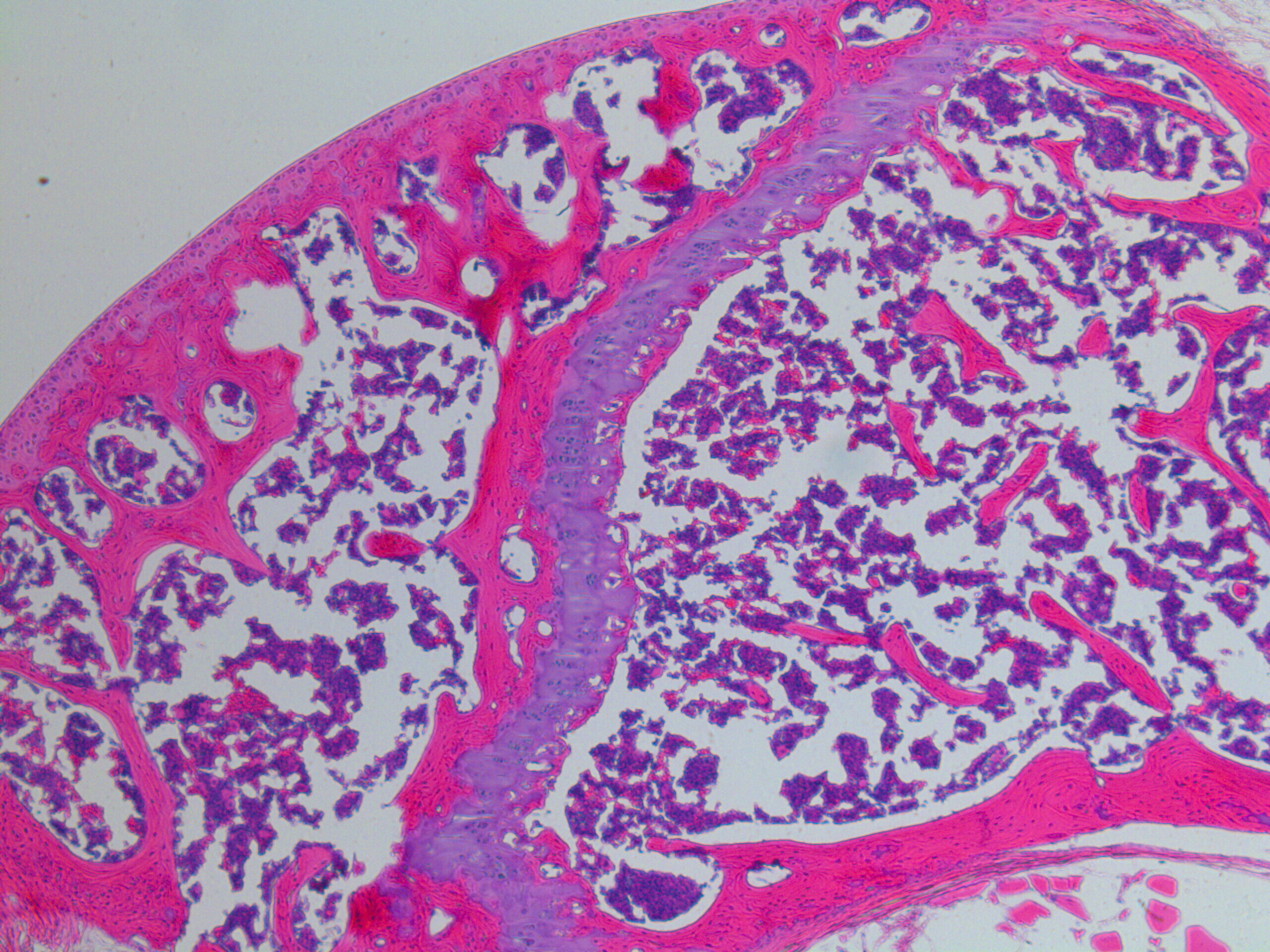

Supplement: Supplementary file 5 [file DataSheet9.zip › HE×5(The second sample from each group) - 副本/Model.jpg]

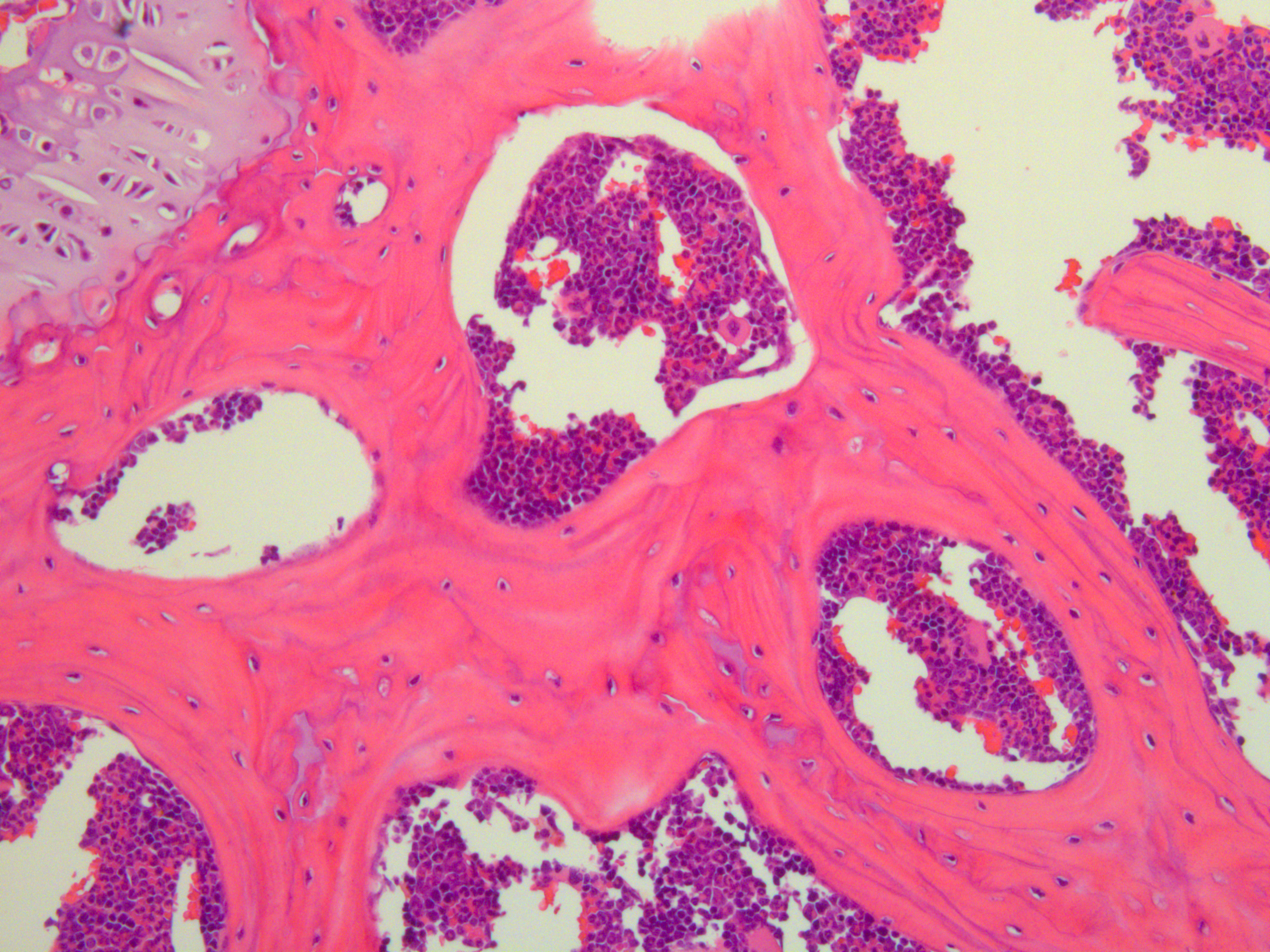

Supplement: Supplementary file 6 [file DataSheet4.zip › Figure2B-HE×20(The first sample from each group)/FIGURE 2B-HE(1)- (Alendronate).tif]

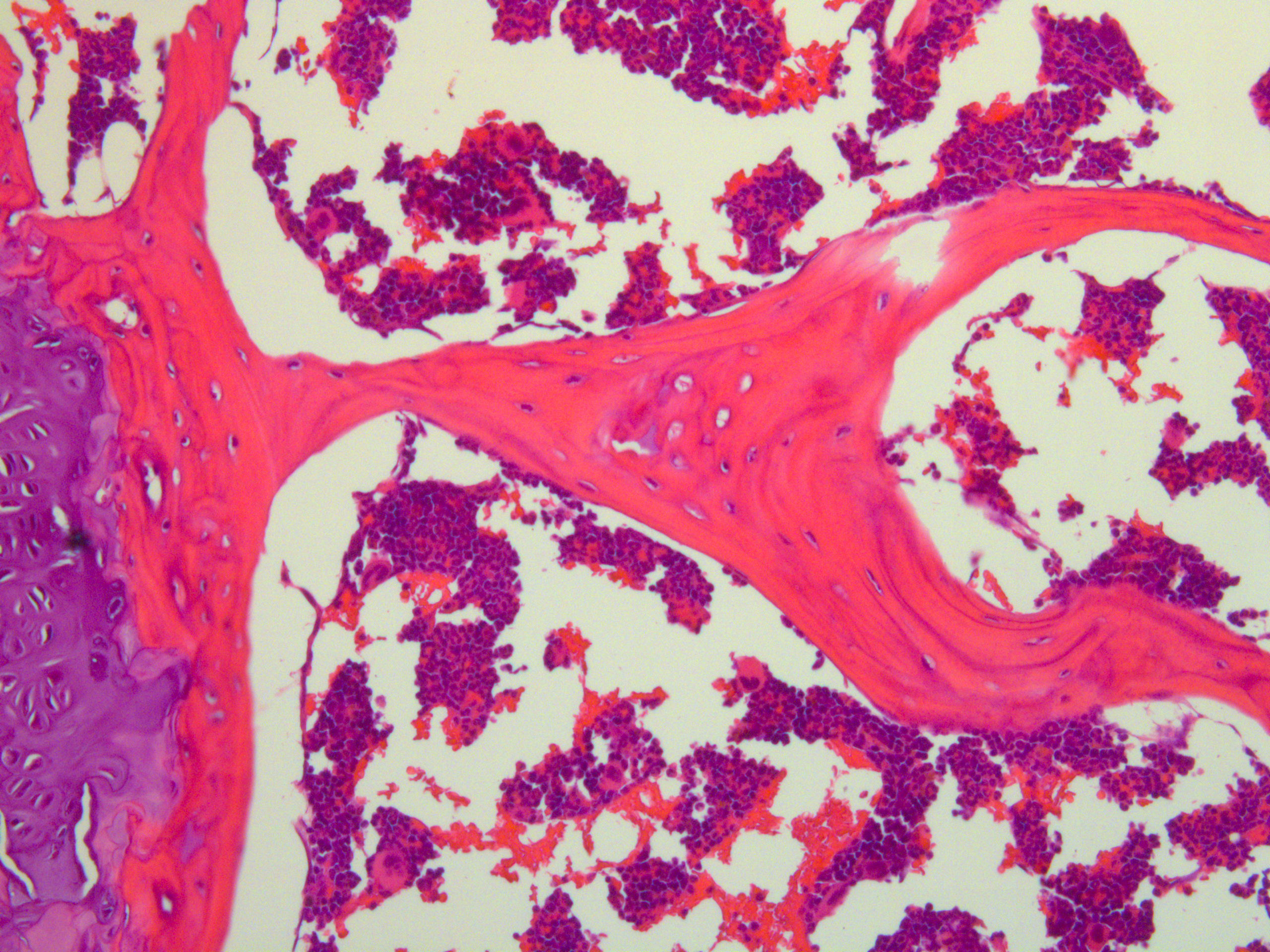

Supplement: Supplementary file 6 [file DataSheet4.zip › Figure2B-HE×20(The first sample from each group)/FIGURE 2B-HE(1)- (Model).tif]

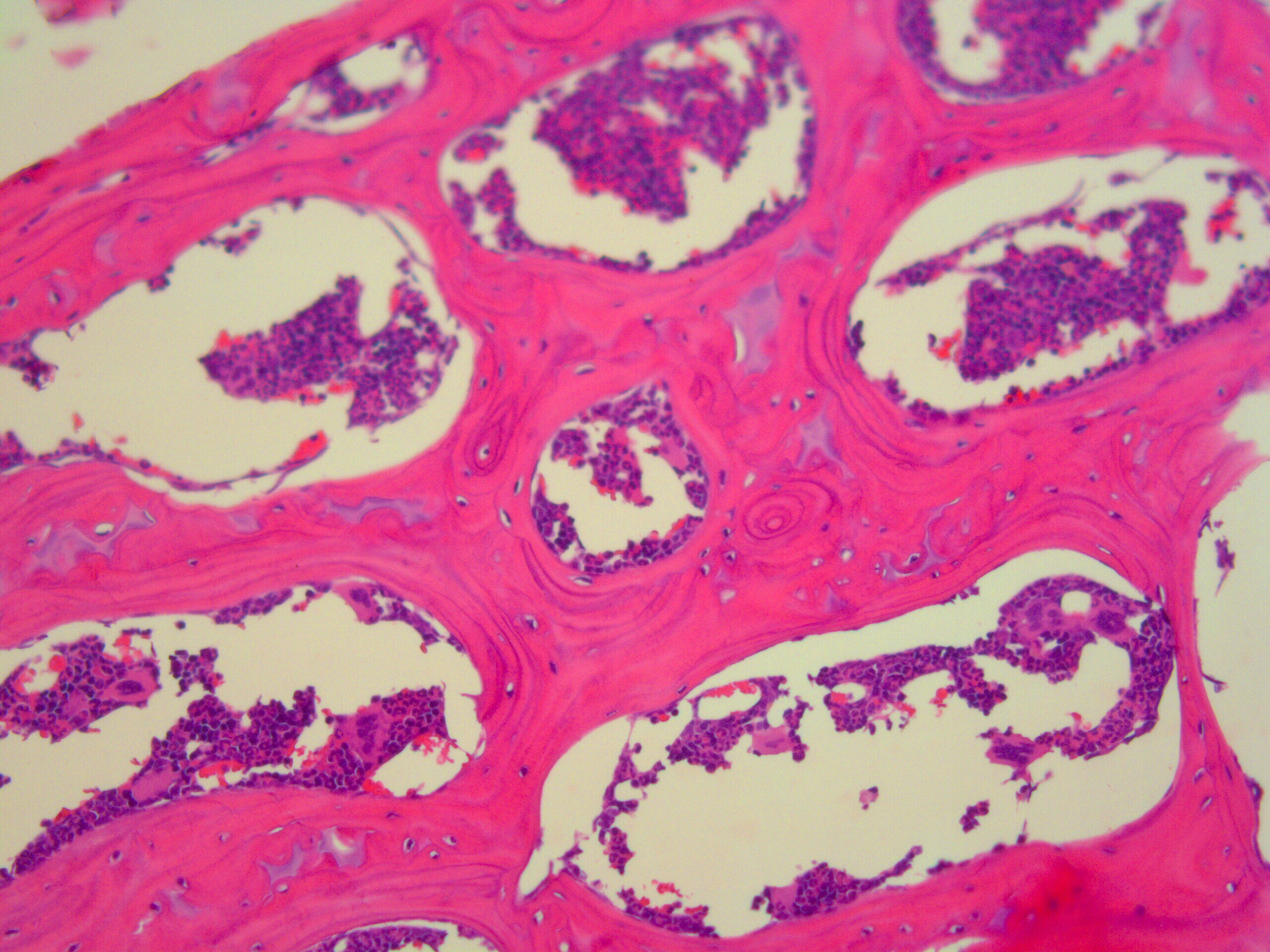

Supplement: Supplementary file 6 [file DataSheet4.zip › Figure2B-HE×20(The first sample from each group)/FIGURE 2B-HE(1)-(Control).jpg]

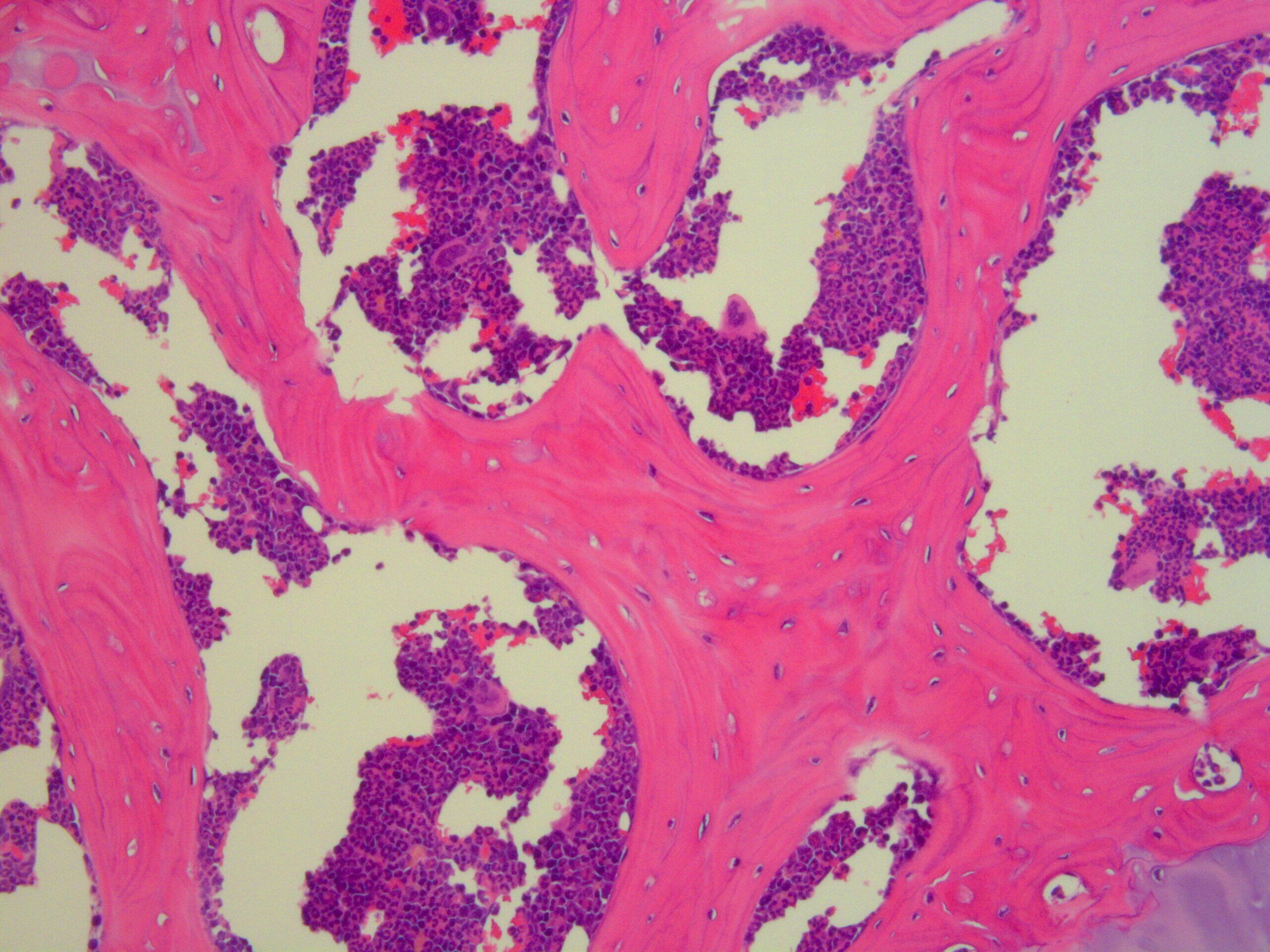

Supplement: Supplementary file 7 [file DataSheet13.zip › HE×20(The second sample from each group) - 副本/HE(2)- (Control).jpg]

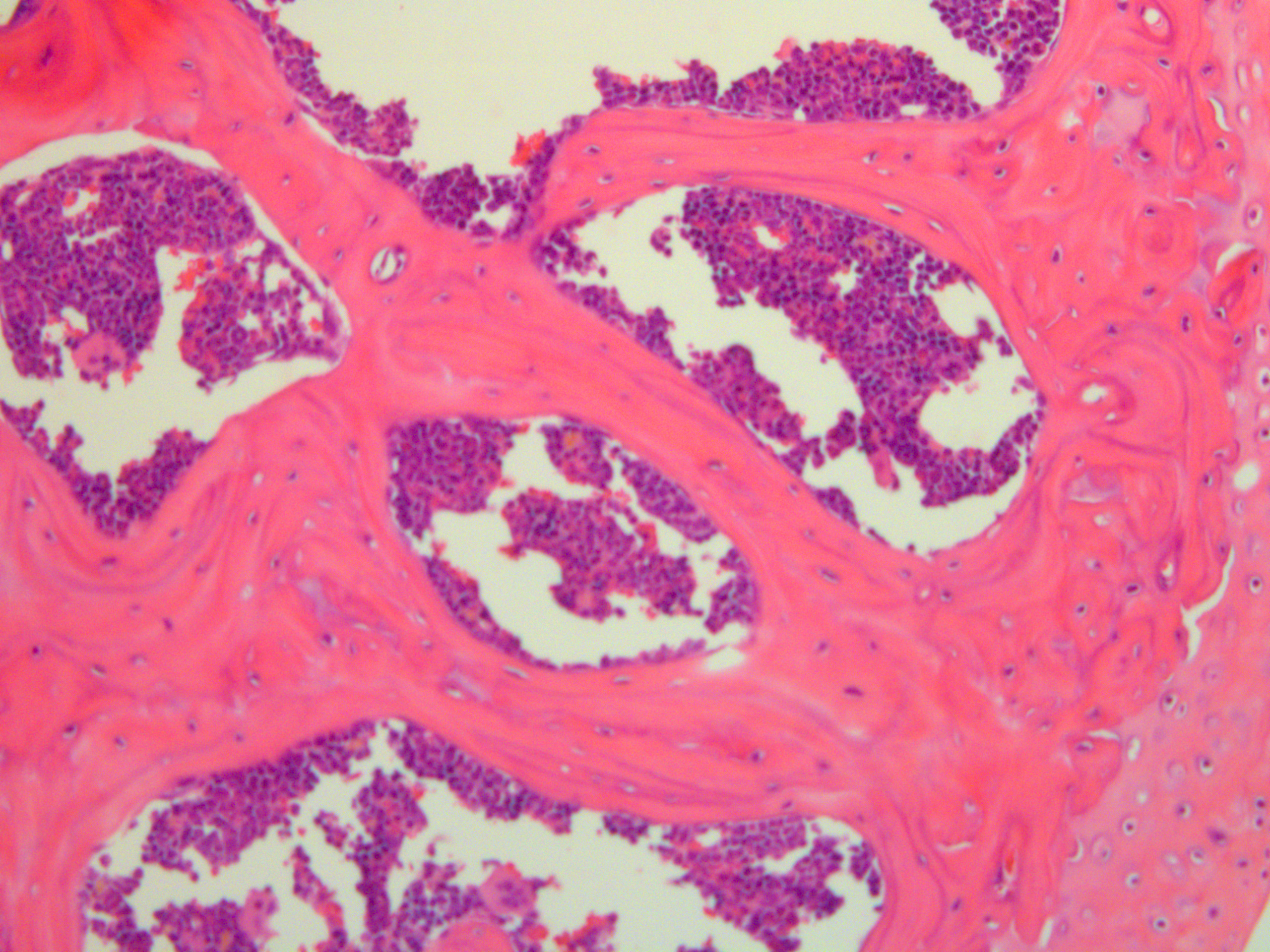

Supplement: Supplementary file 7 [file DataSheet13.zip › HE×20(The second sample from each group) - 副本/HE(2)-(Alendronate).tif]

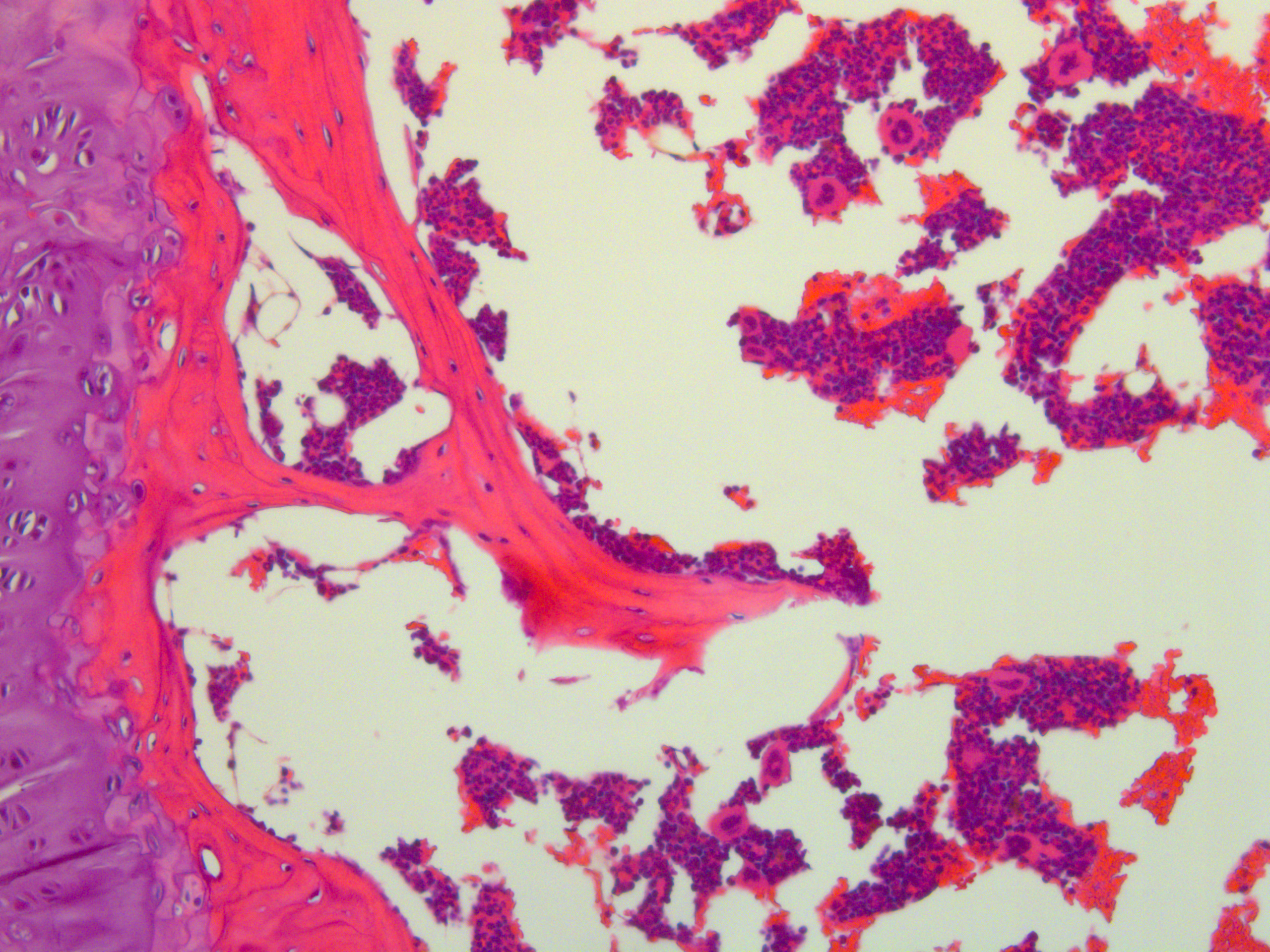

Supplement: Supplementary file 7 [file DataSheet13.zip › HE×20(The second sample from each group) - 副本/HE(2)-(Model).tif]

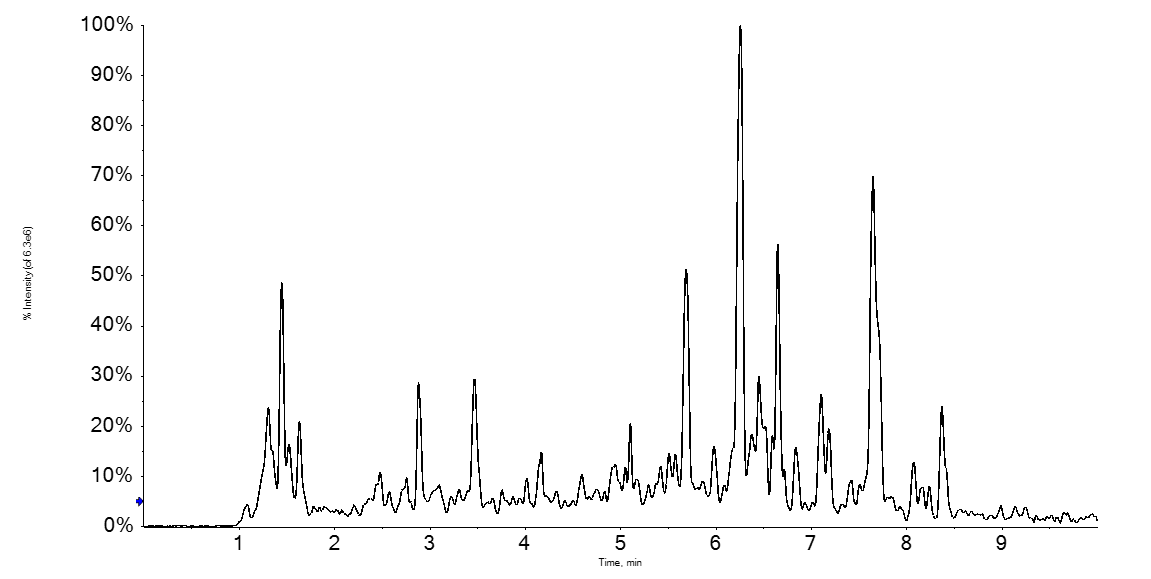

Supplement: Supplementary file 9 [file Image1.tif]

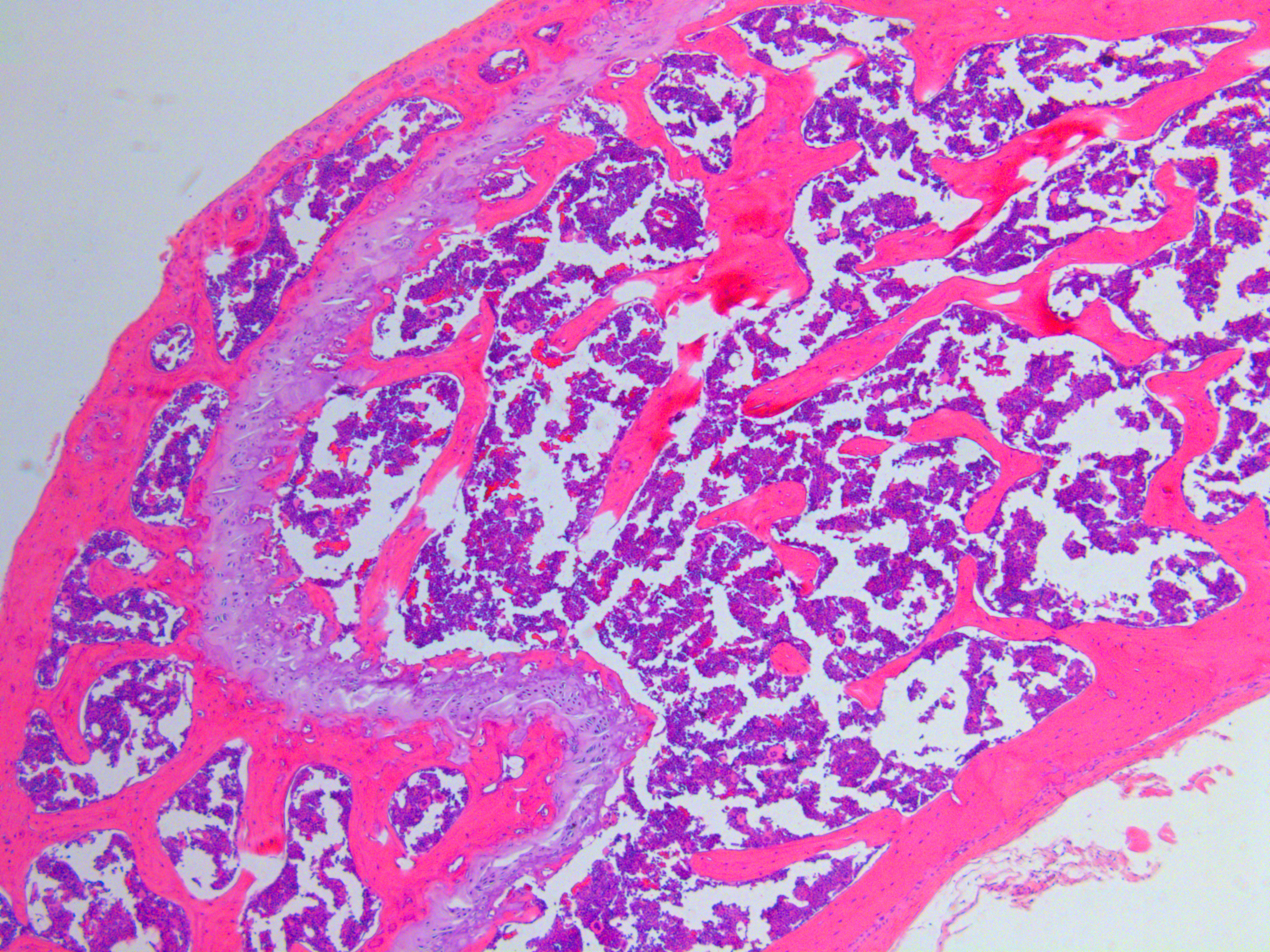

Supplement: Supplementary file 10 [file DataSheet10.zip › HE×5(The third sample from each group)/H-BGSSD.tif]

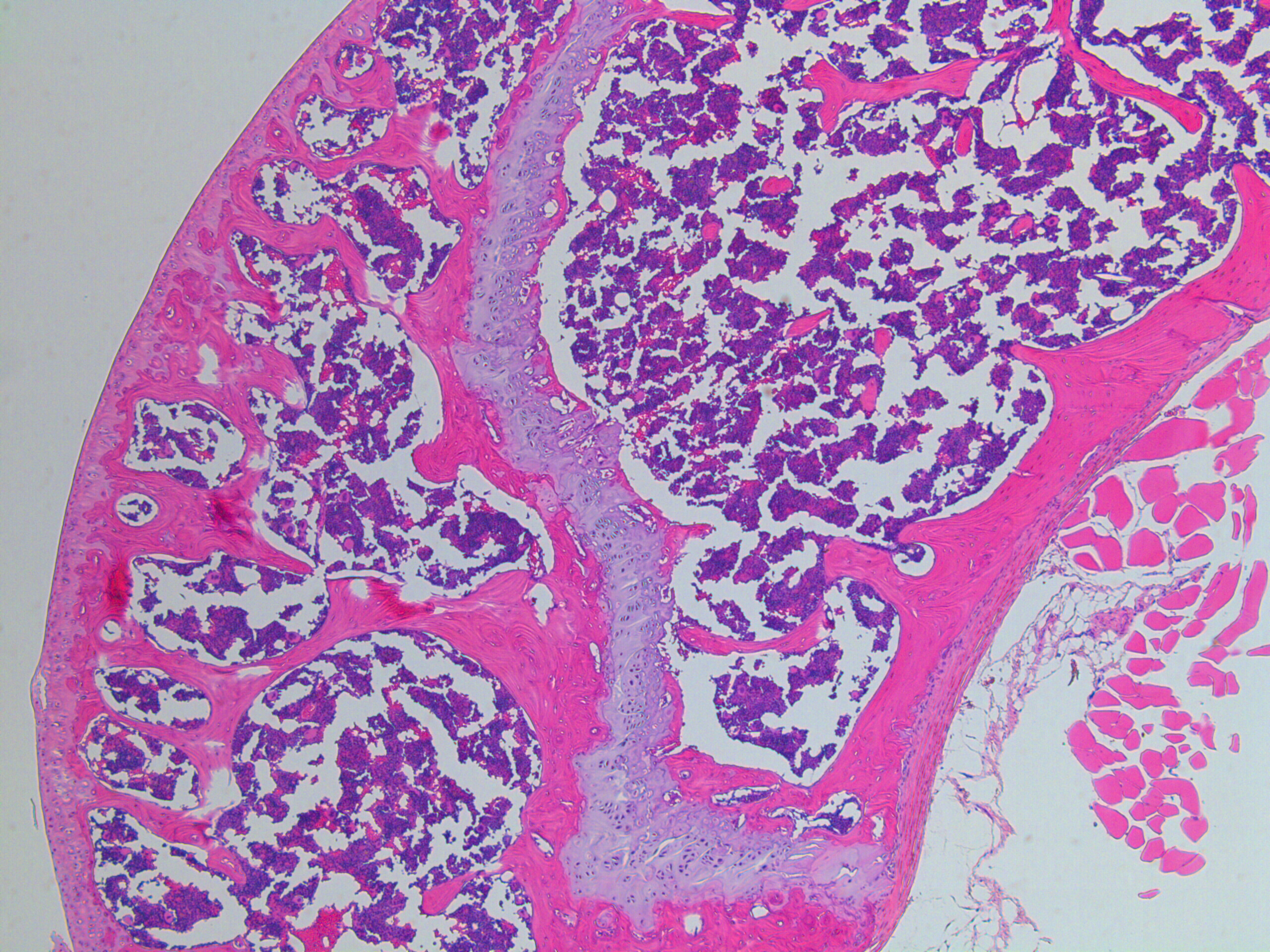

Supplement: Supplementary file 10 [file DataSheet10.zip › HE×5(The third sample from each group)/L-BGSSD.jpg]

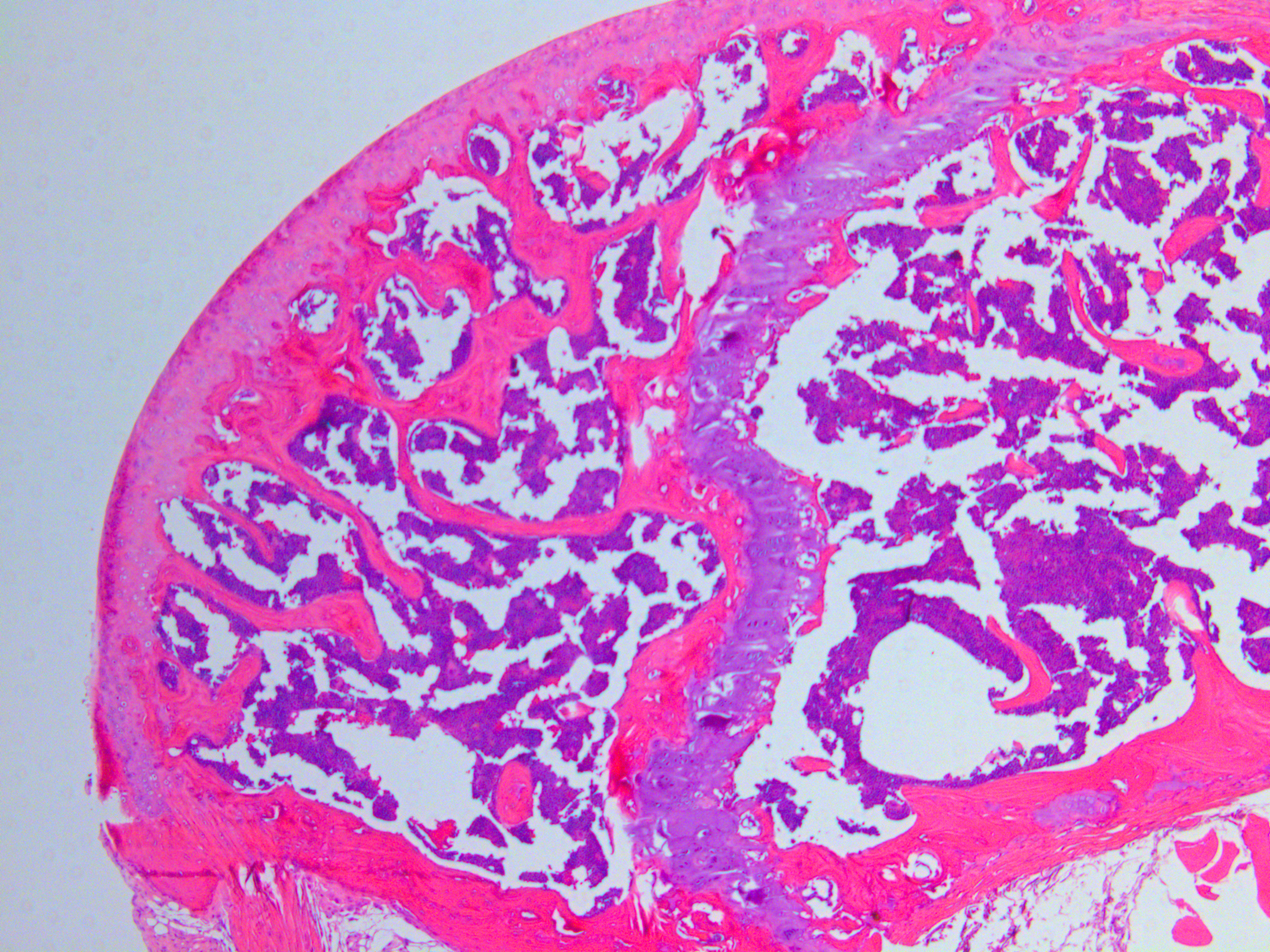

Supplement: Supplementary file 10 [file DataSheet10.zip › HE×5(The third sample from each group)/M- BGSSD.tif]

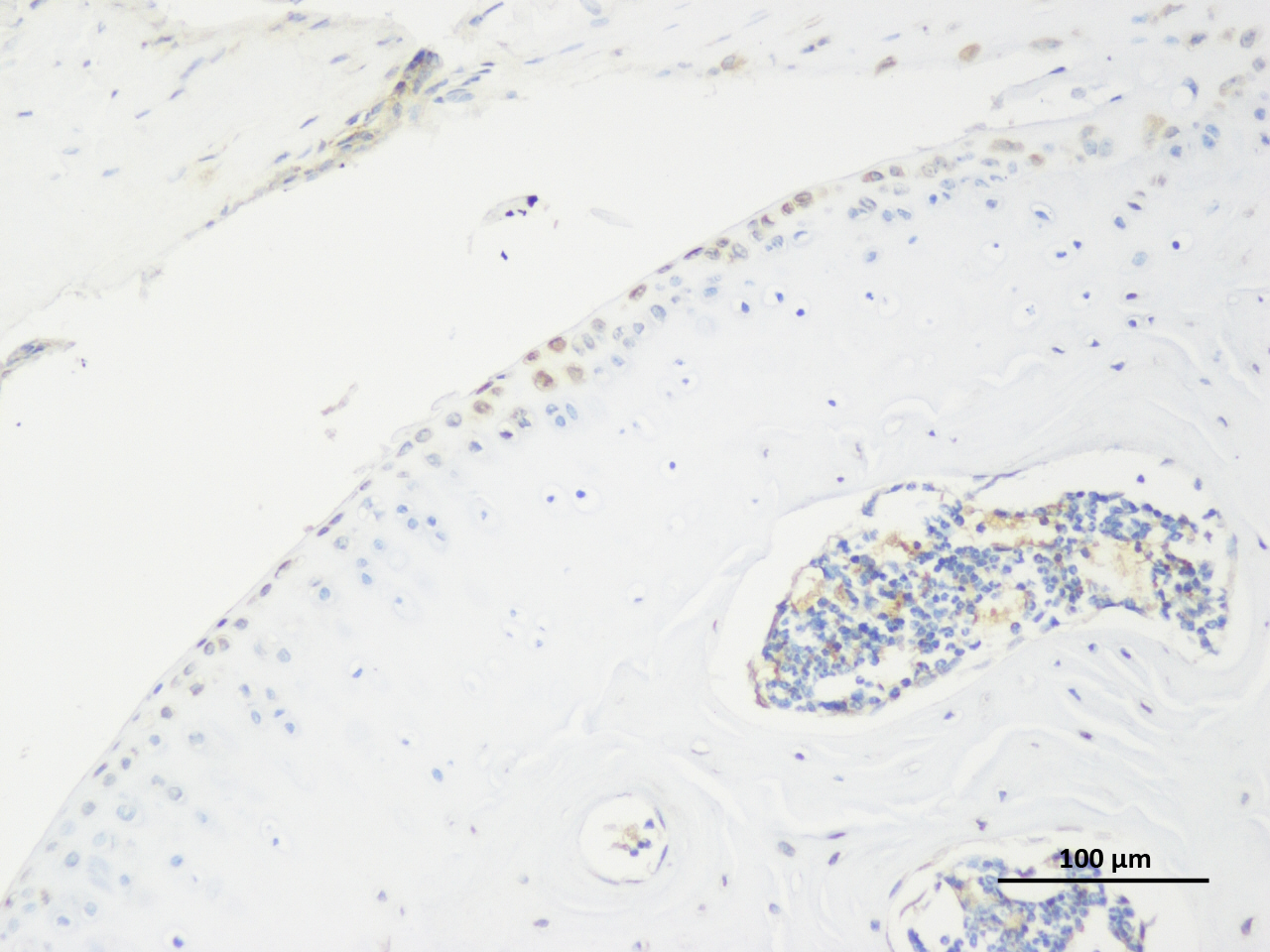

Supplement: Supplementary file 11 [file DataSheet6.zip › Figure4 A, C-IHC staining/Immunohistochemical staining(The first sample from each group)/ERK1-2 (Control).jpg]

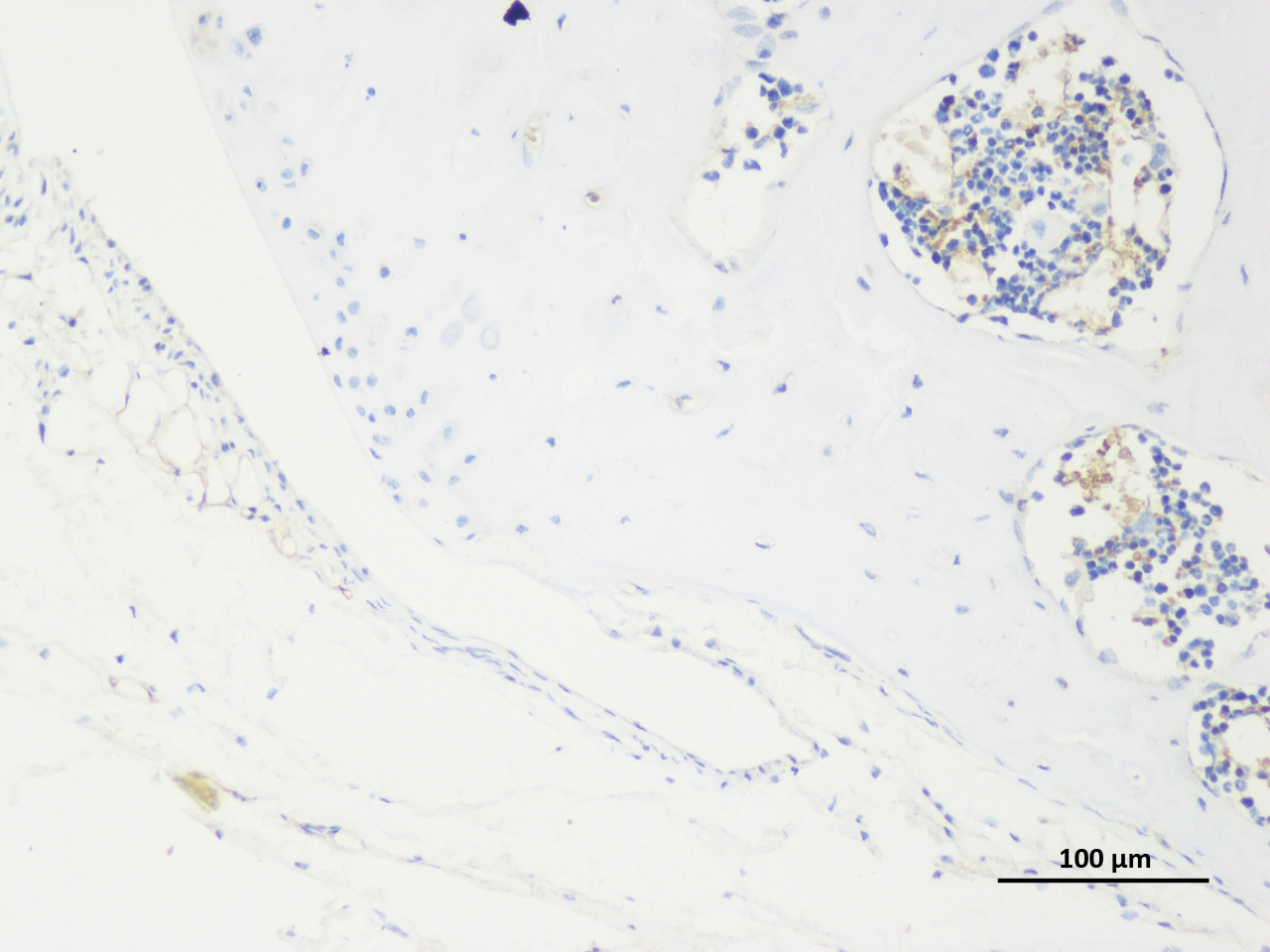

Supplement: Supplementary file 11 [file DataSheet6.zip › Figure4 A, C-IHC staining/Immunohistochemical staining(The first sample from each group)/ERK1-2 (H-BGSSD).jpg]

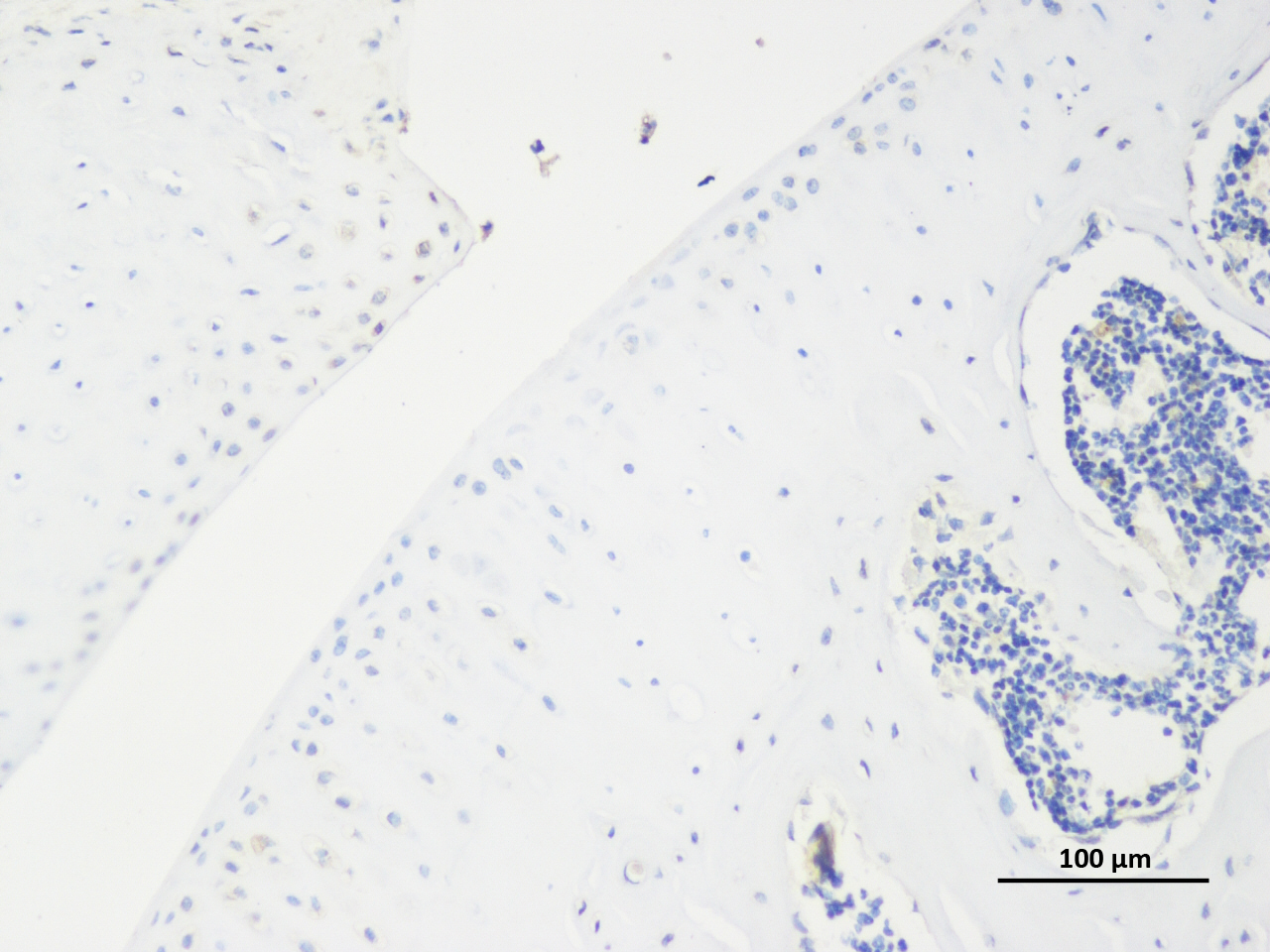

Supplement: Supplementary file 11 [file DataSheet6.zip › Figure4 A, C-IHC staining/Immunohistochemical staining(The first sample from each group)/ERK1-2 (Model).jpg]

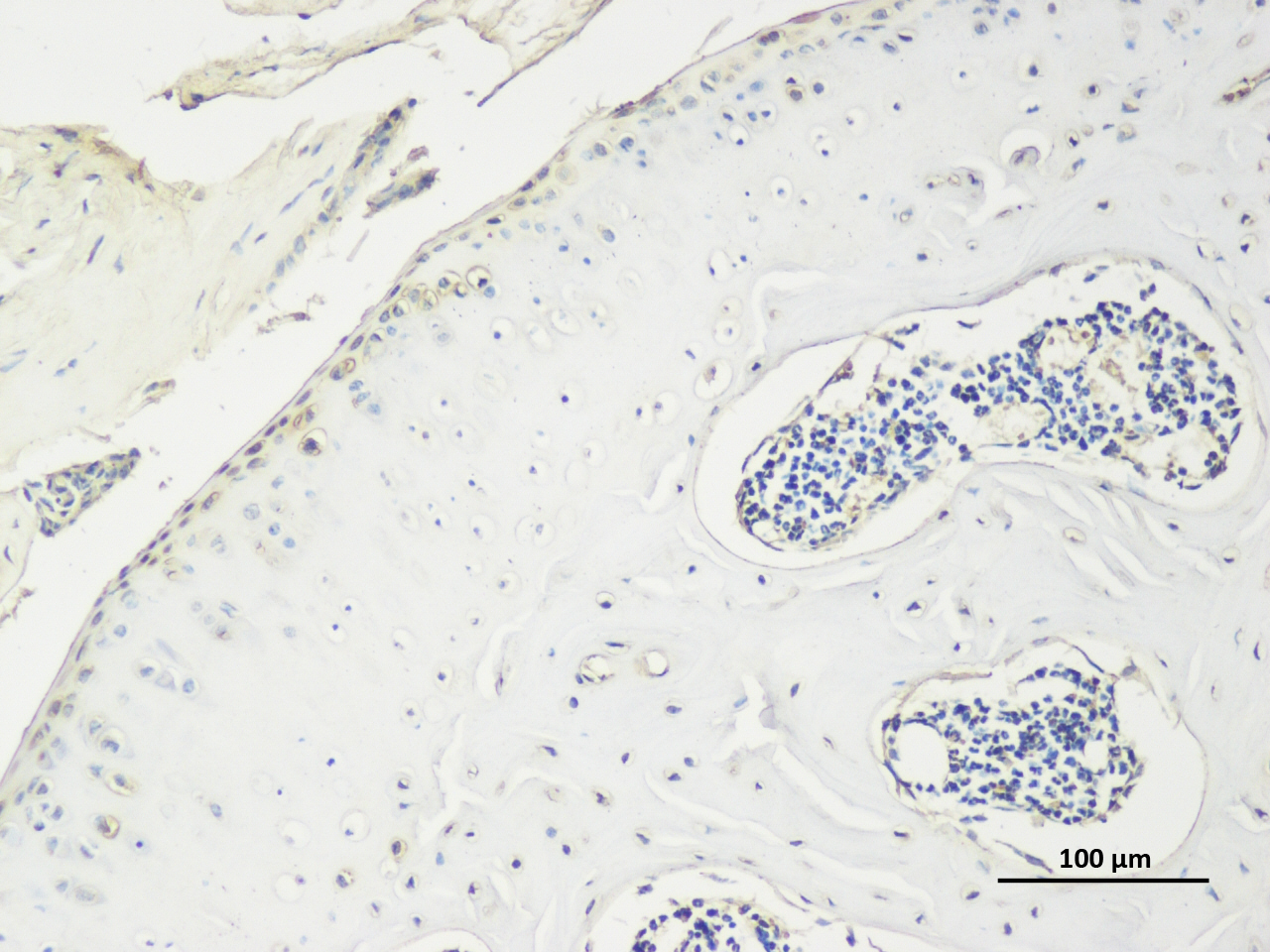

Supplement: Supplementary file 11 [file DataSheet6.zip › Figure4 A, C-IHC staining/Immunohistochemical staining(The first sample from each group)/Smad4 (Control).jpg]

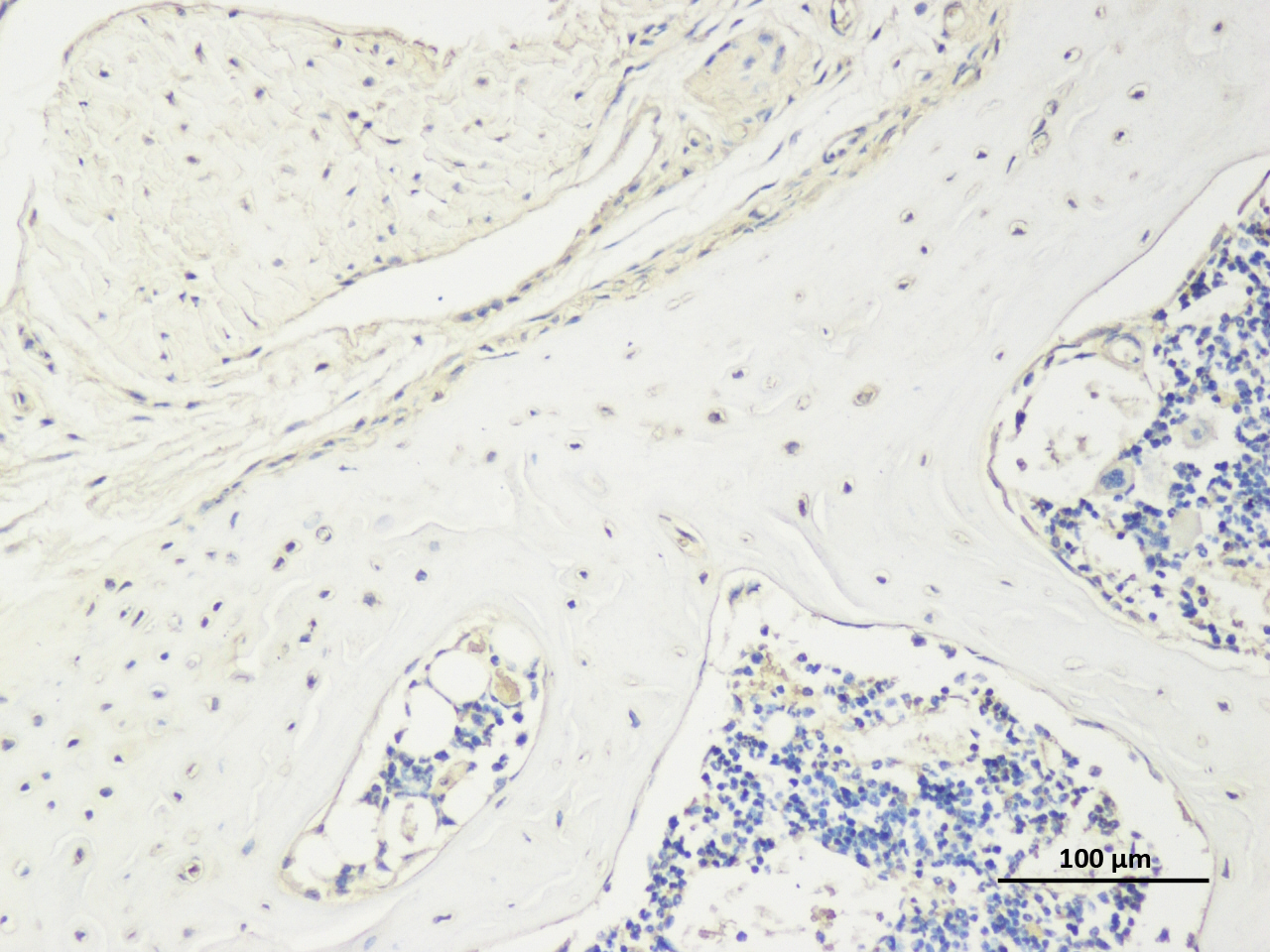

Supplement: Supplementary file 11 [file DataSheet6.zip › Figure4 A, C-IHC staining/Immunohistochemical staining(The first sample from each group)/Smad4 (H-BGSSD).jpg]

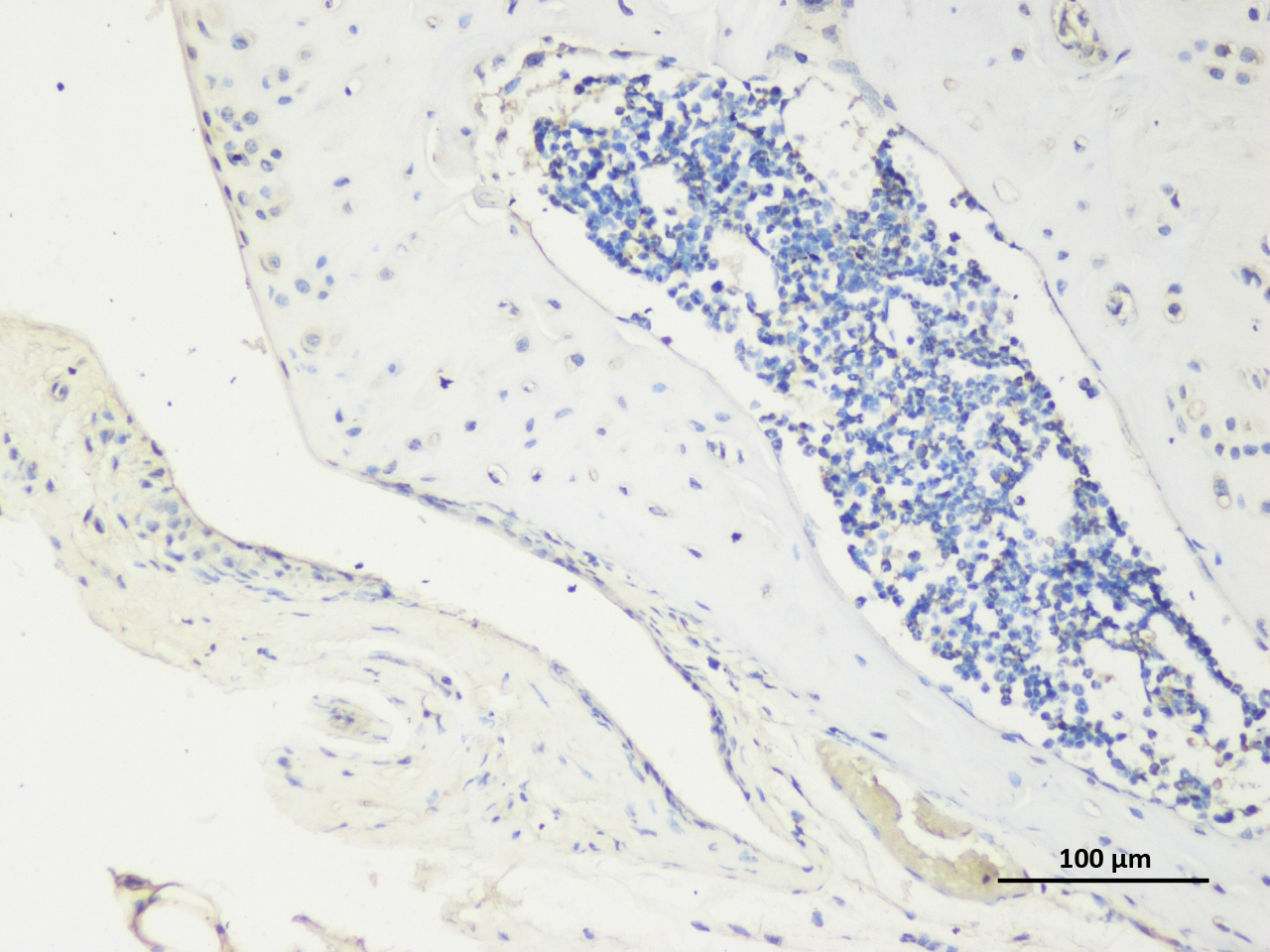

Supplement: Supplementary file 11 [file DataSheet6.zip › Figure4 A, C-IHC staining/Immunohistochemical staining(The first sample from each group)/Smad4 (Model).jpg]

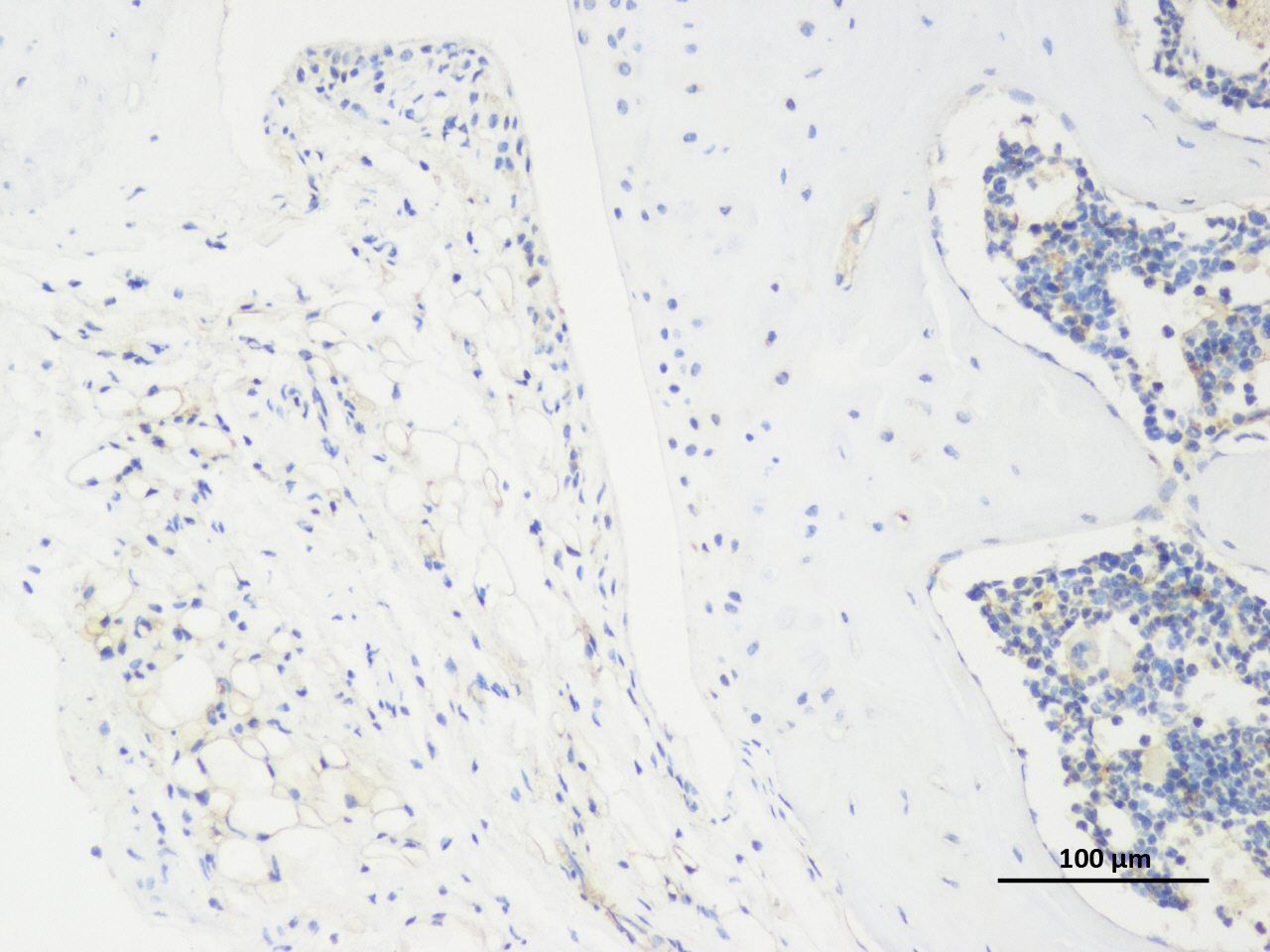

Supplement: Supplementary file 11 [file DataSheet6.zip › Figure4 A, C-IHC staining/Immunohistochemical staining(The second sample from each group)/ERK1-2 (Control) .jpg]

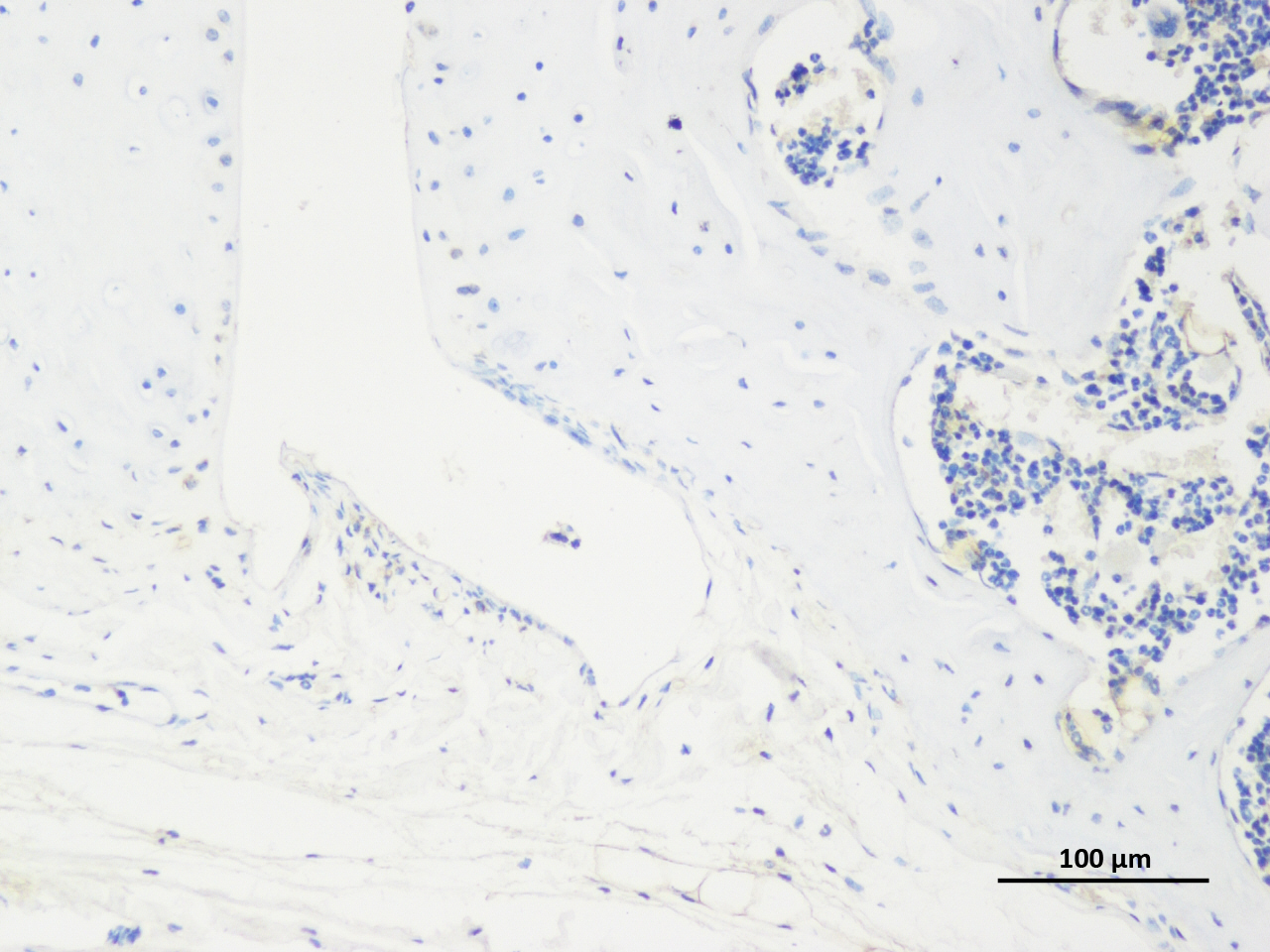

Supplement: Supplementary file 11 [file DataSheet6.zip › Figure4 A, C-IHC staining/Immunohistochemical staining(The second sample from each group)/ERK1-2 (Model) .jpg]

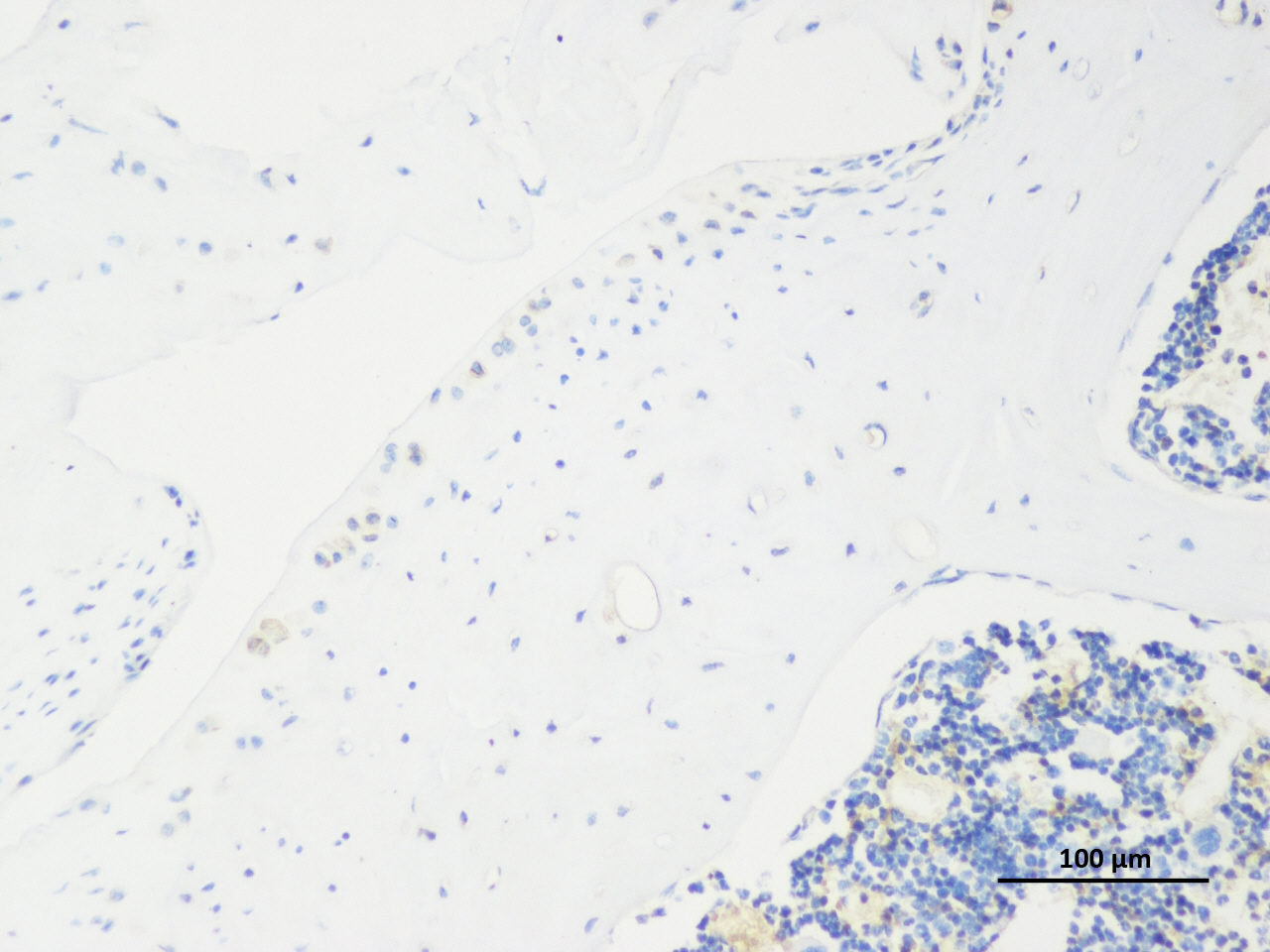

Supplement: Supplementary file 11 [file DataSheet6.zip › Figure4 A, C-IHC staining/Immunohistochemical staining(The second sample from each group)/ERK1-2 (H-BGSSD).jpg]

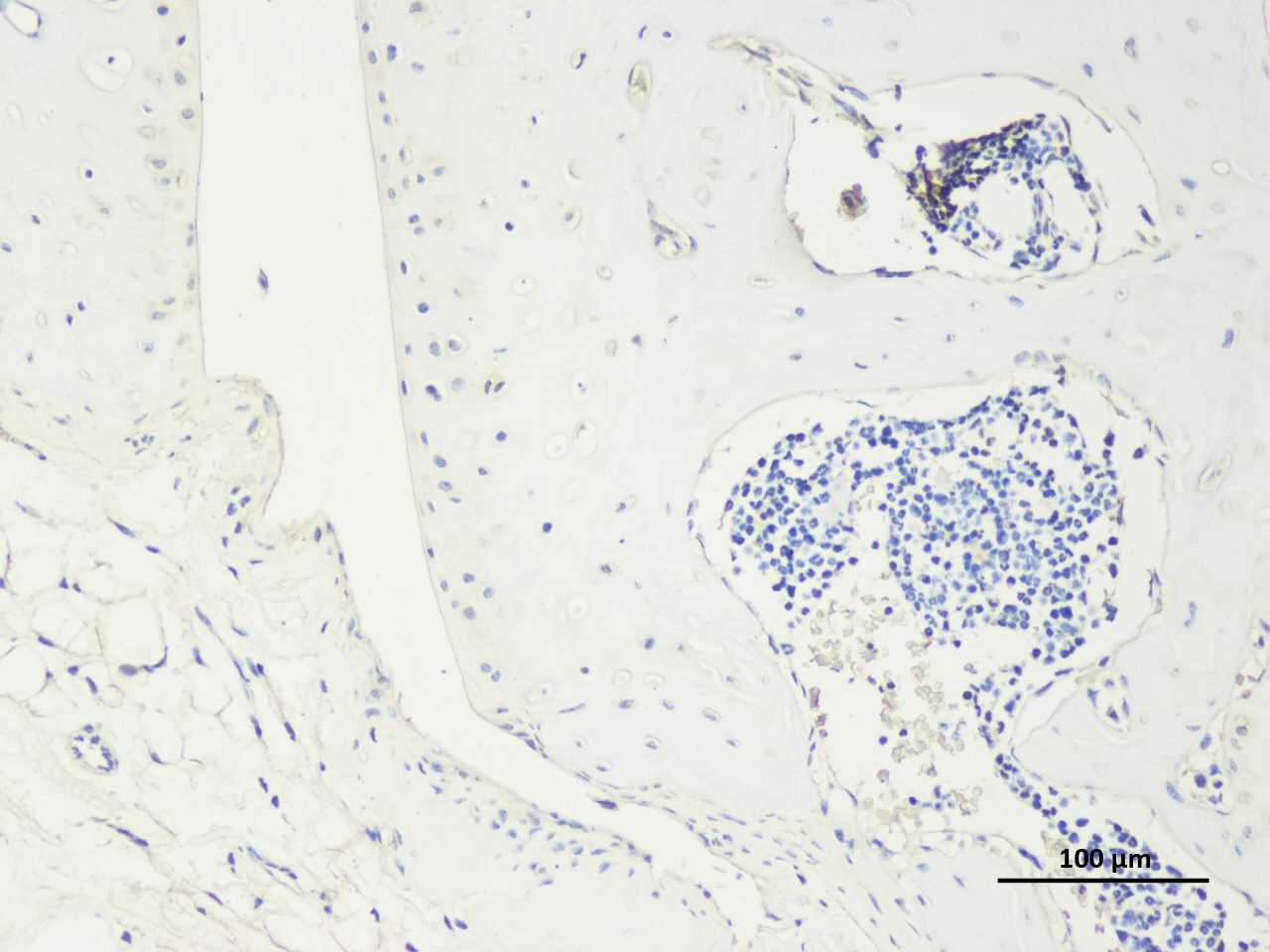

Supplement: Supplementary file 11 [file DataSheet6.zip › Figure4 A, C-IHC staining/Immunohistochemical staining(The second sample from each group)/Smad4 (Control).jpg]

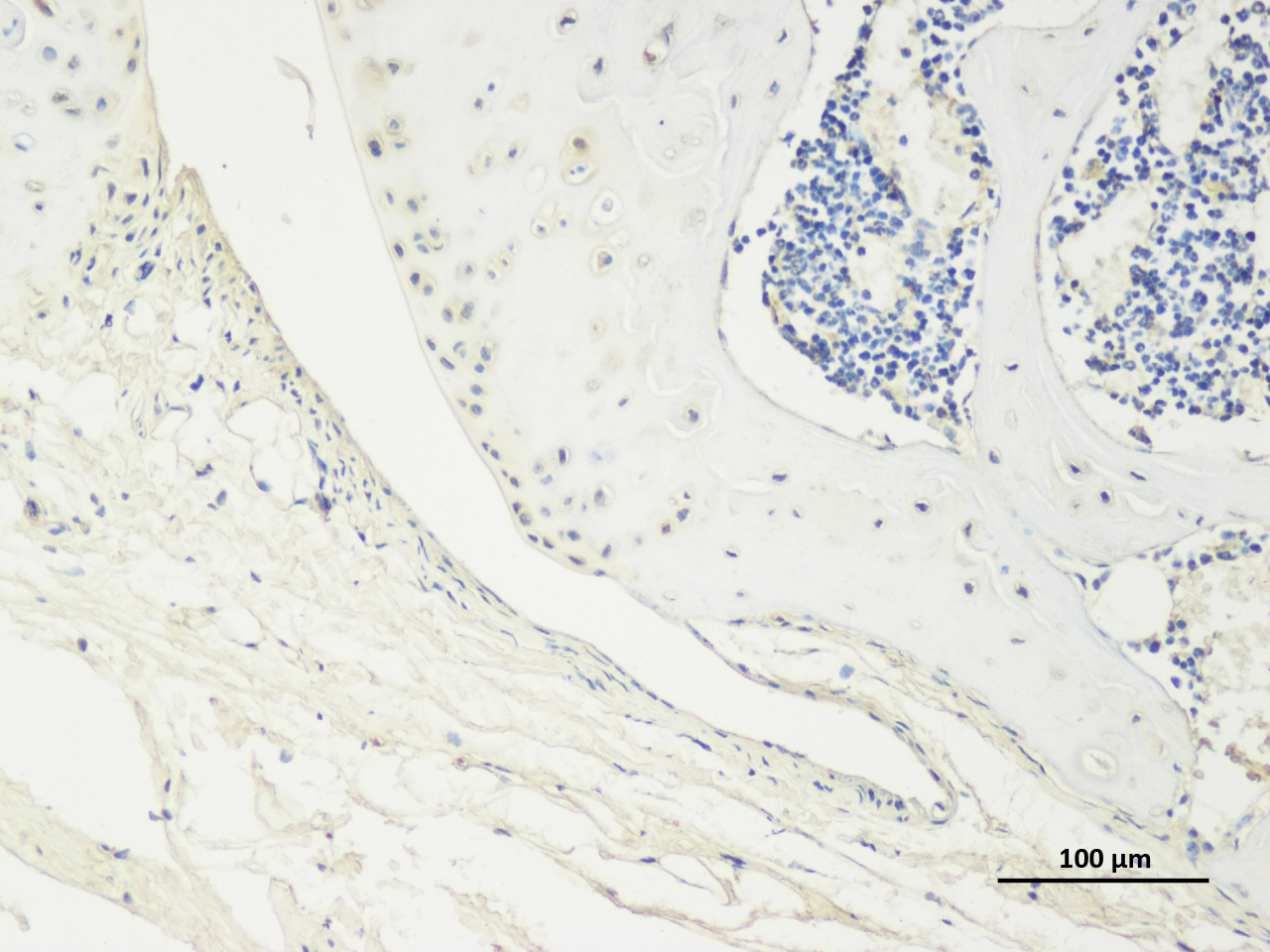

Supplement: Supplementary file 11 [file DataSheet6.zip › Figure4 A, C-IHC staining/Immunohistochemical staining(The second sample from each group)/Smad4 (H-BGSSD).jpg]

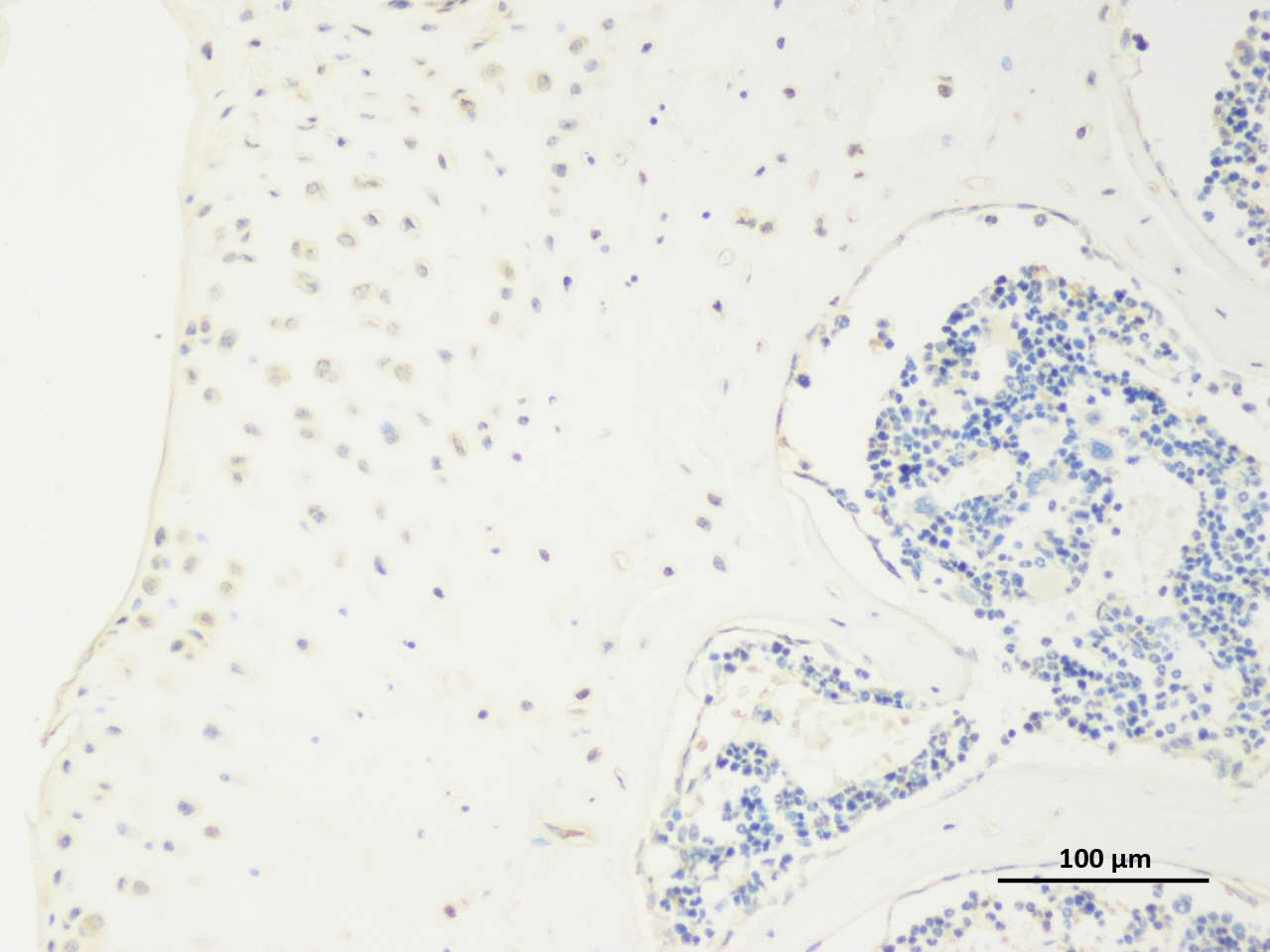

Supplement: Supplementary file 11 [file DataSheet6.zip › Figure4 A, C-IHC staining/Immunohistochemical staining(The second sample from each group)/Smad4 (Model).jpg]

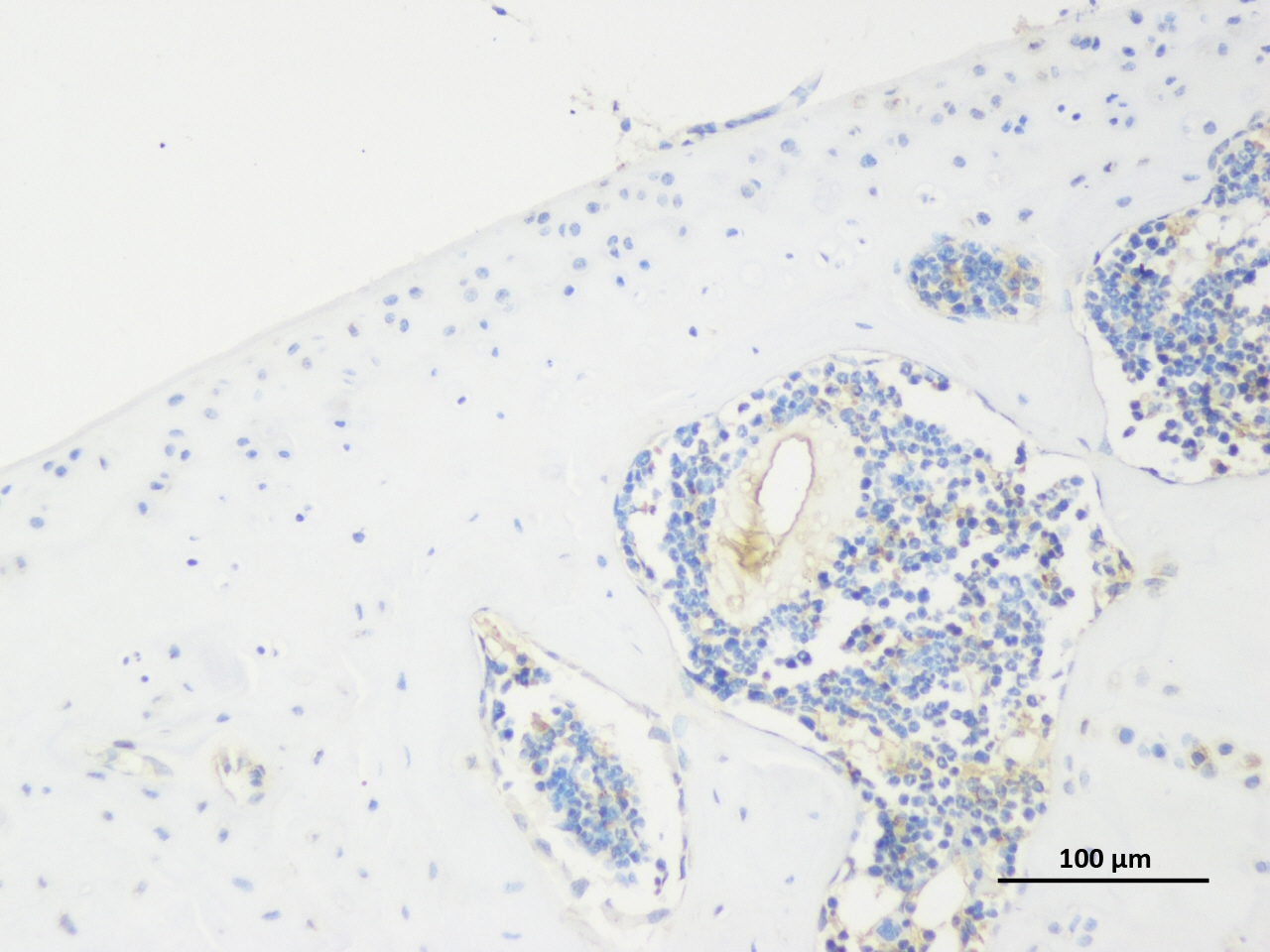

Supplement: Supplementary file 11 [file DataSheet6.zip › Figure4 A, C-IHC staining/Immunohistochemical staining(The third sample from each group)/ERK1-2(Control).jpg]

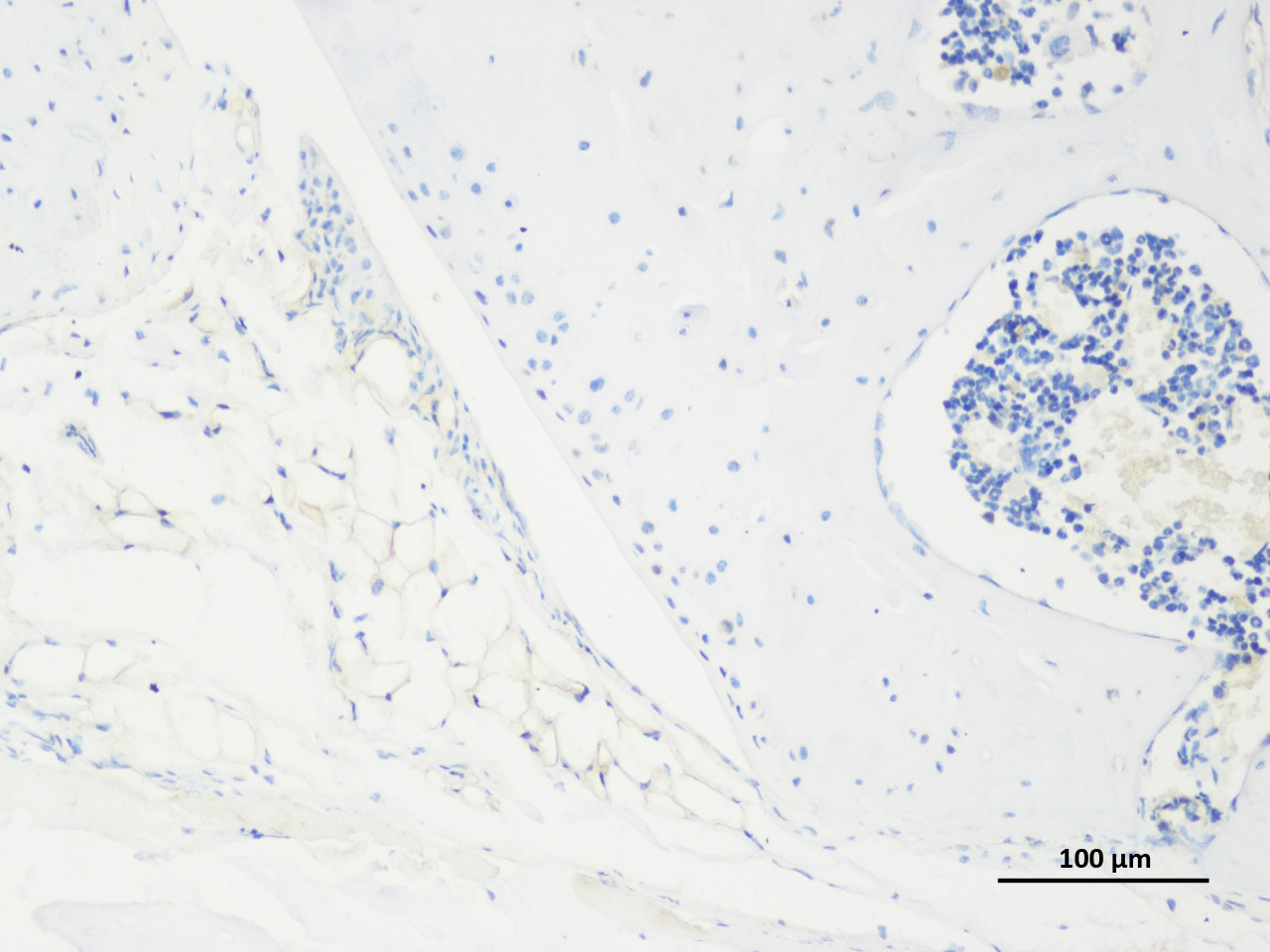

Supplement: Supplementary file 11 [file DataSheet6.zip › Figure4 A, C-IHC staining/Immunohistochemical staining(The third sample from each group)/ERK1-2(H-BGSSD).jpg]

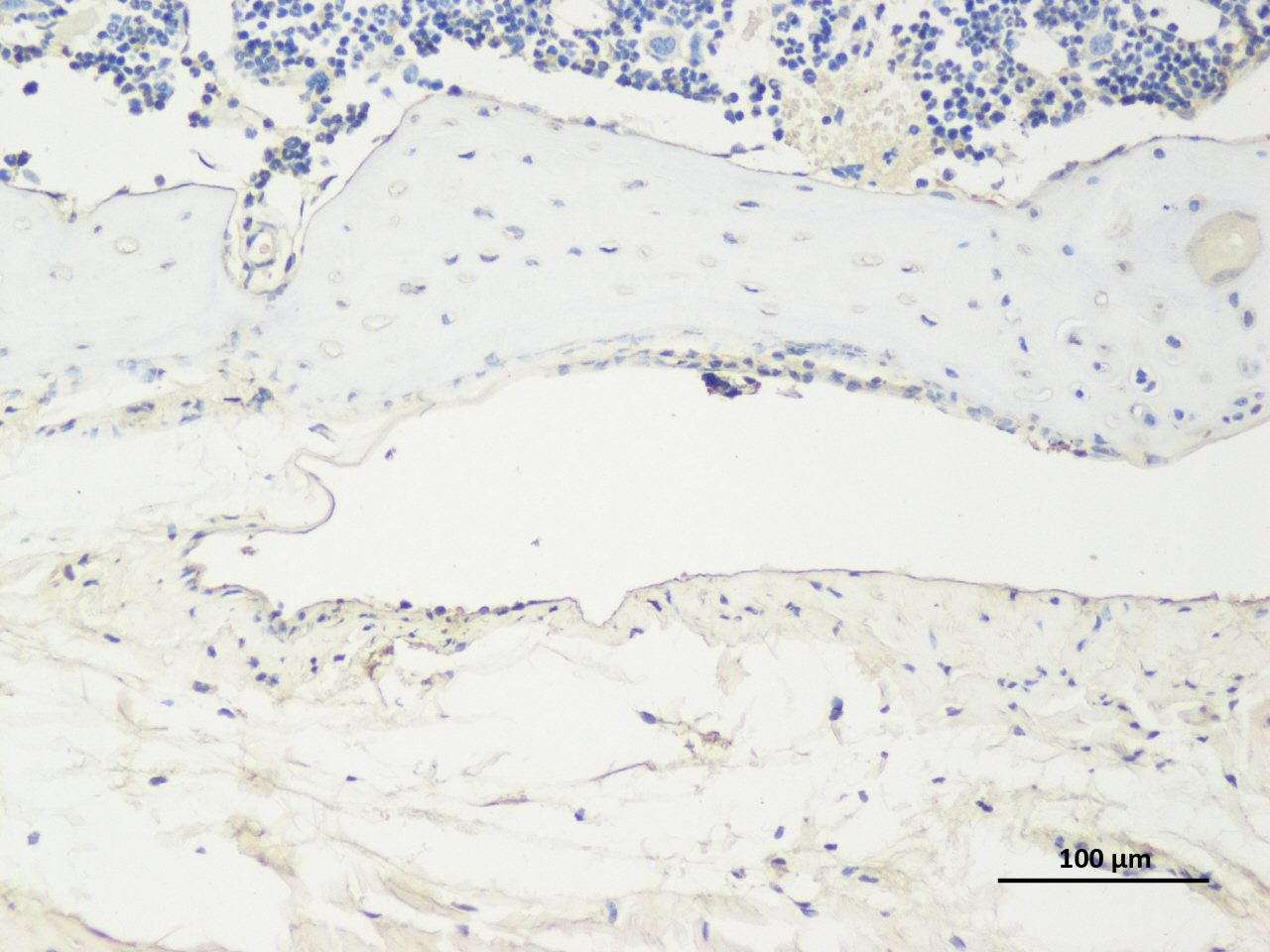

Supplement: Supplementary file 11 [file DataSheet6.zip › Figure4 A, C-IHC staining/Immunohistochemical staining(The third sample from each group)/Smad4 (Control).jpg]

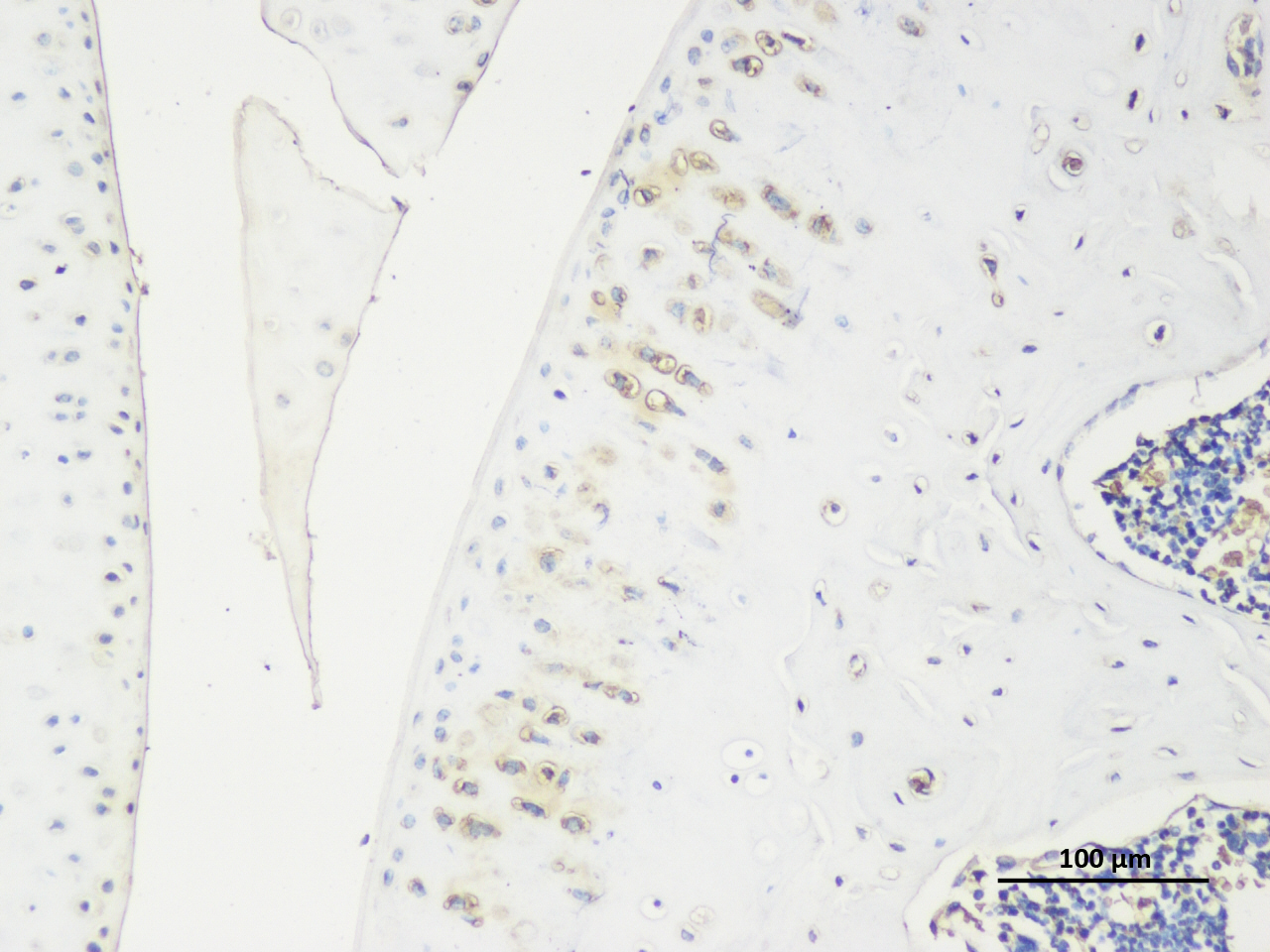

Supplement: Supplementary file 11 [file DataSheet6.zip › Figure4 A, C-IHC staining/Immunohistochemical staining(The third sample from each group)/Smad4 (H-BGSSD).jpg]

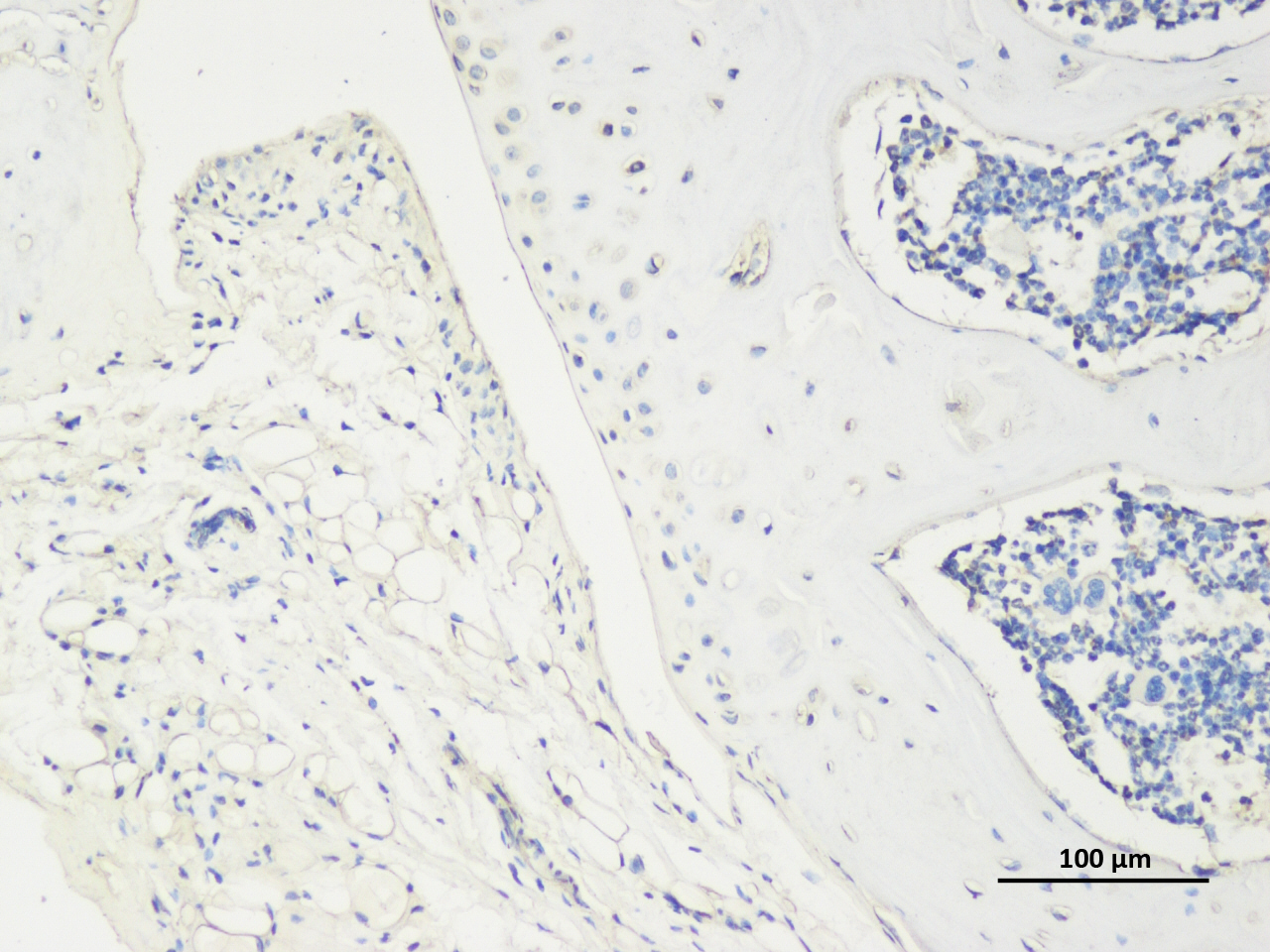

Supplement: Supplementary file 11 [file DataSheet6.zip › Figure4 A, C-IHC staining/Immunohistochemical staining(The third sample from each group)/Smad4 (Model).jpg]

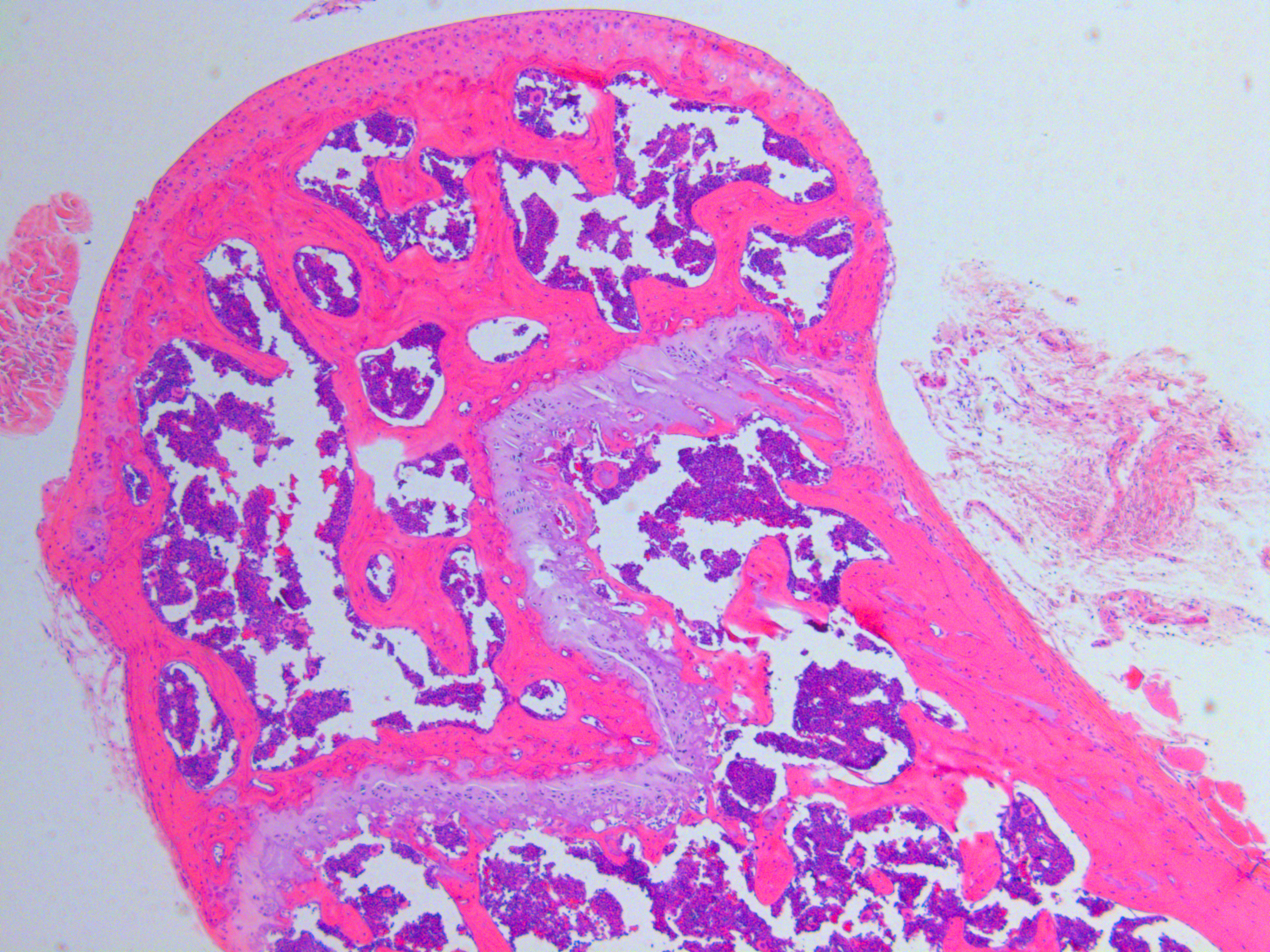

Supplement: Supplementary file 13 [file DataSheet2.zip › Figure2A-HE×5(The first sample from each group)/Alendronate.tif]

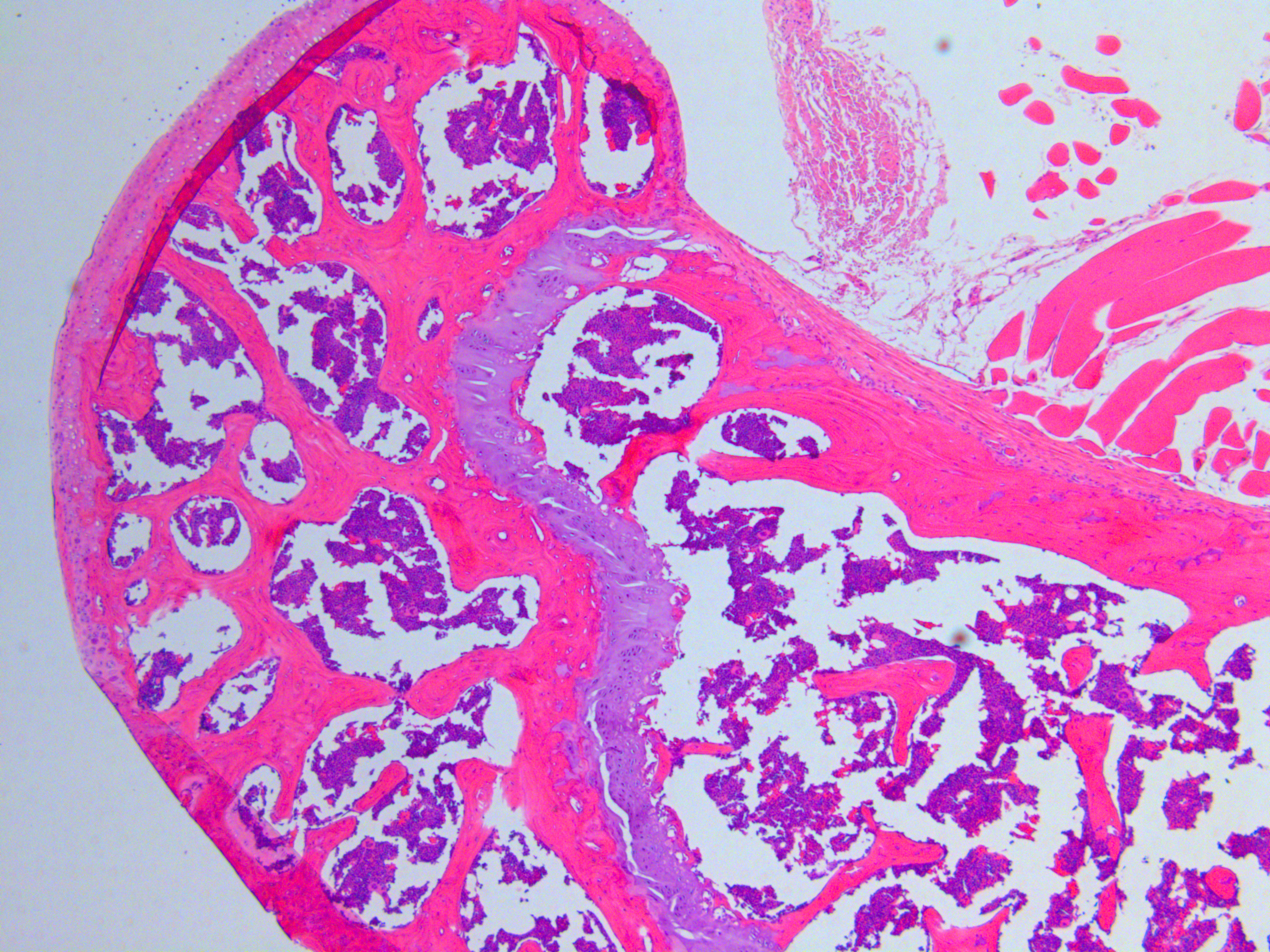

Supplement: Supplementary file 13 [file DataSheet2.zip › Figure2A-HE×5(The first sample from each group)/Control.tif]

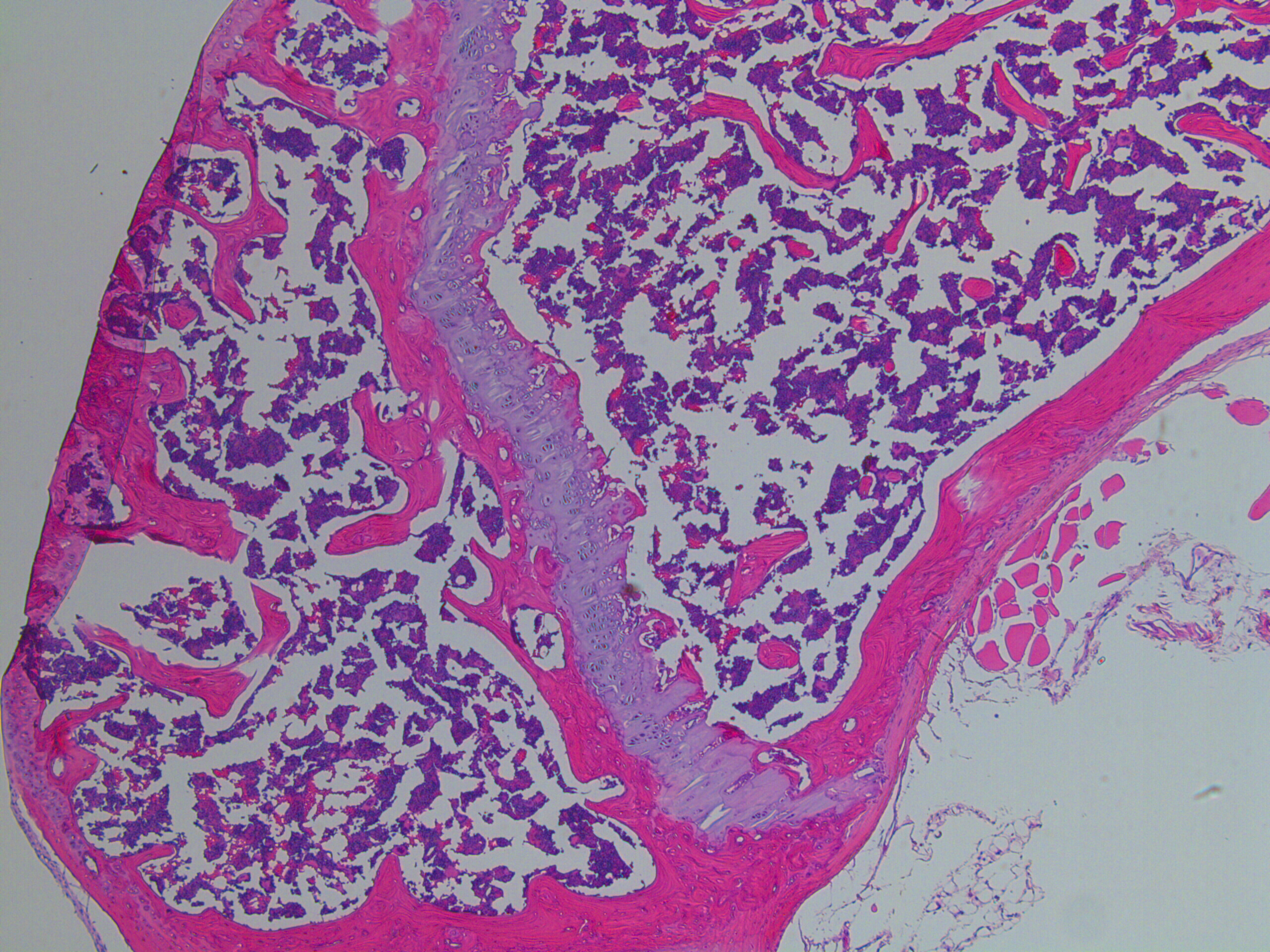

Supplement: Supplementary file 13 [file DataSheet2.zip › Figure2A-HE×5(The first sample from each group)/Model.jpg]

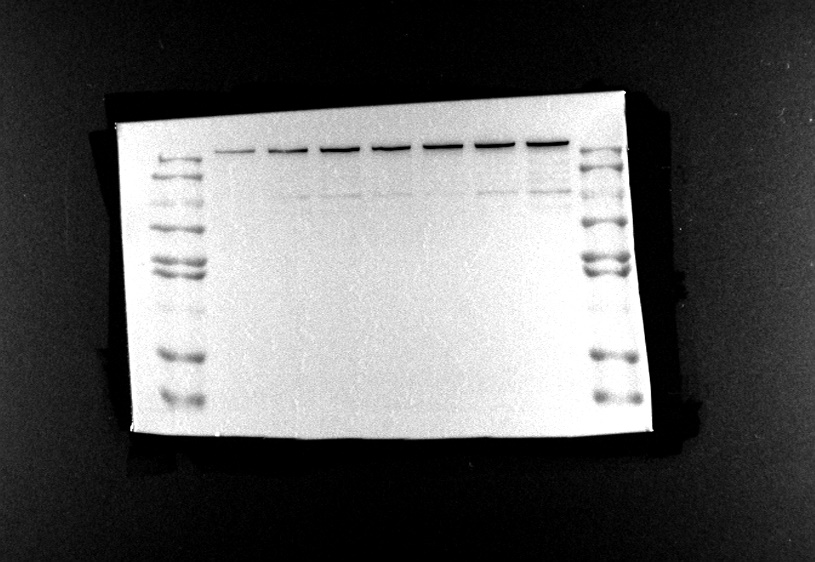

Supplement: Supplementary file 16 [file DataSheet7.zip › Figure 5 A,D-WB bands of ERK and Smad in vivo and vitro/FIGURE5-WB bands in vitro/The second group of samples- ERK12.tif]

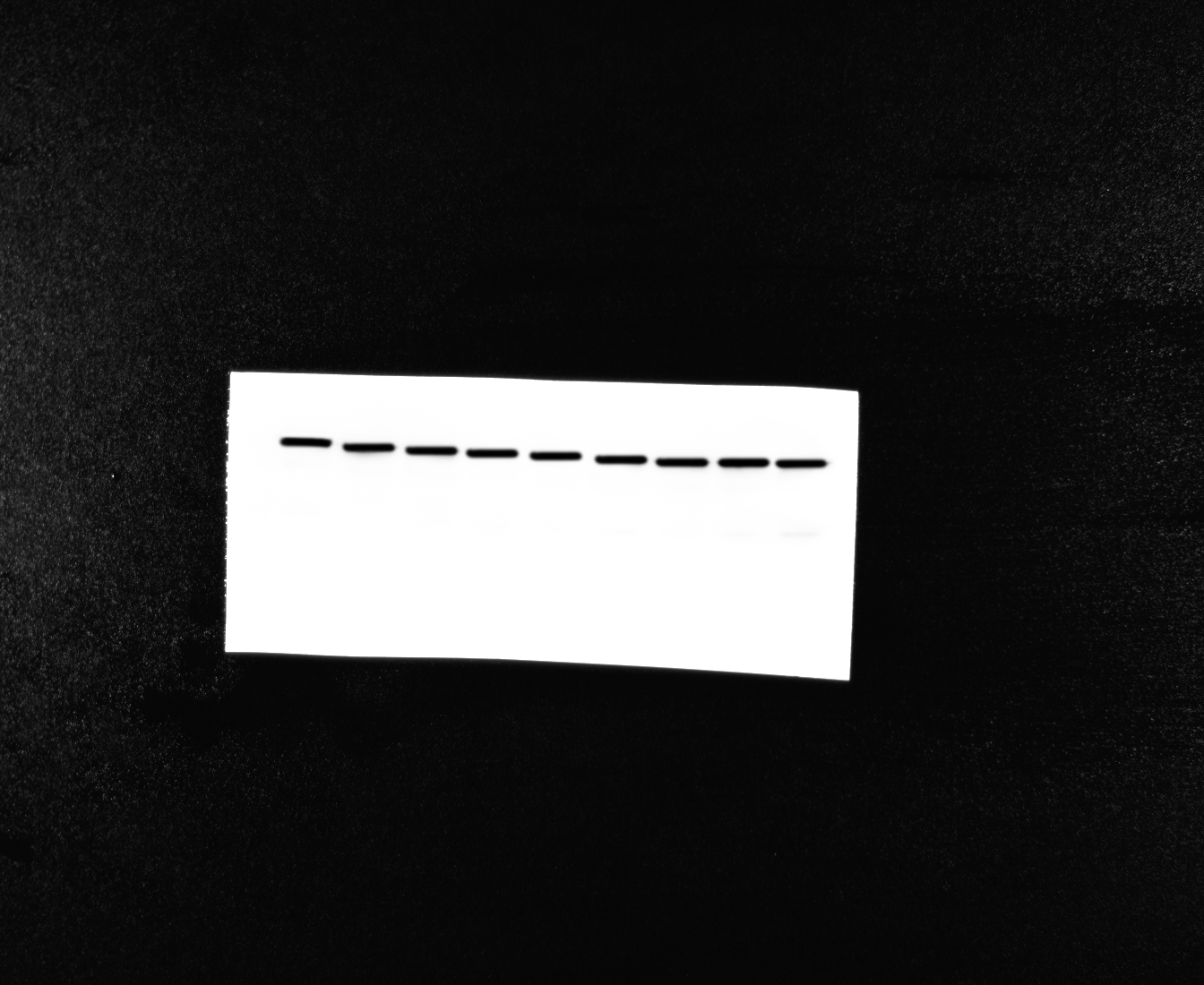

Supplement: Supplementary file 16 [file DataSheet7.zip › Figure 5 A,D-WB bands of ERK and Smad in vivo and vitro/FIGURE5-WB bands in vivo/ERK12,Smad4 β-actin.tif]

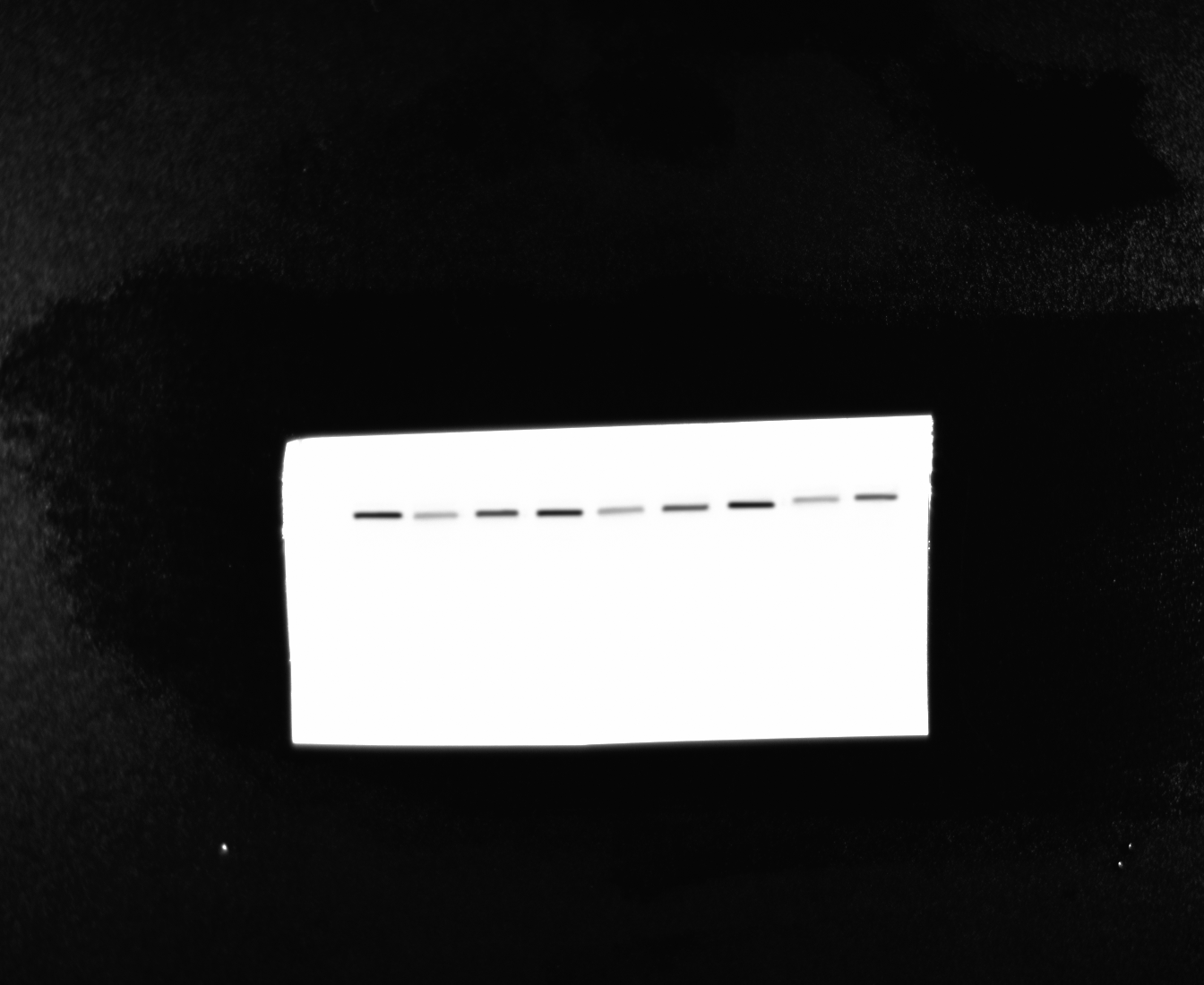

Supplement: Supplementary file 16 [file DataSheet7.zip › Figure 5 A,D-WB bands of ERK and Smad in vivo and vitro/FIGURE5-WB bands in vivo/ERK12.tif]

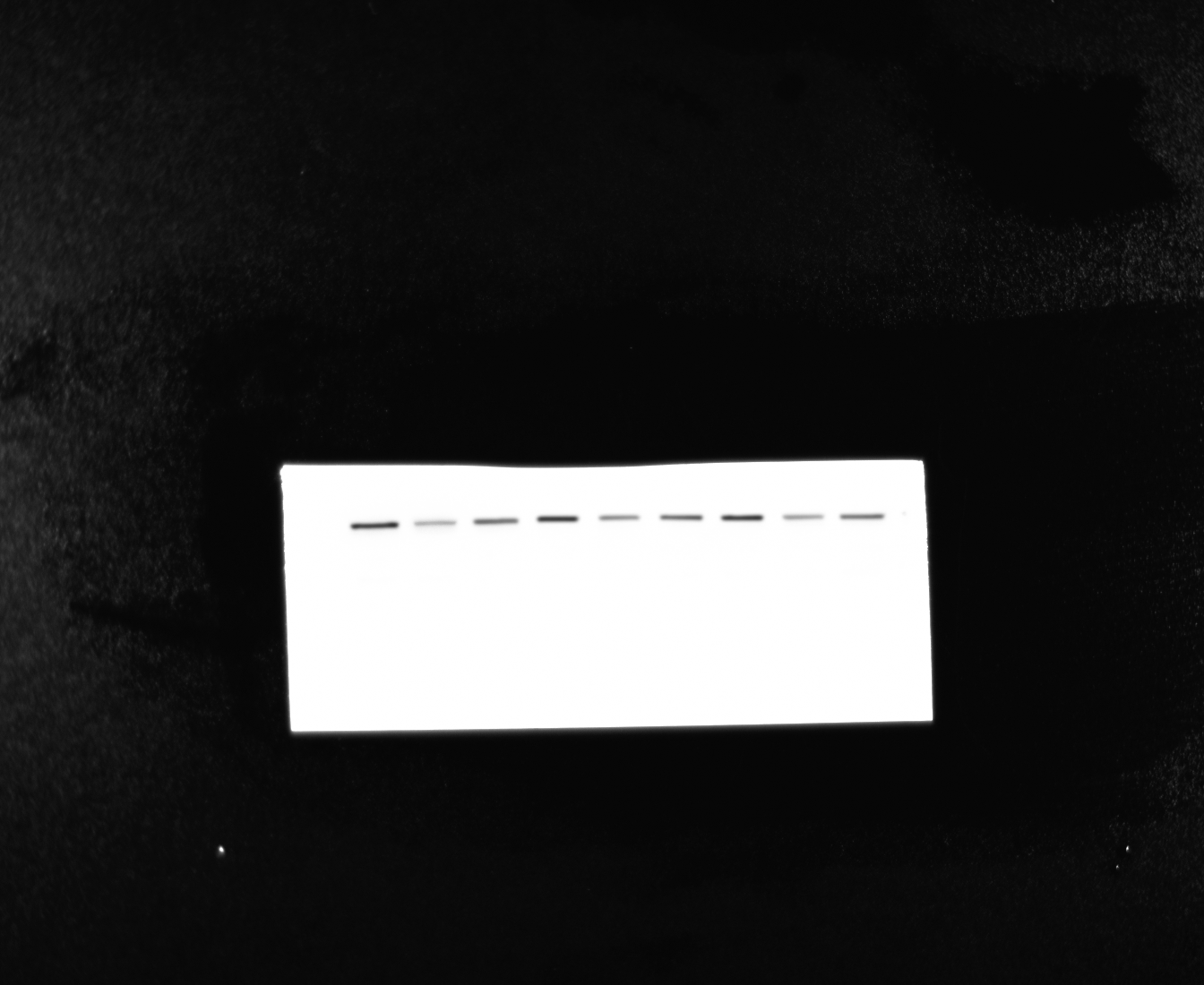

Supplement: Supplementary file 16 [file DataSheet7.zip › Figure 5 A,D-WB bands of ERK and Smad in vivo and vitro/FIGURE5-WB bands in vivo/Smad4.tif]
